# No evidence of genetic causality between diabetes and osteonecrosis: a bidirectional two-sample Mendelian randomization analysis

**DOI:** 10.1186/s13018-023-04428-7

**Published:** 2023-12-16

**Authors:** Wei Li, Jin-Lian Chai, Zhe Li, Cong-Cong Guo, Ran Wei, Tie-Feng Sun, Xue-Zhen Liang

**Affiliations:** 1 College of Traditional Chinese Medicine, Shandong University of Traditional Chinese Medicine, Jinan, 250355 Shandong China; 2 College of Pharmacy, Shandong University of Traditional Chinese Medicine, Jinan, 250355 Shandong China; 3 Department of Cardiology, The First Affiliated Hospital of Shandong First Medical University, Shandong Provincial Qianfoshan Hospital, Jinan, 250000 Shandong China; 4 Department of Endocrinology and Metabology, The First Affiliated Hospital of Shandong First Medical University, Shandong Provincial Qianfoshan Hospital, Jinan, 250000 Shandong China; 5 Science and Technology Department, Affiliated Hospital of Shandong University of Traditional Chinese Medicine, Jinan, 250011 Shandong China; 6 Shandong Provincial Research Institute of Traditional Chinese Medicine, Jinan, 250014 Shandong China; 7 Orthopaedic Microsurgery, Affiliated Hospital of Shandong University of Traditional Chinese Medicine, 16369 Jingshi Road, Jinan, 250014 Shandong China; 8 First College of Clinical Medicine, Shandong University of Traditional Chinese Medicine, Jinan, 250355 Shandong China

**Keywords:** Diabetes, Mendelian randomization, Osteonecrosis, Genome-wide association study, Single-nucleotide polymorphism

## Abstract

**Objective:**

This study aimed to examine whether diabetes mellitus is causally associated with osteonecrosis.

**Method:**

Using publicly accessible genome-wide association study statistics, a bidirectional two-sample Mendelian randomization analysis was carried out. In order to determine whether diabetes has a causal effect on osteonecrosis and whether osteonecrosis has a causal effect on diabetes, we extracted six date on diabetes in Europeans from IEU OpenGWAS and GWAS Catalogue and osteonecrosis in Europeans from FinnGen. We then evaluated the data using inverse variance weighting, MR-Egger regression, weighted median, weighted mode, and simple mode. The results’ stability and dependability were then evaluated using sensitivity analysis and heterogeneity analysis. Finally, meta-analysis is used to further confirm if there is a relationship between diabetes and osteonecrosis.

**Results:**

When diabetes was used as an exposure factor, MR-Egger regression showed that directional fold product was unlikely to bias the results. Cochran’s Q test showed only minor heterogeneity in a few data sets. Multidirectional tests Egger-intercept, MR-PRESSO and funnel plots for most data did not show multidirectional and asymmetry at the gene level. Most of the IVW results showed no causal relationship between diabetes mellitus and osteonecrosis. The results of meta-analysis of IVW methods further confirmed the absence of a causal relationship. Inverse MR analysis also showed no causal relationship between osteonecrosis and diabetes.

**Conclusion:**

Results of bidirectional MR analysis show no evidence of causal relationship between diabetes and osteonecrosis.

**Supplementary Information:**

The online version contains supplementary material available at 10.1186/s13018-023-04428-7.

## Introduction

Osteonecrosis is a common condition that affects the knee and hip joints [1, 2]. Most experts agree that osteonecrosis is primarily caused by blood flow obstructions in the bone, which cause local bone cells to die and bone trabeculae to necrotize, altering the bone structure [3, 4]. Once osteonecrosis manifests, the rate of disability is very high [5], which negatively impacts the patient’s quality of life and significantly burdens the patient’s family and society. Osteonecrosis is a refractory disease that has become commonly observed in orthopaedic clinics [6]. There are many treatment options for osteonecrosis [2], and currently, effective hip preservation therapies include core decompression [7] and osteotomy [8]. However, the risk of surgical treatment increases with age [9, 10]. Osteonecrosis is mainly categorized as traumatic and nontraumatic [6]. Traumatic osteonecrosis is the interruption of blood flow to the bone produced by a variety of traumatic events, the most frequent of which are femoral neck fracture and hip dislocation resulting in femoral head osteonecrosis [11]. Nontraumatic causes of osteonecrosis include corticosteroid use [12], haemoglobinopathies (sickle cell anaemia) [13], fat embolism [14], alcoholism [15], and systemic lupus erythematosus (SLE) [1]. X-rays and bone scans are used to diagnose osteonecrosis with clinical symptoms [16, 17]. Although magnetic resonance imaging (MRI) is the most sensitive diagnostic [1, 18] for detecting early osteonecrosis and silent osteonecrosis, detecting early asymptomatic osteonecrosis remains challenging [16, 19], as its pathogenesis is still not fully elucidated. Evidence suggests that osteonecrosis is linked to various pathogenic pathways, including intravascular coagulation [20], mechanical stress [21], corticosteroid use [12], and primary cell death [22].

Diabetes is categorized into type 1 diabetes and type 2 diabetes mellitus (T2DM) [23]. T2DM is a multifactorial group disease of leukocyte insulin secretion and/or insulin resistance, resulting in disturbances in carbohydrate, lipid and protein metabolism [24]. The most obvious feature of T2DM is insulin resistance in patients with T2DM. T2DM increases the risk of cardiovascular disease and overall mortality [25, 26]. The global prevalence of T2DM has been increasing over the past few decades; it is projected that by 2045, people with T2DM will account for 9.9% of the world population [27–31], resulting in an increasingly unsustainable global health burden [32]. One of the hallmarks of type 1 diabetes (T1DM) is high blood glucose, and it has been shown that people with T1DM have lower bone mineral density, which is a central factor of the increased risk of fractures [33]. A clinical study suggested that diabetes may be linked to osteonecrosis [25, 34].

There are numerous risk factors for osteonecrosis, including known direct causes such as trauma, radiation exposure, sickle cell anaemia, and caisson disease, and indirect causes such as rheumatic and metabolic disorders, glucocorticoid use, alcohol consumption, and smoking [35–38]. Diabetes mellitus, for instance, may have a significant impact on the development of osteonecrosis in people with a genetic predisposition towards osteonecrosis; however, this is still debatable [34, 39, 40]. Wojciech Konarski summarized the evidence from studies that had reported on the occurrence of avascular necrosis (AVN) in sites other than the jaw, depending on the diagnosis of diabetes, using a systematic review and meta-analysis. The results indicated that diabetes could increase the risk of avascular osteonecrosis in sites other than the jaw [34]. A study conducted by Lai et al. in Taiwan also showed that diabetes is a risk factor for osteonecrosis, and people with diabetes had a greater risk of AVN of the femoral head by a factor of 1.16 [41]. However, not all studies on diabetes and osteonecrosis have come to the same conclusion [41–43]. A comprehensive study conducted by Yang et al. in an orthopaedic hospital found that diabetic patients did not have a greater risk of developing AVN than the general population [43]. These studies suggest that diabetes may be a risk factor for osteonecrosis, but the mechanisms and causation of such connections are unknown, and the majority of research that infers relationships is dependent on observational data. However, conclusions about causality cannot be based solely on associations that exist in observational designs because observational studies are susceptible to many confounding factors and reverse causation and are not sufficiently convincing [44, 45]. Therefore, exploring the causality between diabetes and osteonecrosis is crucial.

To address the excess of confounding factors, we used MR analysis. MR has emerged as a powerful method for identifying causal relationships between risk factors and diseases using genetic variation as an instrumental variable [46–48]. In this study, we examined the bidirectional causal association with osteonecrosis for T1DM and T2DM to verify the hypothesis that diabetes increased the incidence of osteonecrosis. We then conducted META analysis of multiple database results to ensure the reliability of the data to explore whether a causal association of diabetes exists with osteonecrosis.

## Materials and methods

### Study design

**Fig. 1:**
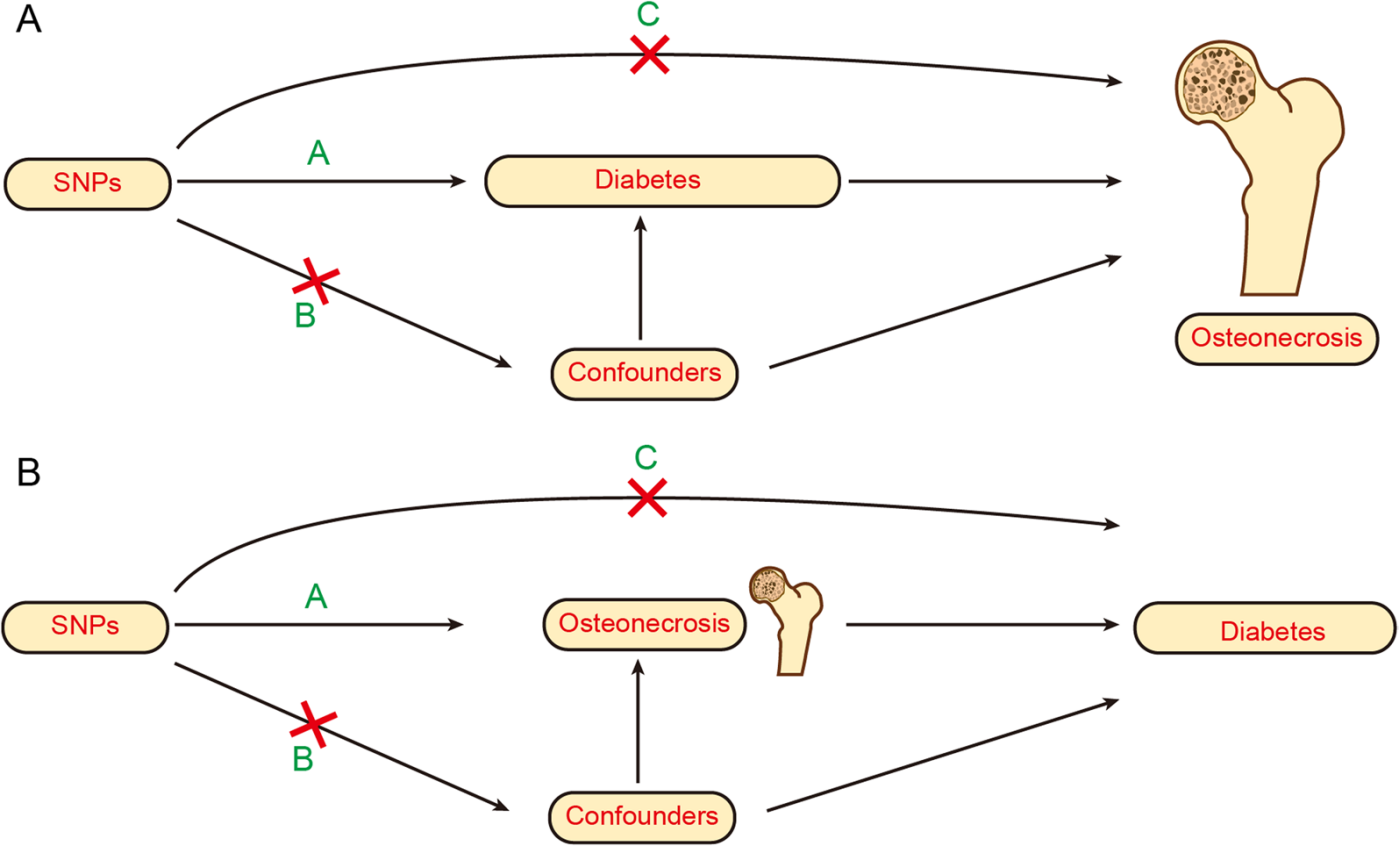
Three key assumptions of the MR study : **A** (①) Relevance assumptions: SNPs are strongly associated with exposure; **B** (②)Independence assumptions: SNPs are independent of confounders; **C** (③) Exclusivity assumption: SNPs must only affect outcome via exposure. *SNPs* single-nucleotide polymorphism

The schematic view of the study design and the three key assumptions of MR, as depicted in Fig. 1, are as follows: ① single-nucleotide polymorphisms (SNPs) are strongly associated with exposure; ② SNPs are independent of known confounders; ③ SNPs affect the outcome only via exposure (Fig. 1).

### Data sources and selection of genetic variants

The study was conducted using the IEU OpenGWAS database (https://gwas.mrcieu.ac.uk/, accessed On 7 August 2023), FinnGen (https://www.finngen.fi/en), and GWAS Catalogue (https://www.ebi.ac.uk/gwas/), encompassing GWASs of the traits of interest in predominantly European individuals and including both males and females. All databases were publicly available GWAS databases. As such, no additional ethical approvals were required. Data on diabetes were obtained through the IEU OpenGWAS database and the GWAS Catalogue database (see Table 1 for specific information). GWAS summary statistics for osteonecrosis were obtained from the FinnGen (https://www.finngen.fi/en) consortium R9 release data [49, 50]. This GWAS included 359,399 Europeans (1835 cases and 358,014 controls) with 20,169,843 SNPs. The sex, age, first 10 principal components, and genotyping batch were corrected during the analysis [50]. All the above populations are of European origin to minimize potential bias due to population heterogeneity. Specific brief information is presented in Table 1.

**Table 1:** Summary of the GWAS included in this TSMR study

Variables	Data codes	Source of sample ethnicity	Sample size	Size of SNPs	Year of publication
T2DM	ebi-a-GCST006867	Europeans	655,666	5,030,727	2018
T2DM	ebi-a-GCST005413	Europeans	70,127	14,277,791	2018
T2DM	GCST90006934	Europeans	22,326	8,919,079	2020
Severe insulin resistant T2DM	GCST90026414	Europeans	3,874	5,397,362	2021
T2DM	GCST90026417	Europeans	12,230	5,399,457	2021
T2DM	GCST90043636	Europeans	456,348	11,842,647	2021
T1DM	ebi-a-GCST005536	Europeans	29,652	101,101	2015
T1DM	ebi-a-GCST010681	Europeans	24,840	12,783,129	2020
T1DM	ebi-a-GCST90000529	Europeans	17,685	7,740,245	2021
T1DM	ebi-a-GCST90014023	Europeans	520,580	59,999,551	2021
T1DM	ebi-a-GCST90018925	Europeans	457,695	24,182,422	2021
Osteonecrosis	R9_M13_OSTEONECROSIS	Europeans	359,399	20,169,843	2021

*SNPs* single-nucleotide polymorphism

### Selection of instrumental variables

To filter eligible genetic instrumental variables (IVs) that fulfil the three core MR assumptions depicted in Fig. 1, we performed a set of quality control techniques. We selected SNPs strongly associated with diabetes at the genome-wide significance threshold of *P* < 5 × 10^−8^. However, screening the IVs according to this threshold yielded only a small number of SNPs, so we used a second threshold, selecting SNPs below the genome-wide significance threshold of *P* < 1 × 10^−5^ and selecting them as IVs to identify more potential causal relationships between osteonecrosis and diabetes. Then, we screened and removed SNPs correlated with confounding factors and outcomes with *r*^2^ > 0.001 to avoid linkage disequilibrium (LD) in the range of 10,000 KB [51]. Afterwards, the potential confounders associated with the selected SNPs were analysed in the PhenScanner V2 database (http://www.phenoscanner. medschl.cam.ac.uk/, accessed on 23 June 2023), focusing on excluding the SNPs whose corresponding phenotypes have relevant significance with the outcome. The *F*-statistic equals [(*n* − *k* − 1)/*k*) × *R*^2^/ (1 − *R*^2^)], where *R*^2^ represents the variance in exposure explained by the genetic instrument, *K* represents the number of genetic variations, and *N* represents the sample size. The *R*^2^ value was calculated as follows: 2 × *β*^2^ × EAF × (1 − EAF)/2 × *β*^2^ × EAF × (1 − EAF) + se^2^ × 2 × *N* × EAF(1 − EAF). EAF represents the effect allele frequency [52]. The *F*-statistic was calculated for each SNP to validate its strength and to estimate the sample overlap effect and weak instrument bias considering the relatively relaxed threshold; *F* > 10 was considered powerful enough to mitigate the influence of potential bias. IVs with *F* statistics *F* < 10 were considered weak instruments and were excluded from MR analysis [53].

Information on the outcome was extracted through the IEU OpenGWAS database, GWAS Catalogue database or FinnGen database, and the relationship between the SNPs satisfying the hypotheses was obtained from the outcome. The exposure and outcome datasets, which contain the relationship between the above IVs and the outcome and exposure, were combined, and the palindromic SNPs were deleted. The last remaining SNPs were the final instrumental variable regarding the exposure.

### Statistical analysis for MR

The bidirectional TSMR analysis and meta-analysis were performed using R software (version 4.1.2, R Foundation for Statistical Computing) with the “Wampler” R package (version 0.5.6) and the “MR-PRESSO” R package (version 1.0.0). Five MR approaches were utilized as sensitivity analyses, including MR-Egger, weighted median, inverse variance weighted, simple mode and weighted mode.

### Heterogeneity and sensitivity test

We used the mr_heterogeneity package to conduct a Cochran’s Q test on the SNPs that fit the full hypothesis to assess heterogeneity among individual genetic variants [54]. If the Cochran’s Q test result is *P* < 0.05, the results are heterogeneous, indicating that the relationship between exposure and outcome is influenced by age and sex. The final MR result refers to the IVW random effect model as the gold standard; otherwise, we used the IVW fixed effect model as the gold standard. We also used the MR pleiotropic test Egger-intercept method and the MR-PRESSO test to verify whether there is a violation of MR assumptions due to horizontal MR. For the Egger-intercept method of horizontal pleiotropy [55], where the cut-off estimates whether genetic variation significantly influences outcome through pathways other than exposure, *P* < 0.05 represents the presence of horizontal pleiotropy, indicating that the selected IVs significantly influence the outcome variables through pathways other than exposure, which violates hypotheses ② and ③ as depicted in Fig. 1. *P* > 0.05 indicates that the outcome variables are not significantly influenced through routes other than exposure. The “leave-one-out” test as a sensitivity analysis indicated whether any of the final SNPs were outliers. We verified whether the results were stable by examining the asymmetry in the funnel plot. We then identified outliers by the MR-PRESSO method and evaluated the effect of outliers on the results [56]. Finally, meta-analysis of the results of the IVW method was performed on all data to enhance the persuasiveness of the experiment.

## Results

We performed a bidirectional TSMR analysis to explore the causal relationship between diabetes and osteonecrosis risk. Our results suggest neither a causal effect of diabetes on osteonecrosis nor a causal effect of osteonecrosis on diabetes.

### Impact of diabetes on osteonecrosis

#### IVs for MR and five methods results

**Fig. 2:**
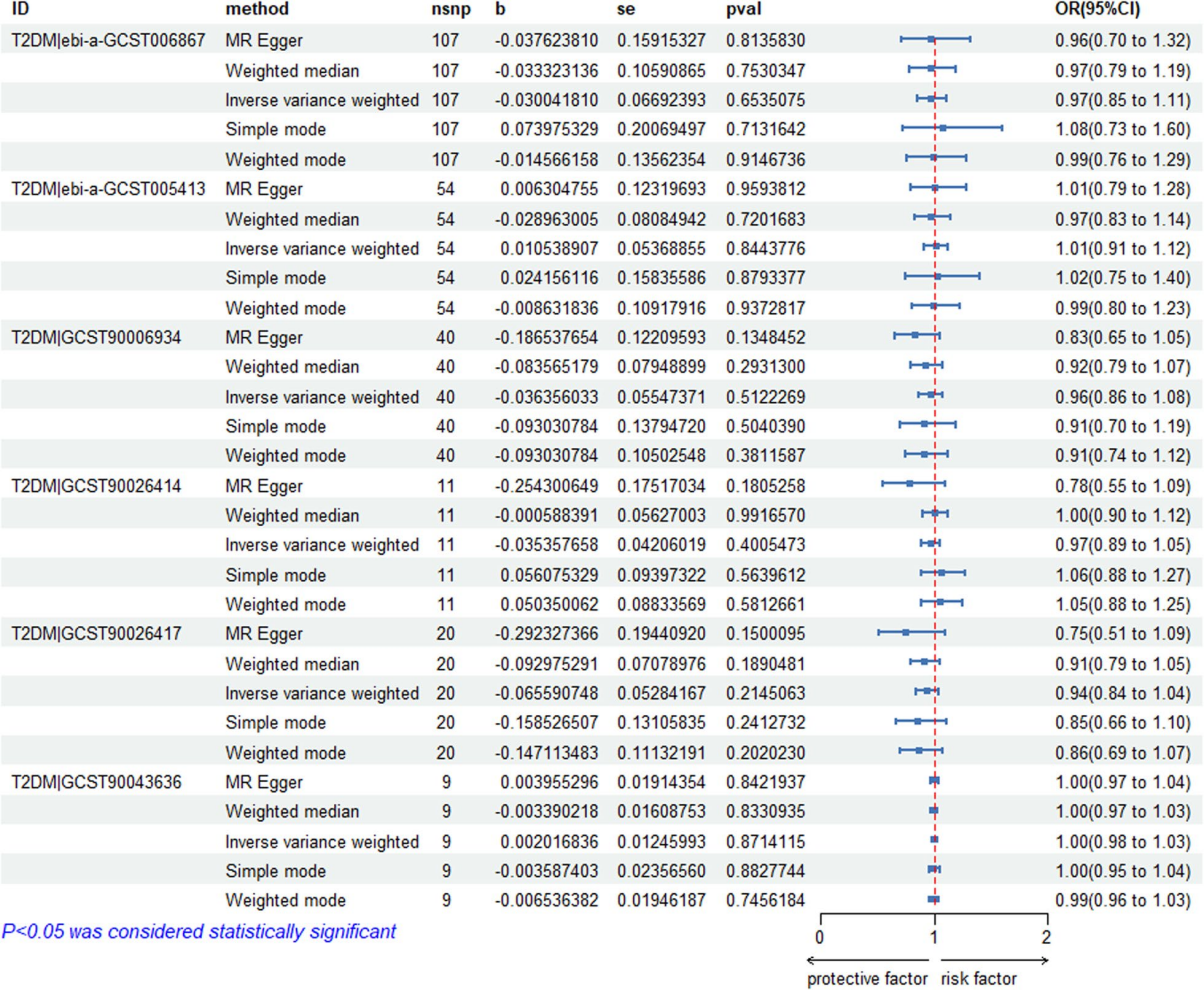
MR analysis of all T2DM data

**Fig. 3:**
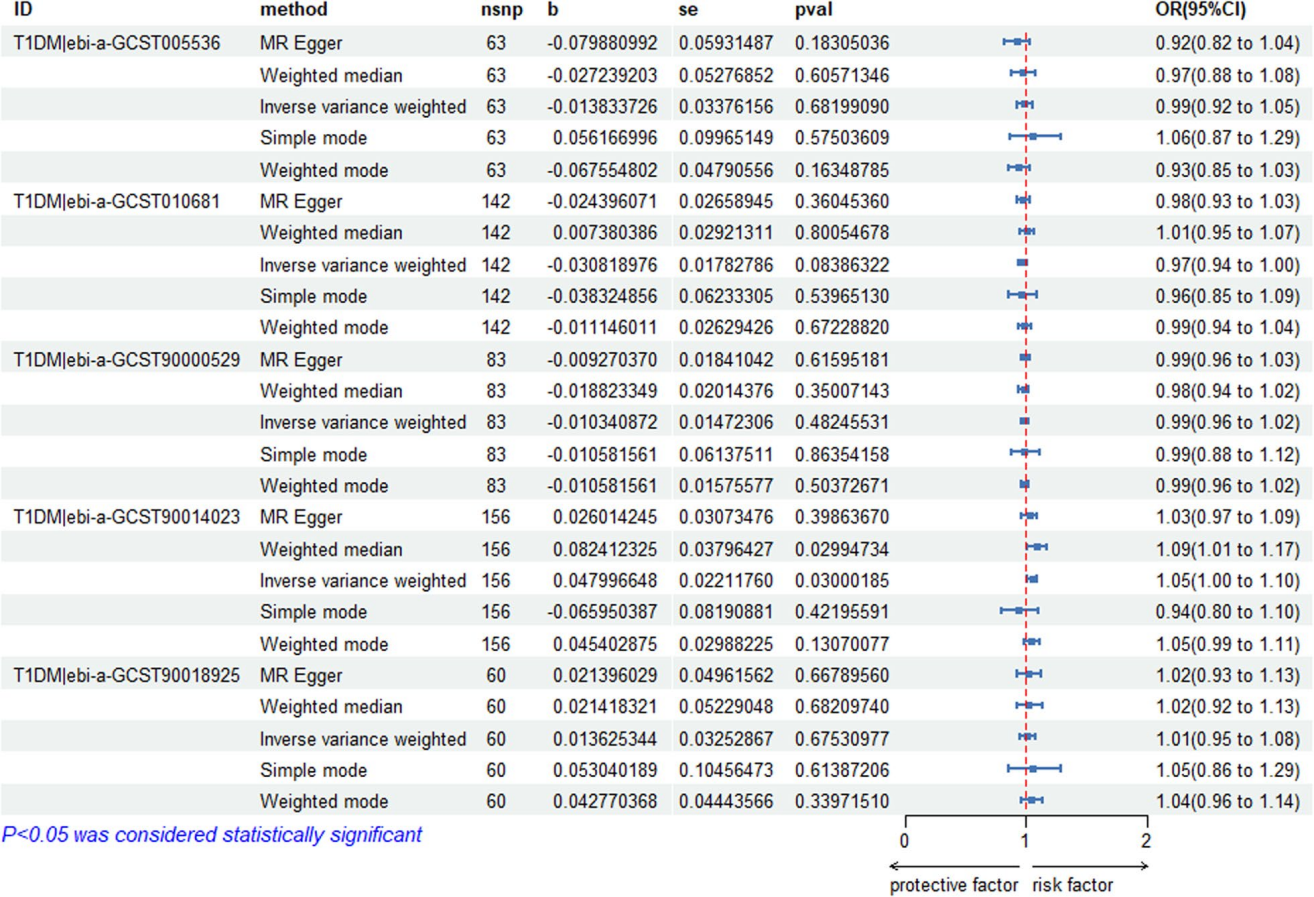
MR analysis of all T1DM data

We selected 118 independent SNPs from the IEU OpenGWAS database (ebi-a-GCST006867) on T2DM as the IVs. The SNP-related phenotypes were retrieved using the PhenoScanner V2 database, primarily excluding the SNPs whose corresponding phenotype was associated with osteonecrosis (*n* = 0). We used the calculation formulas for the *R*^2^ and *F* values to calculate the *F* values of 118 SNPs. All of these *F* values were greater than 10, which demonstrated that 118 IVs were selected as strong IVs in this study. We extracted the outcome information of osteonecrosis through FinnGen and obtained the relationship between the above SNPs and the outcome from the database. We further merged the exposure and outcome datasets, which included the 118 IVs with outcome and exposure and removed the palindrome SNPs. The remaining 107 SNPs were the final instrumental variable for the exposure. Specific information on the data and the results of the MR analyses are provided in Fig. 2, which shows no causal effect of T2DM on osteonecrosis (IVW: *P* > 0.05). Figure 3 shows that T1DM|ebi-a-GCST90014023 was positively correlated with osteonecrosis (IVW: *P* < 0.05, OR > 1), and MR analysis of the rest of the T1DM data on osteonecrosis showed no causal relationship (IVW: *P* > 0.05). Additional file 1 contains instrumental variable SNPs for all data.

#### Heterogeneity and sensitivity test

Heterogeneity [57] is the variability in the causal estimates obtained for each SNP. Low heterogeneity suggests increased reliability of MR estimates. As shown in Table 2, heterogeneity was exhibited when we chose ebi-a-GCST006867 (T2DM) as the exposure factor (*P* = 0.023376 < 0.05). The remaining databases were not heterogeneous. Then, we used a random effects model to estimate the effect size of MR: IVW (*β* = -0.03, SE = 0.067, *P* = 0.654, OR = 0.97, CI: 0.85–1.11), as shown in Table 3. The results of the horizontal multivariate tests are depicted in Table 2. These results show that the IVs from all databases did not significantly affect the results through pathways other than exposure, as indicated by the Egger-intercept method. The leave-one-out sensitivity analysis indicated that the absence of a single SNP had little effect on the causal estimate of diabetes on osteonecrosis risk (see Additional file 2). The MR-Egger regression test, MR-PRESSO test and funnel plot exhibited favourable symmetry and showed no evidence of horizontal pleiotropy (see Additional file 2).

**Table 2:** MR sensitivity analysis

Exposure	Outcome	Inverse variance weighted			Egger-intercept method		
		Q	df	pval	Intercept	Se	pval
T2DM|ebi-a-GCST006867	Osteonecrosis	136.87	106	0.023376	0.000599	0.011389	0.958181
T2DM|ebi-a-GCST005413	Osteonecrosis	58.29	53	0.287162	0.000591	0.015444	0.969617
T2DM|GCST90006934	Osteonecrosis	35.63	39	0.624461	0.025642	0.018571	0.175415
T2DM|GCST90026414	Osteonecrosis	2.29	8	0.970642	− 0.004402	0.033005	0.897648
T2DM|GCST90026417	Osteonecrosis	6.32	19	0.997049	0.046037	0.037987	0.241215
T2DM|GCST90043636	Osteonecrosis	9.27	10	0.506353	0.089844	0.069779	0.230023
Osteonecrosis	T2DM|ebi-a-GCST006867	10.38	5	0.064952	0.016064	0.018001	0.422631
Osteonecrosis	T2DM|ebi-a-GCST005413	8.00	13	0.843280	0.000131	0.009208	0.988823
Osteonecrosis	T2DM|GCST90006934	16.01	14	0.312732	− 0.003945	0.013111	0.768242
Osteonecrosis	T2DM|GCST90026414	7.52	6	0.274852	− 0.109841	0.201825	0.609660
Osteonecrosis	T2DM|GCST90026417	16.78	6	0.010129	− 0.036664	0.190863	0.855224
Osteonecrosis	T2DM|GCST90043636	6.83	13	0.910530	− 0.013187	0.090997	0.887184
T1DM|ebi-a-GCST005536	Osteonecrosis	67.64	62	0.290772	0.014023	0.010387	0.181985
T1DM|ebi-a-GCST010681	Osteonecrosis	134.07	141	0.647964	− 0.002389	0.00733	0.745224
T1DM|ebi-a-GCST90000529	Osteonecrosis	88.79	82	0.285089	− 0.000662	0.006769	0.922240
T1DM|ebi-a-GCST90014023	Osteonecrosis	160.31	155	0.368407	0.005564	0.005403	0.304699
T1DM|ebi-a-GCST90018925	Osteonecrosis	46.82	59	0.874183	− 0.00208	0.010029	0.836413
Osteonecrosis	T1DM|ebi-a-GCST005536	None	None	None	None	None	None
Osteonecrosis	T1DM|ebi-a-GCST010681	20.91	14	0.103741	0.020082	0.015770	0.225186
Osteonecrosis	T1DM|ebi-a-GCST90000529	21.08	11	0.032524	0.061855	0.024800	0.031768
Osteonecrosis	T1DM|ebi-a-GCST90014023	32.49	14	0.003405	0.015989	0.012557	0.225222
Osteonecrosis	T1DM|ebi-a-GCST90018925	8.10	14	0.883871	0.002244	0.010742	0.837731

**Table 3:** Results of IVW random effects model analysis

Exposure	Outcome	Method	nsnp	b	Se	pval	OR (95%Cl)
T2DM|ebi-a-GCST006867	Osteonecrosis	IVW	107	0.03	0.067	0.654	0.97(0.85 to 1.11)

**Fig. 4:**
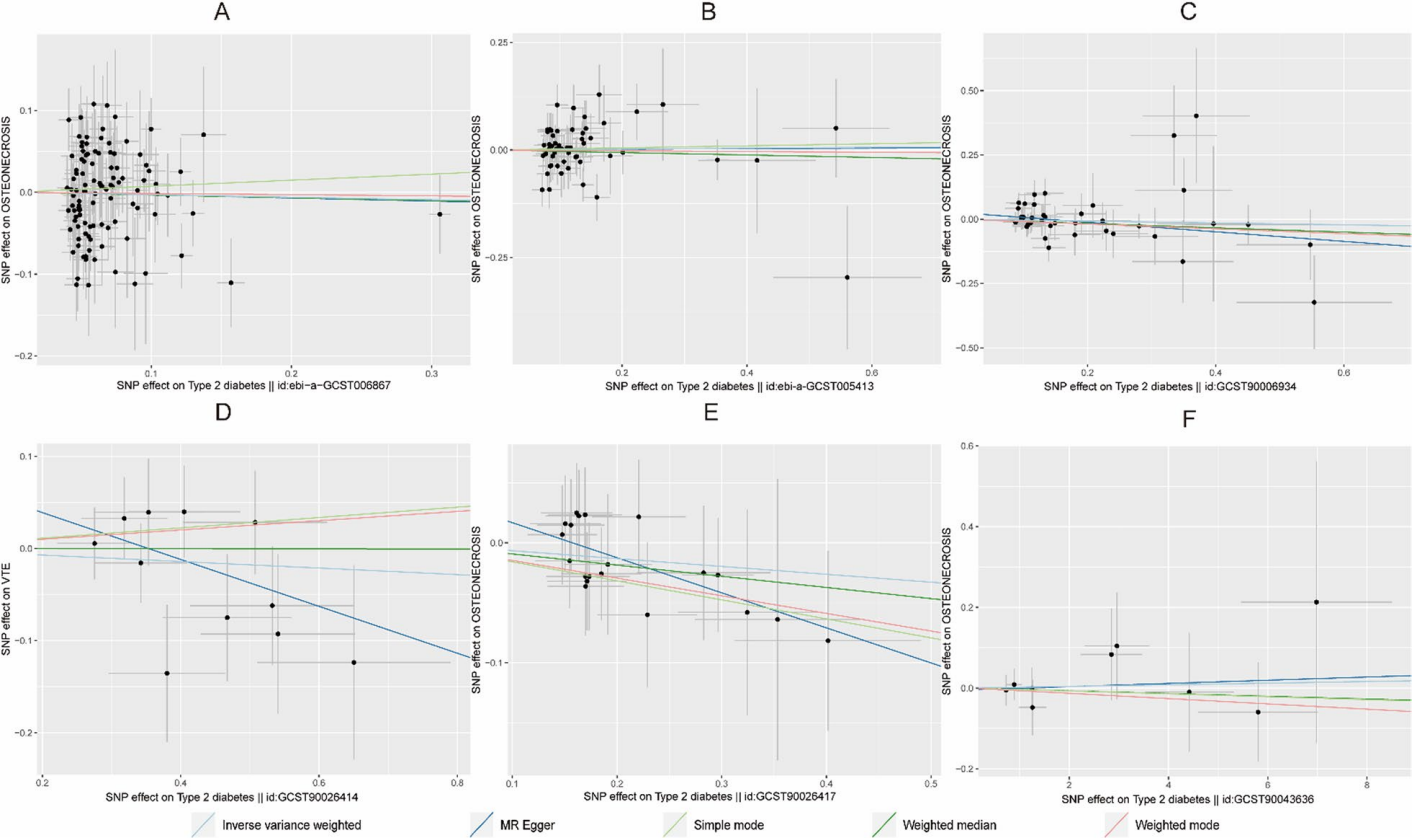
Scatter plot of the causal relationships between osteonecrosis to T2DM levels using different MR methods. **A**: The effect of T2DM|ebi-a-GCST006867 on osteonecrosis; **B**: The effect of T2DM|ebi-a-GCST005413 on osteonecrosis; **C**: The effect of T2DM|GCST90006934 on osteonecrosis; **D**: The effect of T2DM|GCST90026414 on osteonecrosis; **E**: The effect of T2DM|GCST90026417 on osteonecrosis; **F**: The effect of T2DM|GCST90043636 on osteonecrosis

**Fig. 5:**
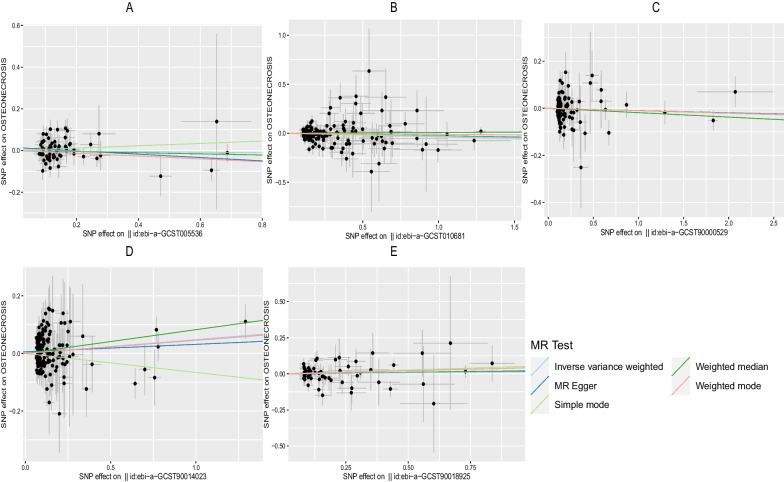
Scatter plot of the causal relationships between osteonecrosis to T1DM levels using different MR methods.  **A**: The effect of T1DM|ebi-a-GCST005536 on osteonecrosis; **B**: The effect of T1DM|ebi-a-GCST010681 on osteonecrosis; **C**: The effect of T1DM|ebi-a-GCST90000529 on osteonecrosis; **D**: The effect of T1DM|ebi-a-GCST90014023 on osteonecrosis; **E**: The effect of T1DM|ebi-a-GCST90018925 on osteonecrosis

Table 3 shows that no evidence was present to support a causal relationship between T2DM (ebi-a-GCST006867) and osteonecrosis by the IVW random effects model method (*β* = -0.03, SE = 0.067, *P* = 0.654, OR = 0.97, CI: 0.85–1.11) (Figs. 4, 5).

#### Meta-analysis of IVW methods

**Fig. 6:**
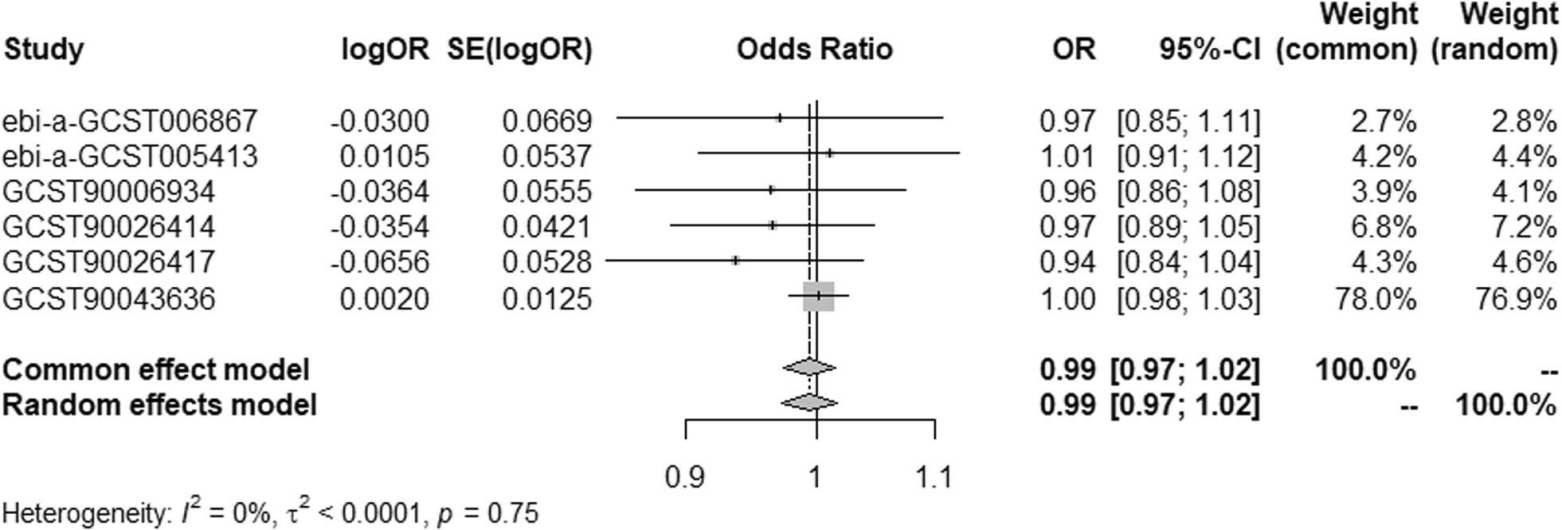
Meta-analysis of IVW results in T2DM

**Fig. 7:**
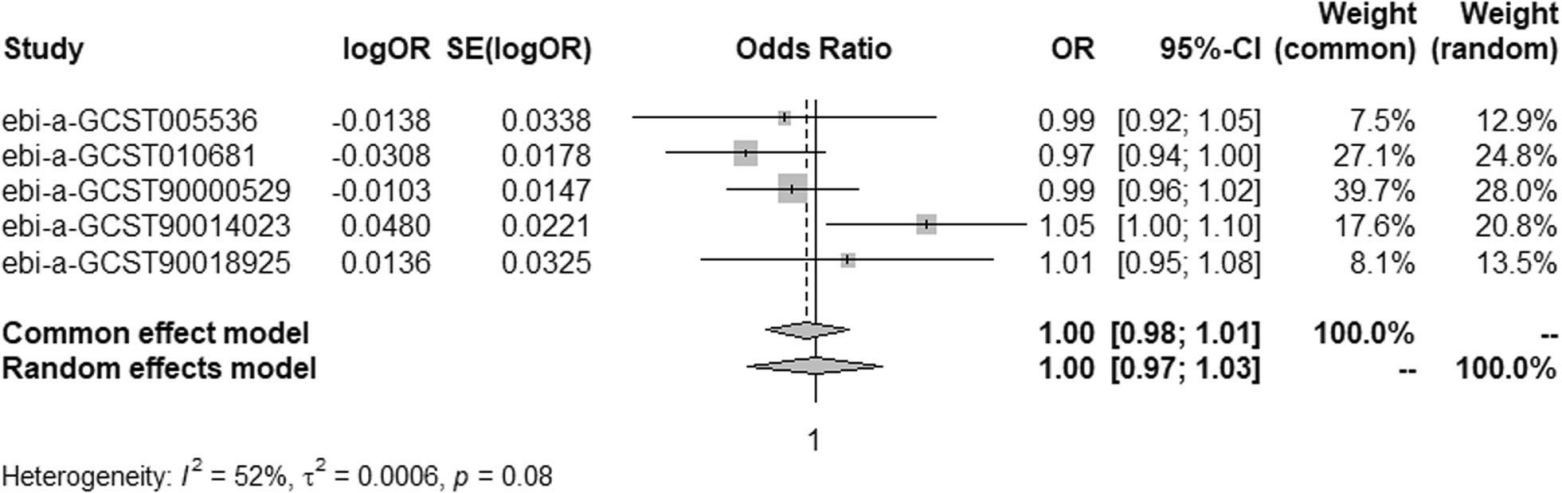
Meta-analysis of IVW results in T1DM

To ensure data reliability, we conducted a meta-analysis of all database results of the IVW method, the specific results of which are shown in Fig. 6. The results of the meta-analysis confirmed that there was no causal association of T2DM with osteonecrosis because the combined confidence (common effect model:0.97–1.02) intervals crossed the null line (OR = 1). There is also no causal association of T1DM on osteonecrosis in the results shown in Fig. 7.

### Impact of osteonecrosis on diabetes

#### IVs for MR and five methods results

**Fig. 8:**
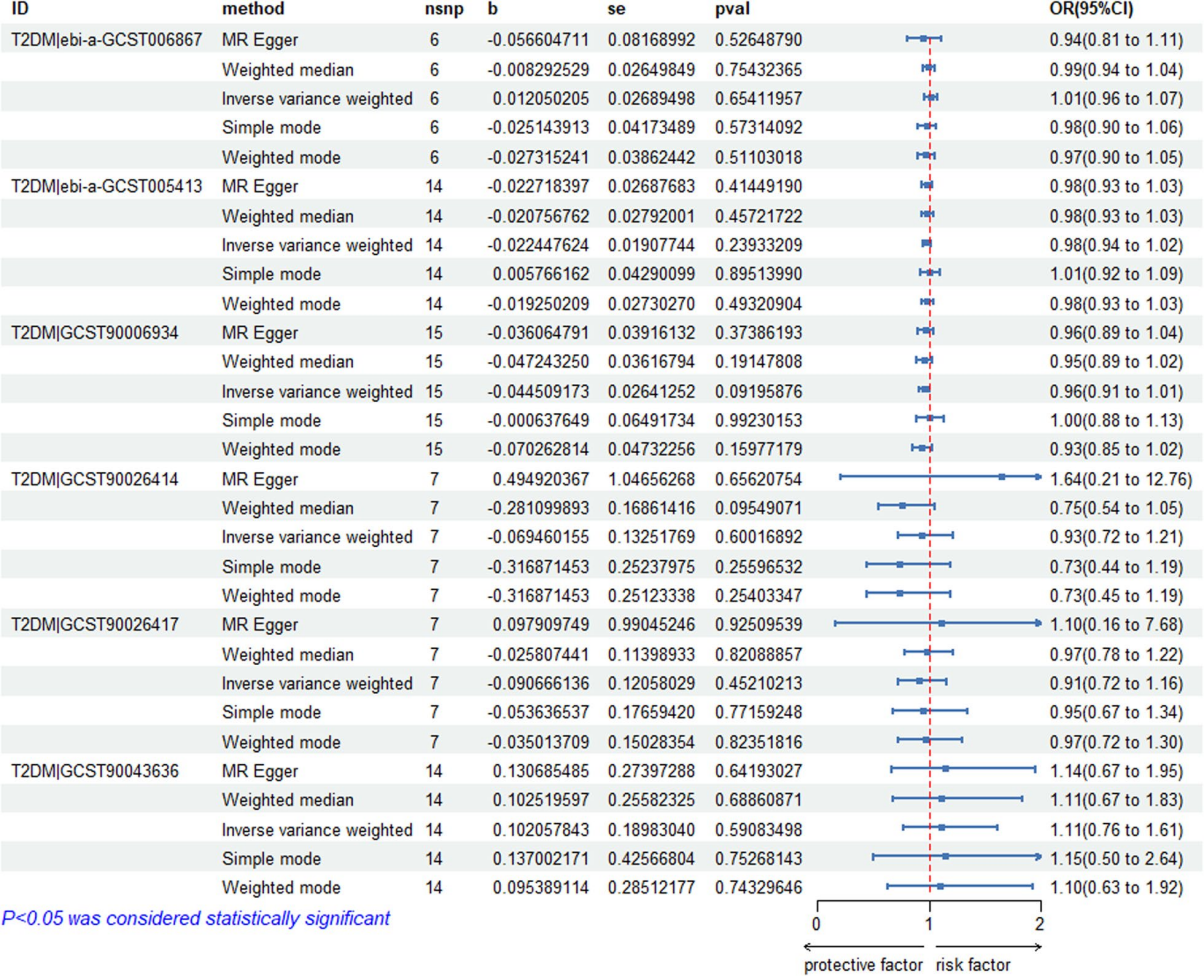
MR analysis of all T2DM data

**Fig. 9:**
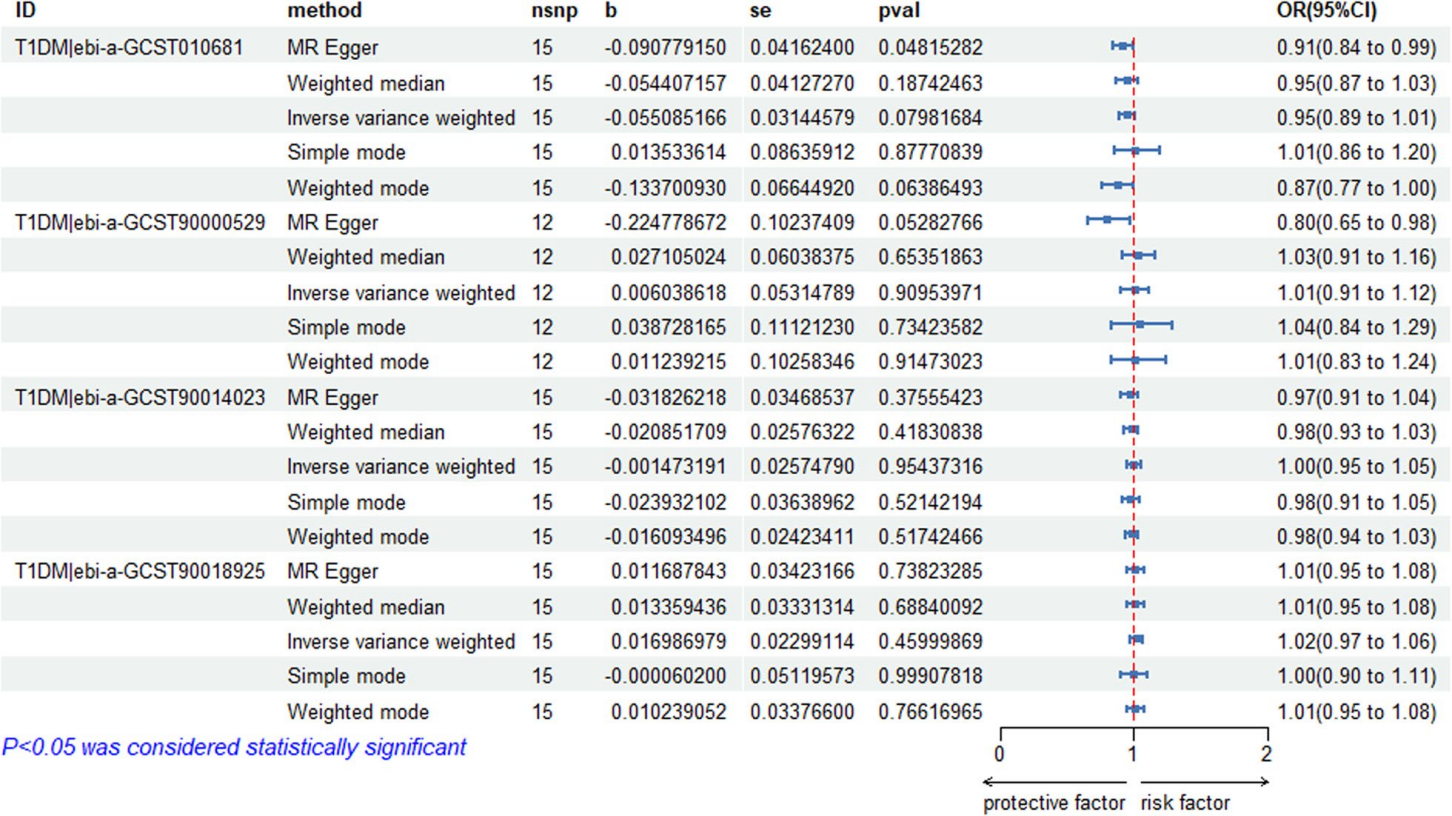
MR analysis of all T1DM data

We selected 20 independent SNPs from the FinnGen database on osteonecrosis as the IVs. The SNP-related phenotypes were retrieved using the PhenoScanner V2 database, primarily excluding the SNPs whose corresponding phenotype was associated with diabetes (*n* = 0). We used the calculation formulas of *R*^2^ and *F* values to calculate the *F* values of 20 SNPs. All of these *F* values were greater than 10, demonstrating that 20 IVs were selected as strong IVs in this study. We extracted the outcome information of diabetes through the IEU OpenGWAS and GWAS Catalogue databases and obtained the relationship between the above SNPs and the outcome from the database. We further merged the exposure and outcome datasets and removed the palindrome SNPs. The remaining SNPs were the final IVs for the exposure. Specific information on the data and the MR analysis results are provided in Fig. 8, which shows no causal effect of osteonecrosis on T2DM (IVW: *P* > 0.05). Figure 9 shows that there is no causal effect of T1DM on osteonecrosis (IVW: *P* > 0.05). Additional file 3 contains instrumental variable SNPs for all data.

#### Heterogeneity and sensitivity test

As shown in Table 2, heterogeneity was exhibited when we chose GCST90026417 (T2DM), ebi-a-GCST90000529 and ebi-a-GCST90014023(T1DM) as the outcome factor (*P* ≤ 0.05). The remaining databases were not heterogeneous. We then used a random effects model to estimate the effect size of MR as shown in Table 4. The Egger’s intercept test in Table 2 showed that there was horizontal pleiotropy when ebi-a-GCST90000529 (T1DM) was used as an outcome factor, but the MR-PRESSO test showed that there was no horizontal pleiotropy (*P* = 0.0778 > 0.05) [56], and that the IVs in the remaining databases did not have a significant influence, as shown by the Egger-intercept method. The leave-one-out sensitivity analysis indicated that the absence of a single SNP had little effect on the causal estimate of osteonecrosis on diabetes risk (see Additional file 4). The MR-Egger regression test, MR-PRESSO test, and funnel plot exhibit favourable symmetry and show no evidence of horizontal pleiotropy (see Additional file 4).

**Table 4:** Results of IVW random effects model analysis

Exposure	Outcome	Method	nsnp	b	Se	pval	OR (95%Cl)
Osteonecrosis	T2DM|GCST90026417	IVW	7	− 0.09	0.121	0.452	0.91(0.72 to 1.16)
Osteonecrosis	T1DM|ebi-a-GCST90000529	IVW	12	0.006	0.053	0.909	1.01(0.91 to 1.12)
Osteonecrosis	T1DM|ebi-a-GCST90014023	IVW	15	− 0.001	0.026	0.954	0.99(0.95 to 1.05)

**Fig. 10:**
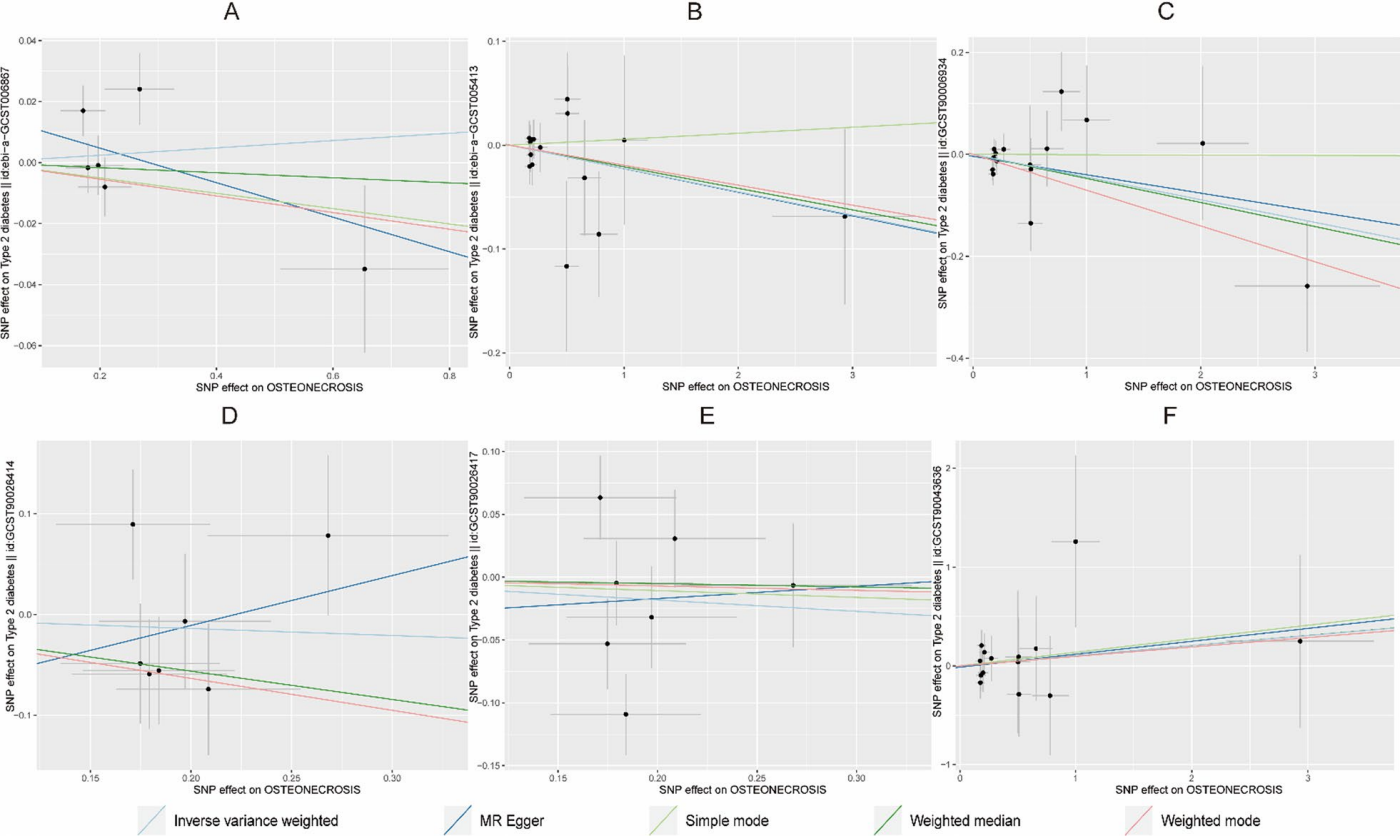
Scatter plot of the causal relationships between osteonecrosis to T2DM levels using different MR methods. **A**: The effect of osteonecrosis on T2DM|ebi-a-GCST006867; **B**: The effect of osteonecrosis on T2DM|ebi-a-GCST005413; **C**: The effect of osteonecrosis on T2DM|GCST90006934; **D**: The effect of osteonecrosis on T2DM|GCST90026414; **E**: The effect of osteonecrosis on T2DM|GCST90026417; **F**: The effect of osteonecrosis on T2DM|GCST90043636

**Fig. 11:**
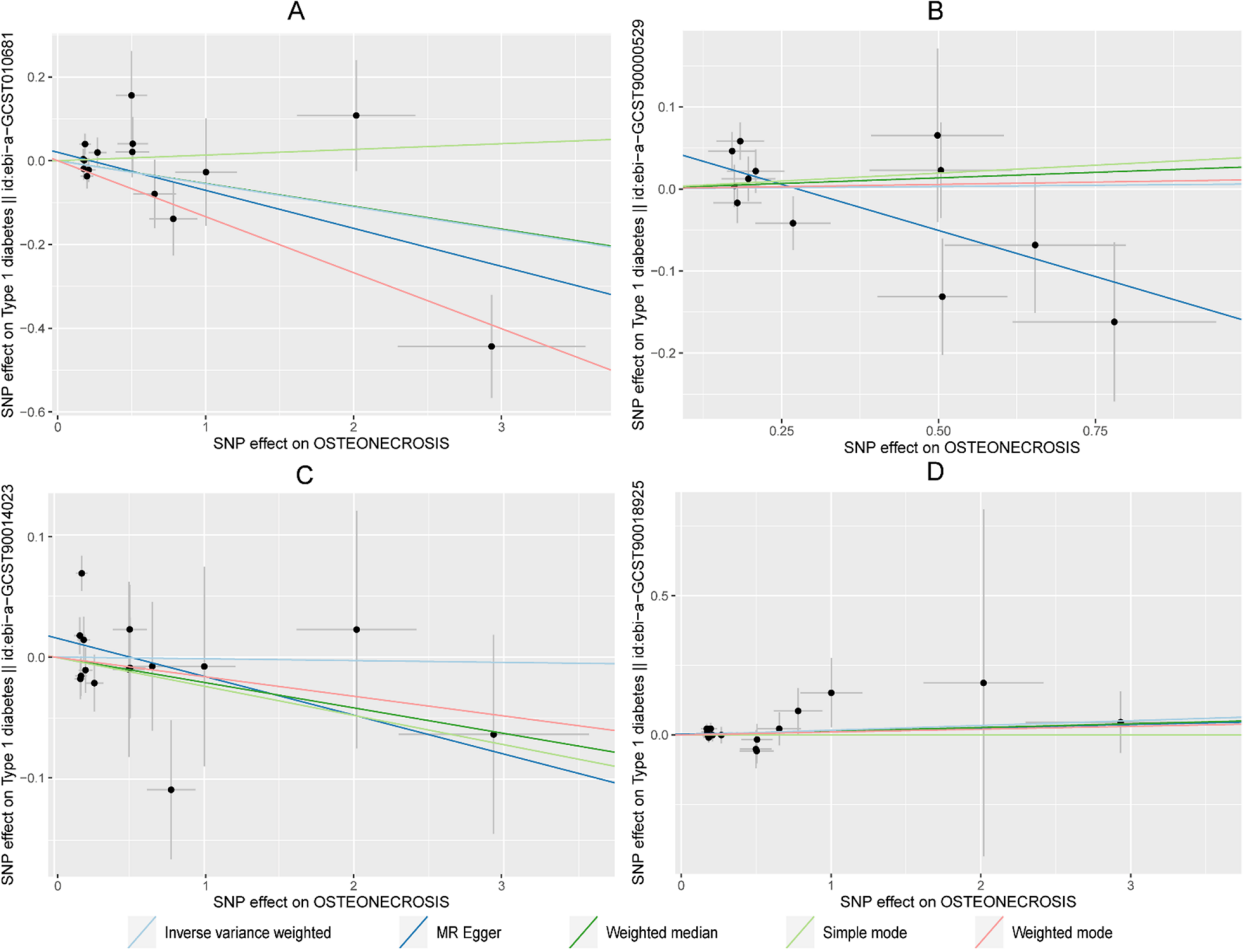
Scatter plot of the causal relationships between osteonecrosis to T1DM levels using different MR methods. **A**: The effect of osteonecrosis on T1DM|ebi-a-GCST010681; **B**: The effect of osteonecrosis on T1DM|ebi-a-GCST90000529; **C**: The effect of osteonecrosis on T1DM|ebi-a-GCST90014023; **D**: The effect of osteonecrosis on T1DM|ebi-a-GCST90018925

Table 4 shows that no evidence supporting a causal relationship between osteonecrosis and T2DM (GCST90026417) was present using the IVW random effects model method (*β* = -0.09, SE = 0.121, *P* = 0.452, OR = 0.91, CI: 0.72–1.16). There was also no evidence of a causal association of osteonecrosis on T1DM (ebi-a-GCST90000529 and ebi-a-GCST90014023) (Figs. 10, 11).

#### Meta-analysis of IVW methods

**Fig. 12:**
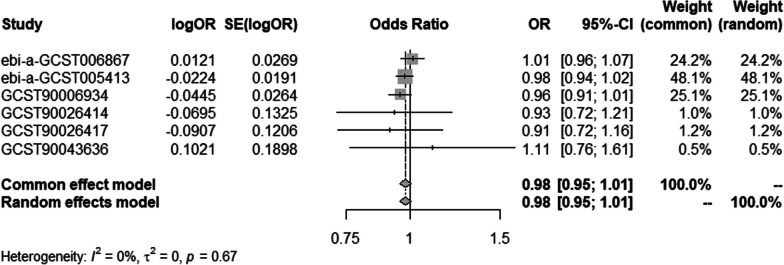
Meta-analysis of IVW results in T2DM

**Fig. 13:**
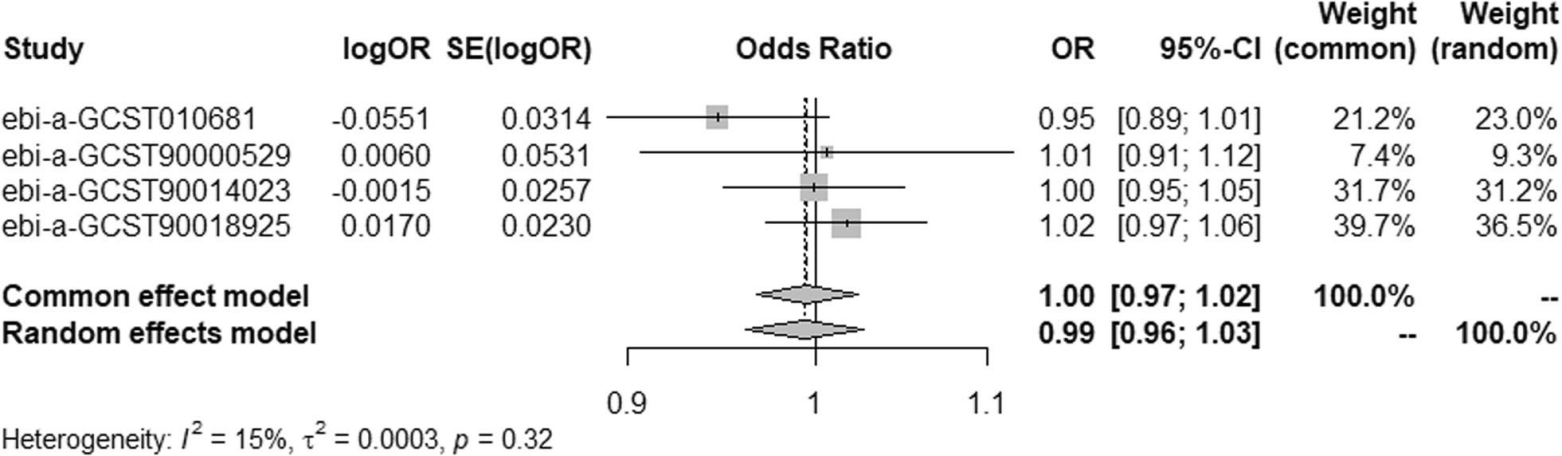
Meta-analysis of IVW results in T1DM

To ensure data reliability, we conducted a meta-analysis of all database results of the IVW method, the specific results of which are depicted in Fig. 12. The results of the meta-analysis confirmed that there was no causal association of osteonecrosis with T2DM because the combined confidence intervals (common effect model:0.95–1.01) crossed the null line (OR = 1). There is also no causal association of T1DM on osteonecrosis shown in Fig. 13.

## Discussion

This study is the first to comprehensively examine the causal effect of diabetes on osteonecrosis using a summary of GWAS data. Our results showed that none of the genetically predicted diabetes cases were significantly associated with the risk of osteonecrosis. The findings from our MR study, which is less prone to confounding than observational studies, did not support the hypothesis that diabetes increases the risk of osteonecrosis.

This study is the first to investigate the potential causal relationship between diabetes and osteonecrosis using a bidirectional TSMR approach, which has a great advantage over observational studies because the genetic variants are all measurable and are not affected by the external environment [46–48]. We set three major hypotheses to ensure that research is not influenced by confounding factors: ① single-nucleotide polymorphisms (SNPs) are strongly associated with exposure; ② SNPs are independent of known confounders; and ③ SNPs affect outcome only via exposure. As long as these three assumptions are satisfied, we can assume that IVs can be substituted for exposure factors [58]. In this study, we let all IVs satisfy *P* < 1 × 10–5 and *F* > 10. All IVs were corrected for using the Bonferroni correction [59], so we could assume that all the IVs satisfy hypothesis ①. SNPs associated with outcome were also eliminated through the PhenoScanner database to fulfil hypothesis ② (*n* = 0). Finally, we performed a sensitivity analysis on the results of the bidirectional MR to satisfy hypothesis ③.

In this study, we examined heterogeneity by Cochran’s Q test, gene-level pleiotropy by Egger’s intercept method and exclusion sensitivity by the leave-one-out method. When ebi-a-GCST006867 was used as the exposure factor or GCST90026417 was used as the outcome factor, Cochran’s Q test *P* < 0.05 indicated heterogeneity was present. However, the heterogeneity was small, so we used an IVW random effects model to analyse the MR effect size [60]. Heterogeneity was allowed because heterogeneity may arise from different analytic platforms, experiments, population stratification, etc. [61]. Random effects modelling allows MR analysis to be conducted in the presence of heterogeneity.

To further explore whether there is a causal effect of T2DM on osteonecrosis, we chose data on T2DM with strong insulin resistance from a genome-wide association study of diabetes by Mansour Aly D et al. (GCST90026414). Insulin resistance is the most obvious manifestation of T2DM. The results still indicated that no causal relationship with osteonecrosis existed. Finally, we used meta-analysis to integrate the data processing of the IVW method of MR analysis. The results still showed that no causal relationship existed between T2DM and osteonecrosis which contradicts the conclusions of previous observational studies [34, 41]. Inverse Mendelian randomization studies showed no causal effect of osteonecrosis on T2DM. In addition, we tested the association between T1DM SNPs and osteonecrosis to assess whether hyperglycaemia was associated with osteonecrosis. The results showed that one T1DM data showed a positive association with osteonecrosis, but the results of meta-analysis by IVW method showed no causal association between hyperglycaemia and osteonecrosis in T1DM. This is contrary to the results of our previous observational study.

The main strength of our study is that we used a bidirectional TSMR design, which reduces bias caused by confounders and reverses causality. Finally, all participants in our exposure-outcome GWAS dataset were of European origin, which avoids bias due to ethnic stratification. Although heterogeneity exists when T2DM (ebi-a-GCST006867) is used as an exposure factor, this heterogeneity was allowed because of factors such as population stratification. This study has some limitations. First, all GWAS data were derived from European populations, and whether the results we derived are applicable to other populations remain to be investigated. Second, although we used different estimation models and rigorous sensitivity analyses to ensure the reliability and robustness of our results, we were unable to completely eliminate heterogeneity and gene-level pleiotropy. This may be due to the complexity and ambiguity of the biological functions of many genetic variants as well as environmental confounders, such as age and gender, which may also impact MR analysis. Finally, when *P* < 5 × 10^−8^ was used as a screening condition, the exposure factor did not produce enough IVs, so this threshold was lowered to *P* < 1 × 10^−5^, but this resulted in insufficiently strong correlation of IVs with the exposure factor. Additionally, additional research in stratified groups (e.g. based on age, sex, ethnicity) is needed to more thoroughly explore the variations in how diabetes affects osteonecrosis in various communities [61].

## Conclusion

In conclusion, our MR study and meta-analysis demonstrated that no causal relationship exists between diabetes and osteonecrosis risk. In addition, there was also no causal relationship regarding the genetic predicted risk of osteonecrosis on the causality of diabetes. The associations shown in previous observational studies may be caused by unmeasured confounders. To validate our findings, large-scale GWAS summarizing data and more recent MR analyses of genetic tools are needed.

### Supplementary Information

**Additional file 1**. Forward MR instrumental variables.

**Additional file 2**. Leave one out and funnel plots of the results of the forward MR analyses of each data for T2DM and T1DM.

**Additional file 3**. Reverse MR instrumental variables.

**Additional file 4**. Leave one out and funnel plots of the results of the r everse MR analyses of each data for T2DM and T1DM.

## Data Availability

Publicly available datasets were analysed in this study. These datasets can be found at the following URLs: IEU OpenGWAS database (https://gwas.mrcieu.ac.uk/); GWAS Catalogue database (GWAS Catalogue (ebi.ac.uk);FINNGEN (https://www.finngen.fi/en).

## Abbreviations

AVN: Avascular necrosis
IVs: Instrumental variables
IVW: Inverse variance weighting
GWAS: Genome-Wide Association Study
LD: Linkage disequilibrium
MRI: Magnetic resonance imaging
SNPS: Single-nucleotide polymorphisms
SLE: Systemic lupus erythematosus
TSMR: Two sample Mendelian randomization
T2DM: Type 2 diabetes mellitus
T1DM: Type 1 diabetes
