# Supplementary material for: No evidence of genetic causality between diabetes and osteonecrosis: a bidirectional two-sample Mendelian randomization analysis
Source: J Orthop Surg Res. 2023 Dec 16;18:970. doi: 10.1186/s13018-023-04428-7 (PMC10725608; doi:10.1186/s13018-023-04428-7)
Supplement: Supplementary file 1 — Additional file 1. Forward MR instrumental variables. [file 13018_2023_4428_MOESM1_ESM.pdf]

# MR instrumental variables

**Table 1:** ebi-a-GCST006867

| SNP | b | se | p | lo_ci | up_ci | or | or_lci95 | or_uci95 |
|---|---|---|---|---|---|---|---|---|
| rs10077431 | -0.22182 | 1.027801 | 0.829129 | -2.23631 | 1.79267 | 0.80106 | 0.106852 | 6.005468 |
| rs10087241 | -2.22107 | 0.842078 | 0.008349 | -3.87155 | -0.5706 | 0.108493 | 0.020826 | 0.565186 |
| rs10100265 | -0.43033 | 0.783686 | 0.582926 | -1.96636 | 1.105691 | 0.650292 | 0.139966 | 3.021312 |
| rs10114341 | -0.54121 | 0.943768 | 0.566335 | -2.391 | 1.308574 | 0.582043 | 0.091539 | 3.700894 |
| rs10401969 | 0.501321 | 0.851569 | 0.55606 | -1.16775 | 2.170397 | 1.650901 | 0.311065 | 8.761758 |
| rs1050226 | -0.55908 | 0.801149 | 0.485276 | -2.12933 | 1.011176 | 0.571737 | 0.118917 | 2.748832 |
| rs1061813 | 0.080747 | 0.916338 | 0.929782 | -1.71528 | 1.87677 | 1.084097 | 0.179914 | 6.53237 |
| rs1063355 | 0.687075 | 0.538626 | 0.202095 | -0.36863 | 1.742782 | 1.987892 | 0.691679 | 5.713216 |
| rs10740322 | -0.87375 | 0.913658 | 0.338909 | -2.66452 | 0.917018 | 0.417382 | 0.069633 | 2.501818 |
| rs10811661 | -0.70511 | 0.34624 | 0.041702 | -1.38374 | -0.02648 | 0.494053 | 0.250639 | 0.973867 |
| rs10842994 | 0.395305 | 0.669154 | 0.554686 | -0.91624 | 1.706846 | 1.484836 | 0.400022 | 5.511549 |
| rs10974438 | 0.237203 | 0.667301 | 0.72224 | -1.07071 | 1.545113 | 1.267698 | 0.342766 | 4.688503 |
| rs11098676 | 1.250819 | 0.921644 | 0.174731 | -0.5556 | 3.057242 | 3.493201 | 0.573725 | 21.26881 |
| rs11107116 | -2.41769 | 0.943518 | 0.010395 | -4.26698 | -0.56839 | 0.089127 | 0.014024 | 0.566436 |
| rs1111875 | 0.153713 | 0.403341 | 0.703129 | -0.63683 | 0.944261 | 1.166156 | 0.528964 | 2.570912 |
| rs11257655 | 0.641341 | 0.591649 | 0.27837 | -0.51829 | 1.800972 | 1.899025 | 0.595538 | 6.055529 |
| rs1127655 | -0.37207 | 0.873349 | 0.670092 | -2.08383 | 1.339698 | 0.689309 | 0.124453 | 3.817892 |
| rs11708067 | 0.343649 | 0.526247 | 0.513744 | -0.68779 | 1.375092 | 1.410083 | 0.502683 | 3.955441 |
| rs11925227 | -1.54109 | 0.902835 | 0.087832 | -3.31065 | 0.228465 | 0.214147 | 0.036492 | 1.25667 |
| rs11926707 | -0.64745 | 0.870743 | 0.457141 | -2.35411 | 1.059205 | 0.523378 | 0.094978 | 2.884077 |
| rs12088739 | -1.26549 | 0.920451 | 0.169177 | -3.06957 | 0.538598 | 0.282102 | 0.046441 | 1.713603 |
| rs12299509 | 0.0489 | 0.8313 | 0.953093 | -1.58045 | 1.678248 | 1.050115 | 0.205883 | 5.356161 |
| rs12617659 | 1.547387 | 0.780658 | 0.047462 | 0.017296 | 3.077477 | 4.699175 | 1.017447 | 21.70358 |
| rs12910825 | -1.53141 | 0.763634 | 0.044918 | -3.02813 | -0.03468 | 0.216231 | 0.048406 | 0.965912 |
| rs12945601 | -1.36025 | 0.800752 | 0.089372 | -2.92973 | 0.20922 | 0.256596 | 0.053412 | 1.232716 |
| rs12970134 | -0.97569 | 0.864061 | 0.258816 | -2.66925 | 0.717868 | 0.376931 | 0.069304 | 2.050058 |
| rs13239186 | 1.101876 | 0.739365 | 0.136146 | -0.34728 | 2.551032 | 3.009806 | 0.706607 | 12.82033 |
| rs13330951 | -0.76057 | 0.839618 | 0.365017 | -2.40622 | 0.885086 | 0.467402 | 0.090156 | 2.423194 |
| rs13389219 | 0.101859 | 0.554857 | 0.854345 | -0.98566 | 1.18938 | 1.107228 | 0.373192 | 3.285043 |
| rs1359790 | 0.214883 | 0.526263 | 0.683039 | -0.81659 | 1.246358 | 1.239717 | 0.441935 | 3.477654 |
| rs1496653 | 0.158489 | 0.538433 | 0.768489 | -0.89684 | 1.213818 | 1.171739 | 0.407857 | 3.366312 |
| rs1552224 | 0.097607 | 0.435094 | 0.822496 | -0.75518 | 0.950391 | 1.10253 | 0.469928 | 2.586721 |
| rs16988333 | -1.3095 | 0.92135 | 0.155234 | -3.11535 | 0.496345 | 0.269954 | 0.044363 | 1.642706 |
| rs17086692 | 0.486769 | 0.880694 | 0.580462 | -1.23939 | 2.212929 | 1.62705 | 0.28956 | 9.142451 |
| rs17168486 | -0.48462 | 0.646036 | 0.453165 | -1.75086 | 0.781607 | 0.615929 | 0.173625 | 2.184981 |
| rs17405722 | -0.16448 | 0.731268 | 0.82204 | -1.59776 | 1.268807 | 0.848336 | 0.202349 | 3.556606 |
| rs17631783 | -0.44817 | 0.935501 | 0.63189 | -2.28175 | 1.385414 | 0.638797 | 0.102105 | 3.996479 |
| rs17791513 | -0.2615 | 0.576252 | 0.649981 | -1.39095 | 0.867958 | 0.769898 | 0.248839 | 2.382041 |
| rs1801214 | -0.21204 | 0.428567 | 0.620768 | -1.05203 | 0.627953 | 0.808933 | 0.349228 | 1.87377 |
| rs1899951 | -0.03308 | 0.453069 | 0.941793 | -0.9211 | 0.854933 | 0.96746 | 0.398082 | 2.351218 |
| rs2237892 | -1.03258 | 0.894046 | 0.24811 | -2.78491 | 0.71975 | 0.356087 | 0.061735 | 2.053919 |
| rs2246618 | 0.983975 | 0.816187 | 0.227981 | -0.61575 | 2.583701 | 2.675068 | 0.540234 | 13.24608 |
| rs2261181 | 0.263774 | 0.746517 | 0.723834 | -1.1994 | 1.726946 | 1.301833 | 0.301375 | 5.623456 |

| | | | | | | | | |
|---|---|---|---|---|---|---|---|---|
| rs2294120 | −1.65454 | 0.866585 | 0.056228 | −3.35305 | 0.043964 | 0.19118 | 0.034978 | 1.044945 |
| rs2296173 | 0.111514 | 0.751955 | 0.882107 | −1.36232 | 1.585346 | 1.117969 | 0.256066 | 4.880982 |
| rs2299383 | 2.148228 | 0.93001 | 0.020894 | 0.325409 | 3.971047 | 8.569661 | 1.384597 | 53.04004 |
| rs243019 | −1.01831 | 0.67814 | 0.133196 | −2.34746 | 0.310848 | 0.361206 | 0.095612 | 1.364582 |
| rs2493394 | 0.241221 | 0.761679 | 0.751475 | −1.25167 | 1.734112 | 1.272802 | 0.286026 | 5.663898 |
| rs2796441 | 0.419354 | 0.540607 | 0.437921 | −0.64024 | 1.478944 | 1.520978 | 0.527168 | 4.388307 |
| rs2820426 | 0.722779 | 0.751131 | 0.335921 | −0.74944 | 2.194995 | 2.060151 | 0.472633 | 8.979957 |
| rs2867125 | 0.833048 | 0.856356 | 0.330661 | −0.84541 | 2.511506 | 2.30032 | 0.429381 | 12.32348 |
| rs2908282 | −2.05614 | 1.124185 | 0.067399 | −4.25954 | 0.147261 | 0.127947 | 0.014129 | 1.158656 |
| rs2925979 | 0.848785 | 0.764719 | 0.267029 | −0.65006 | 2.347634 | 2.336805 | 0.522012 | 10.46079 |
| rs2943656 | 0.02632 | 0.434771 | 0.951728 | −0.82583 | 0.87847 | 1.026669 | 0.437871 | 2.407214 |
| rs3217992 | −0.7105 | 0.742497 | 0.338614 | −2.1658 | 0.744793 | 0.491398 | 0.114659 | 2.106006 |
| rs340874 | 0.761882 | 0.616414 | 0.216462 | −0.44629 | 1.970053 | 2.142304 | 0.639999 | 7.171055 |
| rs348330 | 1.400906 | 0.820246 | 0.087653 | −0.20678 | 3.008589 | 4.058874 | 0.813201 | 20.25878 |
| rs3756784 | 1.115208 | 0.912366 | 0.221585 | −0.67303 | 2.903446 | 3.050202 | 0.51016 | 18.23688 |
| rs3802177 | −0.63614 | 0.32362 | 0.049334 | −1.27044 | −0.00184 | 0.529332 | 0.280709 | 0.998158 |
| rs459193 | 0.126269 | 0.575252 | 0.826258 | −1.00122 | 1.253763 | 1.134588 | 0.367429 | 3.503502 |
| rs4622883 | 1.019324 | 0.904915 | 0.259983 | −0.75431 | 2.792957 | 2.771321 | 0.470335 | 16.32924 |
| rs4686471 | −1.46753 | 0.739275 | 0.047134 | −2.91651 | −0.01855 | 0.230493 | 0.054122 | 0.981617 |
| rs4812829 | −0.95032 | 0.888367 | 0.284736 | −2.69152 | 0.790877 | 0.386617 | 0.067778 | 2.20533 |
| rs4823182 | −1.18734 | 0.796452 | 0.136016 | −2.74839 | 0.373702 | 0.30503 | 0.064031 | 1.453104 |
| rs4865796 | 0.009023 | 0.765464 | 0.990595 | −1.49129 | 1.509333 | 1.009064 | 0.225083 | 4.523711 |
| rs516946 | 0.757149 | 0.574608 | 0.187611 | −0.36908 | 1.883381 | 2.132189 | 0.691368 | 6.5757 |
| rs5215 | 0.054353 | 0.563398 | 0.923144 | −1.04991 | 1.158614 | 1.055857 | 0.34997 | 3.185514 |
| rs576674 | 1.185199 | 0.995668 | 0.233907 | −0.76631 | 3.136708 | 3.271337 | 0.464724 | 23.02794 |
| rs6059662 | 0.367491 | 1.019697 | 0.718554 | −1.63112 | 2.366098 | 1.444107 | 0.195711 | 10.65573 |
| rs61953351 | 0.859581 | 0.606883 | 0.156662 | −0.32991 | 2.049072 | 2.362172 | 0.718989 | 7.760695 |
| rs622217 | −0.33574 | 0.786256 | 0.669375 | −1.8768 | 1.205325 | 0.714812 | 0.15308 | 3.337844 |
| rs6515236 | 0.802175 | 0.931613 | 0.389205 | −1.02379 | 2.628136 | 2.230386 | 0.359232 | 13.84794 |
| rs67232546 | −1.38318 | 0.895938 | 0.122629 | −3.13921 | 0.372862 | 0.250781 | 0.043317 | 1.451884 |
| rs6767484 | 0.208016 | 0.341797 | 0.542793 | −0.46191 | 0.877937 | 1.231233 | 0.630082 | 2.405931 |
| rs6785040 | 0.174401 | 0.728104 | 0.810696 | −1.25268 | 1.601486 | 1.190533 | 0.285737 | 4.960396 |
| rs6795735 | −1.27216 | 0.712611 | 0.074227 | −2.66888 | 0.124558 | 0.280226 | 0.06933 | 1.132648 |
| rs6878122 | −0.74402 | 0.805099 | 0.355418 | −2.32201 | 0.833977 | 0.475201 | 0.098076 | 2.302457 |
| rs6960043 | 0.126175 | 0.607458 | 0.835456 | −1.06444 | 1.316792 | 1.13448 | 0.34492 | 3.731432 |
| rs7144011 | −0.38926 | 0.925548 | 0.674064 | −2.20334 | 1.42481 | 0.677556 | 0.110434 | 4.157068 |
| rs7177055 | −0.60824 | 0.639464 | 0.341515 | −1.86159 | 0.645106 | 0.544307 | 0.155425 | 1.906189 |
| rs7240767 | −0.49161 | 0.86704 | 0.570714 | −2.19101 | 1.207786 | 0.61164 | 0.111804 | 3.346069 |
| rs72892910 | −1.02324 | 0.731488 | 0.16186 | −2.45695 | 0.41048 | 0.35943 | 0.085696 | 1.507541 |
| rs735949 | −0.10309 | 0.887354 | 0.907516 | −1.8423 | 1.636128 | 0.902049 | 0.158452 | 5.135248 |
| rs753270 | 0.168197 | 0.732462 | 0.818378 | −1.26743 | 1.603822 | 1.183169 | 0.281555 | 4.972001 |
| rs7561798 | 0.132821 | 0.95897 | 0.889842 | −1.74676 | 2.012402 | 1.142046 | 0.174338 | 7.481269 |
| rs7572970 | 1.828373 | 0.80071 | 0.022405 | 0.258981 | 3.397765 | 6.223752 | 1.295609 | 29.8972 |
| rs7674212 | 0.580327 | 0.831396 | 0.485168 | −1.04921 | 2.209862 | 1.786622 | 0.350215 | 9.114463 |
| rs7685296 | 0.443841 | 0.823184 | 0.589765 | −1.1696 | 2.057282 | 1.558683 | 0.310491 | 7.824674 |
| rs7729395 | 0.511799 | 0.601479 | 0.394825 | −0.6671 | 1.690698 | 1.66829 | 0.513195 | 5.423266 |
| rs7756992 | −0.19955 | 0.312072 | 0.522534 | −0.81121 | 0.412109 | 0.819097 | 0.444318 | 1.509999 |

| | | | | | | | | |
|---|---|---|---|---|---|---|---|---|
| rs7786095 | 1.241349 | 1.106236 | 0.261804 | -0.92687 | 3.40957 | 3.460277 | 0.395789 | 30.25224 |
| rs780094 | -0.62927 | 0.577938 | 0.276232 | -1.76203 | 0.503487 | 0.53298 | 0.171696 | 1.65448 |
| rs7845219 | -1.08234 | 0.903341 | 0.230856 | -2.85289 | 0.688205 | 0.338801 | 0.057677 | 1.99014 |
| rs7903146 | -0.08797 | 0.155773 | 0.572277 | -0.39328 | 0.21735 | 0.915793 | 0.674839 | 1.242779 |
| rs7929543 | -0.68288 | 0.875031 | 0.435151 | -2.39794 | 1.03218 | 0.505159 | 0.090905 | 2.807179 |
| rs7955901 | -0.08934 | 0.88932 | 0.91998 | -1.83241 | 1.653727 | 0.914535 | 0.160028 | 5.226424 |
| rs8068804 | -0.69763 | 0.710295 | 0.326014 | -2.08981 | 0.694544 | 0.497762 | 0.12371 | 2.002795 |
| rs8108269 | 1.029601 | 0.639398 | 0.10734 | -0.22362 | 2.28282 | 2.799948 | 0.79962 | 9.80429 |
| rs825476 | 0.025453 | 0.743244 | 0.972681 | -1.43131 | 1.482212 | 1.02578 | 0.238997 | 4.402674 |
| rs840967 | 1.842425 | 0.77198 | 0.017004 | 0.329344 | 3.355505 | 6.311823 | 1.390056 | 28.66008 |
| rs849135 | 0.773979 | 0.383473 | 0.043556 | 0.022371 | 1.525587 | 2.168377 | 1.022623 | 4.597842 |
| rs853974 | -0.02951 | 0.715226 | 0.967087 | -1.43136 | 1.372332 | 0.970919 | 0.238985 | 3.944538 |
| rs9369425 | 0.857846 | 0.756196 | 0.256617 | -0.6243 | 2.33999 | 2.358076 | 0.535637 | 10.38114 |
| rs9894220 | -0.22111 | 0.662656 | 0.738628 | -1.51992 | 1.077697 | 0.801629 | 0.21873 | 2.937906 |
| rs9928094 | -0.01963 | 0.36835 | 0.957496 | -0.7416 | 0.702335 | 0.98056 | 0.476352 | 2.01846 |
| rs993380 | 1.20612 | 0.768976 | 0.11677 | -0.30107 | 2.713314 | 3.340499 | 0.740024 | 15.07916 |
| rs9940149 | 0.601383 | 1.065979 | 0.572645 | -1.48794 | 2.690702 | 1.82464 | 0.225838 | 14.74202 |
| All - Inverse variance weighted | -0.03004 | 0.066924 | 0.653507 | -0.16121 | 0.101129 | 0.970405 | 0.851111 | 1.106419 |
| All - MR Egger | -0.03762 | 0.159153 | 0.813583 | -0.34956 | 0.274317 | 0.963075 | 0.704995 | 1.315631 |

**Table 2:** ebi-a-GCST005413

| SNP | b | se | p | lo_ci | up_ci | or | or_lci95 | or_uci95 |
|---|---|---|---|---|---|---|---|---|
| rs10404333 | 0.285091 | 0.415555 | 0.492682 | -0.5294 | 1.09958 | 1.329883 | 0.58896 | 3.002904 |
| rs10771367 | 0.153233 | 0.510071 | 0.76386 | -0.84651 | 1.152971 | 1.165597 | 0.428911 | 3.167591 |
| rs10811662 | -0.68836 | 0.34128 | 0.043697 | -1.35727 | -0.01945 | 0.502401 | 0.257363 | 0.98074 |
| rs10820725 | -0.06381 | 0.497889 | 0.898019 | -1.03967 | 0.912051 | 0.938182 | 0.35357 | 2.489424 |
| rs10849427 | 0.034971 | 0.463405 | 0.939844 | -0.8733 | 0.943245 | 1.03559 | 0.41757 | 2.568302 |
| rs111268402 | -0.52864 | 0.298356 | 0.07642 | -1.11342 | 0.056137 | 0.589405 | 0.328434 | 1.057742 |
| rs112538588 | 0.183778 | 0.378598 | 0.62738 | -0.55827 | 0.92583 | 1.201749 | 0.572196 | 2.523962 |
| rs11257655 | 0.392581 | 0.362164 | 0.27837 | -0.31726 | 1.102422 | 1.480798 | 0.728142 | 3.011451 |
| rs114584210 | 0.350777 | 0.4575 | 0.443245 | -0.54592 | 1.247477 | 1.420171 | 0.579307 | 3.481548 |
| rs11616380 | 0.169754 | 0.449674 | 0.705799 | -0.71161 | 1.051115 | 1.185013 | 0.490854 | 2.86084 |
| rs11708067 | 0.370941 | 0.56804 | 0.513744 | -0.74242 | 1.4843 | 1.449097 | 0.475962 | 4.411874 |
| rs13266634 | -0.58724 | 0.285818 | 0.039919 | -1.14744 | -0.02704 | 0.55586 | 0.317448 | 0.973327 |
| rs13296301 | 0.155899 | 0.479354 | 0.745008 | -0.78363 | 1.095433 | 1.168708 | 0.456743 | 2.990476 |
| rs139703826 | -0.05863 | 0.40455 | 0.884775 | -0.85154 | 0.73429 | 0.943059 | 0.426755 | 2.084002 |
| rs1538742 | 0.585534 | 0.4837 | 0.226076 | -0.36252 | 1.533586 | 1.795949 | 0.695921 | 4.634768 |
| rs1563575 | 1.090781 | 0.488445 | 0.025538 | 0.133429 | 2.048133 | 2.976599 | 1.142741 | 7.753412 |
| rs1708302 | 0.548976 | 0.273408 | 0.044654 | 0.013096 | 1.084856 | 1.731479 | 1.013182 | 2.959014 |
| rs2057859 | -0.25402 | 0.601685 | 0.672895 | -1.43332 | 0.925284 | 0.775678 | 0.238516 | 2.522585 |
| rs2229429 | -0.1618 | 0.62278 | 0.795017 | -1.38245 | 1.058849 | 0.850612 | 0.250964 | 2.883052 |
| rs2821353 | 0.454577 | 0.593828 | 0.443972 | -0.70933 | 1.61848 | 1.575507 | 0.491976 | 5.045416 |
| rs2925979 | 0.52399 | 0.472092 | 0.267029 | -0.40131 | 1.449291 | 1.688752 | 0.669441 | 4.260092 |
| rs2943656 | 0.02296 | 0.379268 | 0.951728 | -0.72041 | 0.766325 | 1.023225 | 0.486555 | 2.151844 |
| rs340874 | 0.566435 | 0.458284 | 0.216462 | -0.3318 | 1.464671 | 1.761974 | 0.71763 | 4.32612 |
| rs34872471 | -0.06539 | 0.134763 | 0.62754 | -0.32952 | 0.19875 | 0.936706 | 0.719267 | 1.219877 |

| | | | | | | | | |
|---|---|---|---|---|---|---|---|---|
| rs35261542 | -0.20656 | 0.304943 | 0.49817 | -0.80425 | 0.391128 | 0.813378 | 0.447424 | 1.478648 |
| rs3768321 | 0.048207 | 0.462353 | 0.91696 | -0.85801 | 0.954419 | 1.049388 | 0.424007 | 2.597162 |
| rs3792711 | -0.1122 | 0.494938 | 0.820657 | -1.08228 | 0.857877 | 0.893863 | 0.338821 | 2.358149 |
| rs3843467 | 0.796974 | 0.438509 | 0.069146 | -0.0625 | 1.656451 | 2.218816 | 0.93941 | 5.240677 |
| rs4234733 | -0.417 | 0.442637 | 0.346153 | -1.28457 | 0.45057 | 0.659022 | 0.276771 | 1.569206 |
| rs4367411 | -0.05565 | 0.47064 | 0.905871 | -0.97811 | 0.866803 | 0.945868 | 0.376022 | 2.379291 |
| rs4607103 | -0.38543 | 0.458034 | 0.400069 | -1.28318 | 0.512312 | 0.680155 | 0.277154 | 1.669146 |
| rs4746890 | -0.37425 | 0.42743 | 0.381259 | -1.21201 | 0.463514 | 0.687806 | 0.297598 | 1.589649 |
| rs6121871 | 0.397179 | 0.294656 | 0.177677 | -0.18035 | 0.974704 | 1.487622 | 0.834981 | 2.650383 |
| rs66502159 | 0.113399 | 0.465903 | 0.807698 | -0.79977 | 1.02657 | 1.120079 | 0.449432 | 2.791474 |
| rs6743071 | 0.364637 | 0.518706 | 0.482072 | -0.65203 | 1.381301 | 1.439992 | 0.520989 | 3.980078 |
| rs703983 | -0.13 | 0.311475 | 0.67641 | -0.74049 | 0.480492 | 0.878096 | 0.47688 | 1.616869 |
| rs71304101 | -0.0295 | 0.251822 | 0.906752 | -0.52307 | 0.464073 | 0.970933 | 0.592699 | 1.590539 |
| rs71320321 | 0.184744 | 0.303861 | 0.543195 | -0.41082 | 0.780312 | 1.202911 | 0.663104 | 2.182152 |
| rs7153662 | -0.44124 | 0.457592 | 0.334913 | -1.33812 | 0.455641 | 0.643238 | 0.262338 | 1.577184 |
| rs74850119 | 0.045351 | 0.656416 | 0.944919 | -1.24122 | 1.331926 | 1.046395 | 0.28903 | 3.788332 |
| rs7522969 | 0.526063 | 0.473167 | 0.266228 | -0.40134 | 1.453471 | 1.692257 | 0.669419 | 4.277938 |
| rs75233777 | 0.78899 | 0.425631 | 0.063783 | -0.04525 | 1.623227 | 2.201173 | 0.955762 | 5.069422 |
| rs752579 | -0.69113 | 0.484704 | 0.153903 | -1.64115 | 0.25889 | 0.50101 | 0.193757 | 1.295492 |
| rs75432112 | -0.07571 | 0.496998 | 0.878923 | -1.04983 | 0.898406 | 0.927085 | 0.349999 | 2.455685 |
| rs757110 | 0.099905 | 0.43852 | 0.819783 | -0.75959 | 0.959404 | 1.105066 | 0.467856 | 2.610142 |
| rs7589501 | -1.2851 | 0.53186 | 0.015682 | -2.32755 | -0.24265 | 0.276623 | 0.097535 | 0.784543 |
| rs76895963 | 0.093609 | 0.21089 | 0.657132 | -0.31974 | 0.506954 | 1.09813 | 0.726341 | 1.660226 |
| rs7903302 | 0.025198 | 0.403201 | 0.950169 | -0.76508 | 0.815473 | 1.025518 | 0.465298 | 2.260244 |
| rs79430446 | 0.102727 | 0.442664 | 0.816488 | -0.76489 | 0.970348 | 1.108188 | 0.465383 | 2.638862 |
| rs7977788 | -1.09918 | 0.516686 | 0.03339 | -2.11188 | -0.08647 | 0.333145 | 0.12101 | 0.91716 |
| rs79966456 | 0.398953 | 0.489179 | 0.414754 | -0.55984 | 1.357743 | 1.490263 | 0.571302 | 3.887408 |
| rs9268835 | -0.11974 | 0.356683 | 0.737103 | -0.81884 | 0.579364 | 0.887155 | 0.440945 | 1.784902 |
| rs9634624 | -0.52986 | 0.541663 | 0.327973 | -1.59152 | 0.531801 | 0.588689 | 0.203617 | 1.701995 |
| rs999474 | -0.17628 | 0.526715 | 0.737862 | -1.20864 | 0.856077 | 0.83838 | 0.298602 | 2.353908 |
| All - Inverse variance weighted | 0.010539 | 0.053689 | 0.844378 | -0.09469 | 0.115768 | 1.010595 | 0.909654 | 1.122736 |
| All - MR Egger | 0.006305 | 0.123197 | 0.959381 | -0.23516 | 0.247771 | 1.006325 | 0.790443 | 1.281166 |

**Table 3:** GCST90006934

| SNP | b | se | p | lo_ci | up_ci | or | or_lci95 | or_uci95 |
|---|---|---|---|---|---|---|---|---|
| rs10811660 | -0.79276 | 0.388883 | 0.041493 | -1.55497 | -0.03055 | 0.452593 | 0.211195 | 0.969909 |
| rs113617270 | 0.322344 | 0.35847 | 0.368535 | -0.38026 | 1.024945 | 1.380359 | 0.683685 | 2.786941 |
| rs113897089 | 0.255591 | 0.60357 | 0.671956 | -0.92741 | 1.438588 | 1.291225 | 0.395578 | 4.214742 |
| rs11603349 | 0.064399 | 0.336361 | 0.848165 | -0.59487 | 0.723666 | 1.066518 | 0.551636 | 2.061979 |
| rs12121080 | 0.48996 | 0.444985 | 0.270866 | -0.38221 | 1.36213 | 1.632251 | 0.682352 | 3.9045 |
| rs138772829 | -0.47247 | 0.459946 | 0.304307 | -1.37397 | 0.429018 | 0.623457 | 0.253101 | 1.535749 |
| rs1391265 | -0.13426 | 0.437986 | 0.759195 | -0.99271 | 0.724194 | 0.874363 | 0.37057 | 2.063067 |
| rs140367104 | -0.04378 | 0.760282 | 0.954084 | -1.53393 | 1.446378 | 0.957168 | 0.215686 | 4.2477 |
| rs143017296 | 0.971768 | 0.580997 | 0.094409 | -0.16699 | 2.110522 | 2.642612 | 0.846211 | 8.252551 |
| rs143454009 | 1.087123 | 0.708599 | 0.124984 | -0.30173 | 2.475977 | 2.965729 | 0.739536 | 11.89332 |
| rs146580015 | -0.1815 | 0.248862 | 0.465816 | -0.66927 | 0.306274 | 0.834022 | 0.512085 | 1.358354 |

| | | | | | | | | |
|---|---|---|---|---|---|---|---|---|
| rs148259165 | −0.04595 | 0.166745 | 0.782896 | −0.37277 | 0.280874 | 0.955093 | 0.688826 | 1.324286 |
| rs149736582 | −0.58395 | 0.329321 | 0.076197 | −1.22942 | 0.061519 | 0.557691 | 0.292463 | 1.063451 |
| rs1549182 | 0.089404 | 0.395176 | 0.821017 | −0.68514 | 0.863949 | 1.093522 | 0.504019 | 2.37251 |
| rs17154959 | 0.018601 | 0.421585 | 0.964807 | −0.8077 | 0.844907 | 1.018775 | 0.44588 | 2.327761 |
| rs1977833 | 0.11681 | 0.290143 | 0.687248 | −0.45187 | 0.685491 | 1.123905 | 0.636436 | 1.984745 |
| rs2237895 | 0.687821 | 0.414411 | 0.096964 | −0.12442 | 1.500067 | 1.989377 | 0.883005 | 4.48199 |
| rs34356122 | 0.819526 | 0.462459 | 0.076377 | −0.08689 | 1.725945 | 2.269424 | 0.916775 | 5.617827 |
| rs3768321 | 0.047654 | 0.457053 | 0.91696 | −0.84817 | 0.943478 | 1.048808 | 0.428198 | 2.5689 |
| rs4130874 | 0.003837 | 0.426946 | 0.992829 | −0.83298 | 0.840651 | 1.003845 | 0.434753 | 2.317875 |
| rs4300038 | −0.55939 | 0.294193 | 0.057243 | −1.13601 | 0.017224 | 0.571555 | 0.321097 | 1.017373 |
| rs4929965 | 0.458748 | 0.438332 | 0.295295 | −0.40038 | 1.317878 | 1.582092 | 0.670064 | 3.735485 |
| rs4932262 | 0.083939 | 0.449587 | 0.851893 | −0.79725 | 0.965131 | 1.087563 | 0.450565 | 2.625131 |
| rs552719 | 0.751361 | 0.433414 | 0.082991 | −0.09813 | 1.600852 | 2.119884 | 0.906532 | 4.957254 |
| rs55721115 | −0.19657 | 0.347654 | 0.57178 | −0.87798 | 0.484827 | 0.82154 | 0.415623 | 1.623895 |
| rs61953351 | 0.587604 | 0.414861 | 0.156662 | −0.22552 | 1.400733 | 1.799672 | 0.798098 | 4.058172 |
| rs62175628 | −0.21888 | 0.361838 | 0.545244 | −0.92808 | 0.490325 | 0.803421 | 0.395313 | 1.632847 |
| rs62501410 | −0.2203 | 0.426909 | 0.605837 | −1.05704 | 0.616446 | 0.802282 | 0.347484 | 1.852333 |
| rs713311 | −0.08119 | 0.306074 | 0.790804 | −0.6811 | 0.518713 | 0.922017 | 0.506062 | 1.679865 |
| rs72999033 | 0.110489 | 0.411341 | 0.788231 | −0.69574 | 0.916718 | 1.116825 | 0.498706 | 2.501067 |
| rs73021345 | −0.10186 | 0.438734 | 0.816405 | −0.96178 | 0.758057 | 0.903155 | 0.382212 | 2.134126 |
| rs7304270 | −0.23474 | 0.397767 | 0.555098 | −1.01436 | 0.544886 | 0.790779 | 0.362635 | 1.724411 |
| rs73122161 | −0.33829 | 0.449436 | 0.451636 | −1.21918 | 0.542609 | 0.712991 | 0.295472 | 1.720489 |
| rs7546395 | −0.27797 | 0.367917 | 0.449929 | −0.99909 | 0.443143 | 0.757317 | 0.368214 | 1.557595 |
| rs7756992 | −0.18188 | 0.28444 | 0.522534 | −0.73939 | 0.375619 | 0.833699 | 0.477407 | 1.455892 |
| rs7903146 | −0.09586 | 0.169758 | 0.572277 | −0.42859 | 0.236863 | 0.908589 | 0.651428 | 1.267267 |
| rs7920427 | −0.03593 | 0.469484 | 0.938999 | −0.95612 | 0.88426 | 0.964709 | 0.384382 | 2.421193 |
| rs8056223 | −0.02947 | 0.322101 | 0.927093 | −0.66079 | 0.601845 | 0.970957 | 0.516443 | 1.825484 |
| rs952227 | −0.12956 | 0.362233 | 0.720588 | −0.83954 | 0.580415 | 0.878481 | 0.431911 | 1.786781 |
| rs9972653 | −0.17109 | 0.359074 | 0.633743 | −0.87487 | 0.532699 | 0.842749 | 0.416916 | 1.703524 |
| All - Inverse variance weighted | −0.03636 | 0.055474 | 0.512227 | −0.14508 | 0.072372 | 0.964297 | 0.864949 | 1.075056 |
| All - MR Egger | −0.18654 | 0.122096 | 0.134845 | −0.42585 | 0.05277 | 0.829827 | 0.653217 | 1.054188 |

**Table 4:** GCST90026414

| SNP | b | se | p | lo_ci | up_ci | or | or_lci95 | or_uci95 |
|---|---|---|---|---|---|---|---|---|
| rs11649532 | −0.11629 | 0.121064 | 0.336773 | −0.35358 | 0.120996 | 0.890217 | 0.702173 | 1.128621 |
| rs12186187 | 0.098459 | 0.122811 | 0.42272 | −0.14225 | 0.339169 | 1.103469 | 0.867404 | 1.40378 |
| rs12356932 | −0.18998 | 0.161959 | 0.240791 | −0.50742 | 0.127459 | 0.826976 | 0.602048 | 1.135939 |
| rs12928 | 0.020015 | 0.141141 | 0.887233 | −0.25662 | 0.296652 | 1.020216 | 0.77366 | 1.345347 |
| rs17433836 | −0.16053 | 0.147823 | 0.277487 | −0.45027 | 0.1292 | 0.85169 | 0.637458 | 1.137918 |
| rs509761 | −0.04594 | 0.127192 | 0.717958 | −0.29524 | 0.203355 | 0.955099 | 0.744356 | 1.225508 |
| rs61876364 | 0.111205 | 0.164667 | 0.499464 | −0.21154 | 0.433954 | 1.117624 | 0.809335 | 1.543347 |
| rs7177676 | 0.102471 | 0.140315 | 0.46521 | −0.17255 | 0.377489 | 1.107906 | 0.84152 | 1.458617 |
| rs77320512 | −0.17138 | 0.159505 | 0.282623 | −0.48401 | 0.14125 | 0.842502 | 0.616308 | 1.151713 |
| rs79411961 | −0.35593 | 0.195655 | 0.068885 | −0.73941 | 0.027553 | 0.700522 | 0.477394 | 1.027937 |
| rs80346529 | 0.05593 | 0.110604 | 0.613083 | −0.16085 | 0.272715 | 1.057524 | 0.851416 | 1.313526 |
| All - Inverse variance | −0.03536 | 0.04206 | 0.400547 | −0.1178 | 0.04708 | 0.96526 | 0.888878 | 1.048206 |

| | | | | | | | | |
|---|---|---|---|---|---|---|---|---|
| weighted | | | | | | | | |
| All - MR Egger | -0.2543 | 0.17517 | 0.180526 | -0.59763 | 0.089033 | 0.775459 | 0.550111 | 1.093117 |

**Table 5:** GCST90026417

| SNP | b | se | p | lo_ci | up_ci | or | or_lci95 | or_uci95 |
|---|---|---|---|---|---|---|---|---|
| rs11755896 | -0.09414 | 0.305861 | 0.75825 | -0.69363 | 0.50535 | 0.910157 | 0.499761 | 1.657566 |
| rs117658639 | -0.20327 | 0.18693 | 0.27685 | -0.56965 | 0.163111 | 0.816056 | 0.565721 | 1.177167 |
| rs1176785 | 0.138006 | 0.232125 | 0.552157 | -0.31696 | 0.592971 | 1.147982 | 0.72836 | 1.809356 |
| rs12328416 | -0.17889 | 0.264574 | 0.498938 | -0.69746 | 0.33967 | 0.836194 | 0.497849 | 1.404484 |
| rs12347620 | -0.16226 | 0.260021 | 0.532606 | -0.6719 | 0.347379 | 0.850219 | 0.510736 | 1.415353 |
| rs17092429 | 0.046442 | 0.280321 | 0.868413 | -0.50299 | 0.595872 | 1.047537 | 0.604721 | 1.814612 |
| rs191858852 | -0.26196 | 0.263842 | 0.320769 | -0.77909 | 0.255167 | 0.769539 | 0.458822 | 1.290678 |
| rs1959005 | -0.08833 | 0.198517 | 0.656346 | -0.47743 | 0.30076 | 0.915456 | 0.620378 | 1.350886 |
| rs2001625 | -0.13945 | 0.209447 | 0.505524 | -0.54997 | 0.271061 | 0.869833 | 0.576967 | 1.311355 |
| rs2243909 | 0.098059 | 0.21657 | 0.650705 | -0.32642 | 0.522535 | 1.103028 | 0.721504 | 1.686297 |
| rs28366124 | -0.18693 | 0.238914 | 0.433964 | -0.6552 | 0.281339 | 0.829499 | 0.519336 | 1.324903 |
| rs5848 | -0.21285 | 0.236874 | 0.368869 | -0.67713 | 0.25142 | 0.808275 | 0.508075 | 1.285849 |
| rs61815651 | -0.18078 | 0.332318 | 0.586433 | -0.83213 | 0.470558 | 0.834615 | 0.435123 | 1.600887 |
| rs7519349 | 0.106061 | 0.265474 | 0.689514 | -0.41427 | 0.62639 | 1.11189 | 0.660824 | 1.870845 |
| rs754733 | 0.137097 | 0.235074 | 0.559754 | -0.32365 | 0.597842 | 1.146939 | 0.723505 | 1.818191 |
| rs7646518 | 0.155236 | 0.256116 | 0.544439 | -0.34675 | 0.657224 | 1.167933 | 0.70698 | 1.929429 |
| rs7903146 | -0.0908 | 0.160802 | 0.572277 | -0.40598 | 0.224366 | 0.913196 | 0.666326 | 1.251529 |
| rs8070203 | -0.16519 | 0.289559 | 0.568335 | -0.73273 | 0.40234 | 0.847729 | 0.480595 | 1.49532 |
| rs9312914 | 0.095205 | 0.245798 | 0.698512 | -0.38656 | 0.576969 | 1.099884 | 0.67939 | 1.780633 |
| rs9568809 | -0.09732 | 0.253756 | 0.701348 | -0.59468 | 0.400046 | 0.907269 | 0.55174 | 1.491893 |
| All - Inverse variance weighted | -0.06559 | 0.052842 | 0.214506 | -0.16916 | 0.037979 | 0.936514 | 0.844373 | 1.038709 |
| All - MR Egger | -0.29233 | 0.194409 | 0.15001 | -0.67337 | 0.088715 | 0.746524 | 0.509987 | 1.092769 |

**Table 6:** GCST90043636

| SNP | b | se | p | lo_ci | up_ci | or | or_lci95 | or_uci95 |
|---|---|---|---|---|---|---|---|---|
| rs10240601 | -0.00014 | 0.040887 | 0.997338 | -0.08027 | 0.080002 | 0.999864 | 0.922863 | 1.083289 |
| rs115136539 | 0.029257 | 0.039847 | 0.462803 | -0.04884 | 0.107358 | 1.02969 | 0.952331 | 1.113332 |
| rs140830647 | -0.00229 | 0.033309 | 0.945197 | -0.06758 | 0.062997 | 0.997713 | 0.934657 | 1.065023 |
| rs144224508 | 0.035234 | 0.044754 | 0.431114 | -0.05248 | 0.122951 | 1.035862 | 0.94887 | 1.130829 |
| rs150819700 | -0.01024 | 0.021216 | 0.629416 | -0.05182 | 0.031345 | 0.989815 | 0.9495 | 1.031841 |
| rs524612 | -0.00709 | 0.053034 | 0.893647 | -0.11104 | 0.096856 | 0.992935 | 0.894906 | 1.101702 |
| rs62620048 | 0.03052 | 0.050111 | 0.542493 | -0.0677 | 0.128737 | 1.03099 | 0.934544 | 1.13739 |
| rs76512104 | -0.03823 | 0.05482 | 0.485554 | -0.14568 | 0.069215 | 0.962491 | 0.864437 | 1.071667 |
| rs9271365 | 0.009817 | 0.044525 | 0.825497 | -0.07745 | 0.097087 | 1.009865 | 0.925471 | 1.101956 |
| All - Inverse variance weighted | 0.002017 | 0.01246 | 0.871412 | -0.0224 | 0.026438 | 1.002019 | 0.977844 | 1.026791 |
| All - MR Egger | 0.003955 | 0.019144 | 0.842194 | -0.03357 | 0.041477 | 1.003963 | 0.966991 | 1.042349 |

**Table 7:** ebi-a-GCST005536

| SNP | b | se | p | lo_ci | up_ci | or | or_lci95 | or_uci95 |
|---|---|---|---|---|---|---|---|---|
| rs1018942 | 0.294658 | 0.505002 | 0.559571 | -0.69515 | 1.284462 | 1.342667 | 0.499002 | 3.612724 |

| | | | | | | | | |
|---|---|---|---|---|---|---|---|---|
| rs1052553 | 0.125308 | 0.59084 | 0.83204 | -1.03274 | 1.283354 | 1.133498 | 0.356031 | 3.608723 |
| rs11203203 | -0.17509 | 0.282987 | 0.5361 | -0.72975 | 0.379565 | 0.839381 | 0.482032 | 1.461648 |
| rs113010081 | 0.378299 | 0.349253 | 0.278735 | -0.30624 | 1.062835 | 1.459799 | 0.736212 | 2.894566 |
| rs12150079 | -0.42641 | 0.355503 | 0.230348 | -1.1232 | 0.270373 | 0.652847 | 0.325238 | 1.310454 |
| rs12416116 | 0.113336 | 0.269892 | 0.674537 | -0.41565 | 0.642324 | 1.120008 | 0.659909 | 1.900894 |
| rs12418638 | -0.26415 | 0.42528 | 0.534519 | -1.0977 | 0.569397 | 0.767858 | 0.333638 | 1.767202 |
| rs12927355 | 0.016566 | 0.216624 | 0.939043 | -0.40802 | 0.441148 | 1.016704 | 0.664968 | 1.554491 |
| rs12932357 | 0.018381 | 0.374554 | 0.96086 | -0.71575 | 0.752507 | 1.018551 | 0.488827 | 2.122315 |
| rs1296023 | 0.067028 | 0.432631 | 0.876875 | -0.78093 | 0.914984 | 1.069326 | 0.457981 | 2.496736 |
| rs13415583 | -0.6368 | 0.407388 | 0.118023 | -1.43528 | 0.161682 | 0.528983 | 0.238049 | 1.175486 |
| rs1456988 | 0.065211 | 0.352404 | 0.853192 | -0.6255 | 0.755923 | 1.067385 | 0.534994 | 2.129576 |
| rs151233 | 0.555121 | 0.339534 | 0.10206 | -0.11037 | 1.220607 | 1.742151 | 0.895507 | 3.389245 |
| rs1534422 | -0.08864 | 0.46016 | 0.847251 | -0.99055 | 0.813274 | 0.915176 | 0.371371 | 2.25528 |
| rs1574285 | 0.070676 | 0.333745 | 0.832288 | -0.58346 | 0.724817 | 1.073234 | 0.557962 | 2.064353 |
| rs1701704 | -0.13161 | 0.183228 | 0.472578 | -0.49074 | 0.227516 | 0.876682 | 0.612174 | 1.255478 |
| rs1893217 | -0.08279 | 0.272928 | 0.761644 | -0.61773 | 0.452154 | 0.920549 | 0.53917 | 1.571694 |
| rs2111485 | 0.647961 | 0.234111 | 0.005644 | 0.189103 | 1.106819 | 1.911639 | 1.208165 | 3.02472 |
| rs2168587 | 0.060165 | 0.424951 | 0.88741 | -0.77274 | 0.893069 | 1.062012 | 0.461747 | 2.442615 |
| rs2181527 | -0.22339 | 0.511854 | 0.662527 | -1.22662 | 0.779847 | 0.799805 | 0.293282 | 2.181139 |
| rs2194225 | 0.750847 | 0.379276 | 0.047739 | 0.007465 | 1.494228 | 2.118793 | 1.007493 | 4.455896 |
| rs2269241 | -0.73371 | 0.358676 | 0.040796 | -1.43671 | -0.0307 | 0.480126 | 0.237708 | 0.969764 |
| rs229533 | -0.36679 | 0.363816 | 0.313369 | -1.07987 | 0.346287 | 0.692954 | 0.33964 | 1.413809 |
| rs2304256 | 0.720028 | 0.315703 | 0.022565 | 0.101251 | 1.338806 | 2.054492 | 1.106554 | 3.814487 |
| rs2611215 | -0.00715 | 0.266178 | 0.978562 | -0.52886 | 0.514557 | 0.992873 | 0.589275 | 1.672897 |
| rs2641348 | 0.144355 | 0.372725 | 0.698536 | -0.58619 | 0.874896 | 1.155295 | 0.556446 | 2.398626 |
| rs3024505 | -0.15639 | 0.359617 | 0.663651 | -0.86124 | 0.54846 | 0.855226 | 0.422638 | 1.730587 |
| rs3087243 | 0.160739 | 0.227919 | 0.480656 | -0.28598 | 0.607461 | 1.174379 | 0.751276 | 1.835764 |
| rs3184504 | -0.14308 | 0.145902 | 0.326751 | -0.42905 | 0.142885 | 0.866682 | 0.651127 | 1.153597 |
| rs34185821 | -1.07363 | 0.465405 | 0.021062 | -1.98582 | -0.16144 | 0.341766 | 0.137268 | 0.85092 |
| rs34593439 | 0.116973 | 0.232188 | 0.614412 | -0.33812 | 0.572061 | 1.124089 | 0.713113 | 1.771915 |
| rs3802604 | 0.442067 | 0.429643 | 0.303519 | -0.40003 | 1.284168 | 1.55592 | 0.670297 | 3.611661 |
| rs3842727 | -0.01703 | 0.064867 | 0.792946 | -0.14417 | 0.110113 | 0.983117 | 0.865744 | 1.116404 |
| rs402072 | -0.54198 | 0.386759 | 0.161111 | -1.30003 | 0.216065 | 0.581594 | 0.272523 | 1.241183 |
| rs41295121 | 0.212555 | 0.646999 | 0.742515 | -1.05556 | 1.480674 | 1.236834 | 0.347996 | 4.395907 |
| rs4820830 | -0.03635 | 0.303679 | 0.904724 | -0.63156 | 0.558862 | 0.964303 | 0.531761 | 1.748682 |
| rs4849135 | 0.181381 | 0.4118 | 0.659605 | -0.62575 | 0.98851 | 1.198872 | 0.534861 | 2.687227 |
| rs4930034 | -0.06397 | 0.420482 | 0.879083 | -0.88811 | 0.760175 | 0.938034 | 0.411431 | 2.138651 |
| rs4954573 | 0.855233 | 0.446317 | 0.05534 | -0.01955 | 1.730015 | 2.351922 | 0.98064 | 5.640739 |
| rs4957135 | 0.453257 | 0.438977 | 0.301823 | -0.40714 | 1.313651 | 1.573429 | 0.665553 | 3.719731 |
| rs516246 | 0.096706 | 0.275529 | 0.725602 | -0.44333 | 0.636743 | 1.101536 | 0.641894 | 1.890315 |
| rs56994090 | -0.23319 | 0.297468 | 0.433093 | -0.81623 | 0.34985 | 0.792004 | 0.442097 | 1.418854 |
| rs6043409 | -0.17225 | 0.329637 | 0.601289 | -0.81834 | 0.473838 | 0.841768 | 0.441163 | 1.606146 |
| rs61839660 | -0.26155 | 0.203529 | 0.19877 | -0.66047 | 0.137369 | 0.769859 | 0.516611 | 1.147252 |
| rs62447205 | 0.144059 | 0.349206 | 0.67995 | -0.54039 | 0.828503 | 1.154952 | 0.582523 | 2.289888 |
| rs6592645 | -0.49863 | 0.447632 | 0.265307 | -1.37599 | 0.378726 | 0.60736 | 0.252589 | 1.460423 |
| rs6679677 | -0.14929 | 0.084786 | 0.078273 | -0.31547 | 0.01689 | 0.861319 | 0.729446 | 1.017033 |
| rs6691977 | -0.26997 | 0.323541 | 0.40405 | -0.90411 | 0.364174 | 0.763406 | 0.404904 | 1.439325 |

| | | | | | | | | |
|---|---|---|---|---|---|---|---|---|
| rs6827756 | 0.160263 | 0.291764 | 0.582807 | -0.41159 | 0.732119 | 1.173819 | 0.662593 | 2.079483 |
| rs6840119 | -0.20567 | 0.430847 | 0.633097 | -1.05014 | 0.638786 | 0.814098 | 0.34989 | 1.89418 |
| rs694739 | 0.318666 | 0.455127 | 0.483821 | -0.57338 | 1.210715 | 1.375292 | 0.563616 | 3.355884 |
| rs7239671 | 0.281087 | 0.317683 | 0.376262 | -0.34157 | 0.903747 | 1.324569 | 0.710652 | 2.468836 |
| rs72727394 | -0.30417 | 0.342812 | 0.374925 | -0.97608 | 0.36774 | 0.737734 | 0.376783 | 1.444466 |
| rs72928038 | 0.179457 | 0.331935 | 0.588757 | -0.47114 | 0.830049 | 1.196567 | 0.624293 | 2.293431 |
| rs7795074 | 0.13027 | 0.486035 | 0.78868 | -0.82236 | 1.082898 | 1.139135 | 0.439394 | 2.953224 |
| rs78037977 | 0.321835 | 0.486524 | 0.508293 | -0.63175 | 1.275422 | 1.379657 | 0.531659 | 3.580212 |
| rs7805116 | -0.2799 | 0.463437 | 0.545865 | -1.18824 | 0.628436 | 0.755859 | 0.304758 | 1.874676 |
| rs7839768 | -0.15698 | 0.421196 | 0.709372 | -0.98252 | 0.668564 | 0.854721 | 0.374365 | 1.951432 |
| rs7988301 | 0.537802 | 0.424207 | 0.204876 | -0.29364 | 1.369249 | 1.71224 | 0.745542 | 3.932395 |
| rs8056814 | -0.08779 | 0.249524 | 0.724978 | -0.57685 | 0.401281 | 0.915957 | 0.561663 | 1.493737 |
| rs868093 | -0.20538 | 0.488092 | 0.67392 | -1.16204 | 0.751284 | 0.81434 | 0.312848 | 2.11972 |
| rs911263 | 0.480838 | 0.434871 | 0.268855 | -0.37151 | 1.333186 | 1.61743 | 0.689693 | 3.793108 |
| rs9585056 | 0.881814 | 0.380406 | 0.020445 | 0.136219 | 1.62741 | 2.415278 | 1.145933 | 5.090672 |
| All - Inverse variance weighted | -0.01383 | 0.033762 | 0.681991 | -0.08001 | 0.052339 | 0.986262 | 0.92311 | 1.053733 |
| All - MR Egger | -0.07988 | 0.059315 | 0.18305 | -0.19614 | 0.036376 | 0.923226 | 0.821899 | 1.037046 |

**Table 8:** ebi-a-GCST010681

| SNP | b | se | p | lo_ci | up_ci | or | or_lci95 | or_uci95 |
|---|---|---|---|---|---|---|---|---|
| rs10012242 | -0.58455 | 0.312568 | 0.061463 | -1.19718 | 0.028083 | 0.557357 | 0.302044 | 1.028481 |
| rs10091521 | 0.047589 | 0.216349 | 0.8259 | -0.37645 | 0.471632 | 1.048739 | 0.68629 | 1.602608 |
| rs10183097 | -0.06031 | 0.258648 | 0.815612 | -0.56726 | 0.446635 | 0.941468 | 0.567075 | 1.563044 |
| rs1027769 | -0.17191 | 0.126961 | 0.175728 | -0.42075 | 0.076935 | 0.842056 | 0.656552 | 1.079972 |
| rs1049371 | -0.29175 | 0.304869 | 0.33859 | -0.88929 | 0.305798 | 0.746958 | 0.410948 | 1.357708 |
| rs10760335 | -0.15832 | 0.304545 | 0.603151 | -0.75523 | 0.438582 | 0.853572 | 0.469901 | 1.550508 |
| rs10774624 | -0.0814 | 0.152826 | 0.594272 | -0.38094 | 0.218135 | 0.921822 | 0.683218 | 1.243755 |
| rs10830227 | 0.009067 | 0.241363 | 0.970033 | -0.464 | 0.482138 | 1.009108 | 0.628761 | 1.619534 |
| rs10852506 | -0.04764 | 0.322899 | 0.882696 | -0.68053 | 0.585237 | 0.953473 | 0.506351 | 1.795416 |
| rs10911399 | 0.217392 | 0.311233 | 0.484873 | -0.39262 | 0.827408 | 1.242831 | 0.675282 | 2.287382 |
| rs11079322 | 0.263231 | 0.328944 | 0.423578 | -0.3815 | 0.907961 | 1.301127 | 0.682837 | 2.479263 |
| rs11139960 | 0.123379 | 0.165806 | 0.456808 | -0.2016 | 0.448359 | 1.131313 | 0.817421 | 1.56574 |
| rs11203203 | -0.22637 | 0.365861 | 0.5361 | -0.94345 | 0.490722 | 0.797426 | 0.389281 | 1.633495 |
| rs112169681 | 0.41631 | 0.361334 | 0.24926 | -0.2919 | 1.124525 | 1.516356 | 0.74684 | 3.078753 |
| rs11259536 | -0.28929 | 0.314356 | 0.357437 | -0.90543 | 0.326849 | 0.748795 | 0.404369 | 1.386592 |
| rs112789234 | 0.126157 | 0.377803 | 0.738438 | -0.61434 | 0.866651 | 1.13446 | 0.540999 | 2.378931 |
| rs114157359 | -0.25893 | 0.577199 | 0.653719 | -1.39024 | 0.872377 | 0.771875 | 0.249015 | 2.392591 |
| rs11555655 | -0.08869 | 0.47284 | 0.851209 | -1.01546 | 0.838073 | 0.915126 | 0.362236 | 2.311907 |
| rs115703708 | 0.028152 | 0.229189 | 0.90224 | -0.42106 | 0.477361 | 1.028552 | 0.656352 | 1.611816 |
| rs11571297 | 0.025779 | 0.200933 | 0.897913 | -0.36805 | 0.419608 | 1.026114 | 0.692083 | 1.521364 |
| rs1159619 | -0.04636 | 0.352766 | 0.895454 | -0.73778 | 0.645066 | 0.954702 | 0.478175 | 1.906114 |
| rs11596750 | -0.15614 | 0.343582 | 0.649496 | -0.82957 | 0.517276 | 0.855435 | 0.436239 | 1.677452 |
| rs117205793 | 0.269977 | 0.24002 | 0.26067 | -0.20046 | 0.740416 | 1.309934 | 0.818352 | 2.096807 |
| rs117267644 | -0.70409 | 0.731323 | 0.335669 | -2.13748 | 0.729307 | 0.49456 | 0.117952 | 2.073643 |
| rs11727369 | 0.28045 | 0.331691 | 0.397823 | -0.36966 | 0.930565 | 1.323726 | 0.690966 | 2.535941 |

| | | | | | | | | |
|---|---|---|---|---|---|---|---|---|
| rs117614175 | −0.48154 | 0.554251 | 0.384951 | −1.56787 | 0.604793 | 0.617832 | 0.208488 | 1.830873 |
| rs117748610 | −0.6619 | 0.332807 | 0.04672 | −1.3142 | −0.00959 | 0.515873 | 0.26869 | 0.990452 |
| rs11901096 | 0.119616 | 0.339454 | 0.724556 | −0.54571 | 0.784946 | 1.127063 | 0.579427 | 2.192289 |
| rs11989229 | −0.52886 | 0.342173 | 0.1222 | −1.19952 | 0.141794 | 0.589274 | 0.301338 | 1.152339 |
| rs12082589 | −0.49642 | 0.365428 | 0.174317 | −1.21266 | 0.219819 | 0.608706 | 0.297406 | 1.245851 |
| rs12123737 | 0.263987 | 0.355702 | 0.457992 | −0.43319 | 0.961164 | 1.302111 | 0.648438 | 2.614738 |
| rs12542501 | 0.03126 | 0.299246 | 0.916801 | −0.55526 | 0.617782 | 1.031754 | 0.573922 | 1.85481 |
| rs12697021 | 0.183187 | 0.342585 | 0.592844 | −0.48828 | 0.854654 | 1.201039 | 0.613681 | 2.350561 |
| rs12712117 | −0.56323 | 0.427957 | 0.188142 | −1.40203 | 0.275563 | 0.569365 | 0.246097 | 1.317272 |
| rs12722495 | −0.33594 | 0.245574 | 0.171319 | −0.81726 | 0.145385 | 0.714666 | 0.441638 | 1.156485 |
| rs12804463 | −0.26738 | 0.283001 | 0.344752 | −0.82207 | 0.287297 | 0.765379 | 0.439523 | 1.332821 |
| rs12907749 | −0.33627 | 0.305925 | 0.271683 | −0.93589 | 0.263342 | 0.714429 | 0.392238 | 1.301271 |
| rs13182737 | −1.12268 | 0.290769 | 0.000113 | −1.69258 | −0.55277 | 0.325408 | 0.184044 | 0.575354 |
| rs139025905 | −0.1373 | 0.319861 | 0.667731 | −0.76423 | 0.489623 | 0.871704 | 0.465691 | 1.631702 |
| rs140206868 | 0.90291 | 0.636213 | 0.155843 | −0.34407 | 2.149889 | 2.466772 | 0.708881 | 8.583903 |
| rs140837419 | −0.28343 | 0.264885 | 0.284621 | −0.8026 | 0.235749 | 0.753198 | 0.448161 | 1.265856 |
| rs142050319 | 0.369821 | 0.2989 | 0.215986 | −0.21602 | 0.955666 | 1.447476 | 0.805717 | 2.600401 |
| rs142359748 | −0.53886 | 0.395613 | 0.173172 | −1.31426 | 0.236544 | 0.583414 | 0.268673 | 1.266863 |
| rs143871332 | 0.564536 | 0.444327 | 0.203892 | −0.30634 | 1.435416 | 1.758631 | 0.736133 | 4.201392 |
| rs144091711 | −0.29124 | 0.490557 | 0.552721 | −1.25273 | 0.670255 | 0.747338 | 0.285724 | 1.954735 |
| rs144872936 | 0.240387 | 0.510515 | 0.637732 | −0.76022 | 1.240997 | 1.271742 | 0.467563 | 3.45906 |
| rs145435805 | −0.06146 | 0.097866 | 0.53002 | −0.25328 | 0.130361 | 0.940393 | 0.776254 | 1.139239 |
| rs145508614 | −0.01548 | 0.319764 | 0.961397 | −0.64221 | 0.61126 | 0.984643 | 0.526127 | 1.842752 |
| rs146624920 | −0.14732 | 0.336003 | 0.661057 | −0.80589 | 0.511244 | 0.863016 | 0.446691 | 1.667364 |
| rs146701335 | −0.06107 | 0.081934 | 0.456021 | −0.22166 | 0.099515 | 0.940753 | 0.801184 | 1.104636 |
| rs146775092 | −0.18651 | 0.239422 | 0.435981 | −0.65578 | 0.282758 | 0.829851 | 0.519039 | 1.326784 |
| rs147128719 | 0.724767 | 0.456041 | 0.112002 | −0.16907 | 1.618607 | 2.064249 | 0.844447 | 5.046056 |
| rs148348695 | −0.12141 | 0.526646 | 0.817676 | −1.15364 | 0.910816 | 0.88567 | 0.315487 | 2.486352 |
| rs148824391 | 0.082513 | 0.531698 | 0.876674 | −0.95962 | 1.124641 | 1.086012 | 0.38304 | 3.079113 |
| rs149687275 | 0.666546 | 0.48963 | 0.173411 | −0.29313 | 1.626221 | 1.947498 | 0.745926 | 5.084622 |
| rs1510889 | 0.479154 | 0.322752 | 0.137653 | −0.15344 | 1.111748 | 1.614708 | 0.857752 | 3.039667 |
| rs1543148 | 0.284905 | 0.423496 | 0.501109 | −0.54515 | 1.114957 | 1.329636 | 0.579756 | 3.049438 |
| rs1544218 | −0.01533 | 0.330142 | 0.962955 | −0.66241 | 0.631744 | 0.984783 | 0.515607 | 1.880888 |
| rs1634271 | −0.32088 | 0.355833 | 0.367172 | −1.01832 | 0.376549 | 0.725507 | 0.361202 | 1.457246 |
| rs17206070 | −0.03025 | 0.082923 | 0.715251 | −0.19278 | 0.132278 | 0.970201 | 0.824662 | 1.141426 |
| rs17392686 | 1.171049 | 0.795513 | 0.141003 | −0.38816 | 2.730255 | 3.225373 | 0.678306 | 15.33679 |
| rs17396905 | 0.007273 | 0.238542 | 0.975677 | −0.46027 | 0.474815 | 1.0073 | 0.631114 | 1.607716 |
| rs17600642 | 0.501167 | 0.425232 | 0.238568 | −0.33229 | 1.334622 | 1.650647 | 0.717281 | 3.79856 |
| rs184794035 | 0.304997 | 0.360891 | 0.398042 | −0.40235 | 1.012344 | 1.356621 | 0.668747 | 2.752045 |
| rs1869449 | −0.07075 | 0.242021 | 0.77002 | −0.54512 | 0.403606 | 0.93169 | 0.579775 | 1.497214 |
| rs189624574 | −0.27627 | 0.372734 | 0.458576 | −1.00683 | 0.45429 | 0.75861 | 0.365377 | 1.575055 |
| rs191398177 | −0.01208 | 0.12011 | 0.919902 | −0.24749 | 0.223337 | 0.987995 | 0.780755 | 1.250242 |
| rs192324744 | −0.08476 | 0.281276 | 0.763156 | −0.63606 | 0.466541 | 0.918733 | 0.529374 | 1.59447 |
| rs194749 | −0.14013 | 0.259236 | 0.588829 | −0.64823 | 0.367976 | 0.869249 | 0.522972 | 1.444808 |
| rs202520 | 0.059477 | 0.256774 | 0.816823 | −0.4438 | 0.562754 | 1.061282 | 0.641594 | 1.7555 |
| rs206763 | 0.110309 | 0.226793 | 0.626695 | −0.33421 | 0.554823 | 1.116623 | 0.715906 | 1.741633 |
| rs2111485 | 0.677926 | 0.244938 | 0.005644 | 0.197848 | 1.158005 | 1.969789 | 1.218777 | 3.183575 |

| | | | | | | | | |
|---|---|---|---|---|---|---|---|---|
| rs211510 | 0.019492 | 0.352377 | 0.955886 | -0.67117 | 0.710151 | 1.019684 | 0.511112 | 2.034298 |
| rs2269247 | -0.48186 | 0.243081 | 0.047445 | -0.9583 | -0.00542 | 0.617632 | 0.383544 | 0.994592 |
| rs2297764 | -0.66624 | 0.35981 | 0.064076 | -1.37147 | 0.038986 | 0.513635 | 0.253733 | 1.039756 |
| rs231971 | -0.29502 | 0.410728 | 0.472582 | -1.10005 | 0.510008 | 0.744517 | 0.332855 | 1.665304 |
| rs2414899 | -0.01335 | 0.35389 | 0.969908 | -0.70697 | 0.680273 | 0.986739 | 0.493134 | 1.974417 |
| rs2666237 | 0.081572 | 0.357368 | 0.819447 | -0.61887 | 0.782013 | 1.084991 | 0.538553 | 2.185868 |
| rs2872812 | 0.367429 | 0.308703 | 0.233954 | -0.23763 | 0.972488 | 1.444018 | 0.788495 | 2.644515 |
| rs28799272 | 0.438089 | 0.375916 | 0.243861 | -0.29871 | 1.174885 | 1.549743 | 0.741777 | 3.23777 |
| rs2949577 | 0.36442 | 0.358814 | 0.309809 | -0.33886 | 1.067694 | 1.439678 | 0.712586 | 2.908666 |
| rs2963801 | -0.22841 | 0.305374 | 0.45448 | -0.82694 | 0.370123 | 0.795799 | 0.437385 | 1.447912 |
| rs34664800 | 0.003724 | 0.300353 | 0.990107 | -0.58497 | 0.592417 | 1.003731 | 0.557123 | 1.808354 |
| rs35013225 | -0.078 | 0.327569 | 0.811793 | -0.72003 | 0.564037 | 0.924966 | 0.486735 | 1.757755 |
| rs362719 | 0.178678 | 0.32336 | 0.58056 | -0.45511 | 0.812465 | 1.195636 | 0.634379 | 2.253455 |
| rs372702 | -0.14156 | 0.340731 | 0.677806 | -0.80939 | 0.526273 | 0.868003 | 0.445128 | 1.692613 |
| rs39311 | 0.369864 | 0.321732 | 0.250307 | -0.26073 | 1.000458 | 1.447538 | 0.770489 | 2.719527 |
| rs4149965 | -0.11806 | 0.312941 | 0.705979 | -0.73142 | 0.495303 | 0.888642 | 0.481223 | 1.640995 |
| rs4514654 | 0.038588 | 0.389686 | 0.921119 | -0.7252 | 0.802373 | 1.039342 | 0.484229 | 2.230829 |
| rs4566101 | -0.19289 | 0.241422 | 0.424315 | -0.66607 | 0.280302 | 0.824576 | 0.513722 | 1.323529 |
| rs4762523 | 0.009115 | 0.319365 | 0.97723 | -0.61684 | 0.635071 | 1.009157 | 0.539647 | 1.887156 |
| rs5001561 | -0.81882 | 0.441303 | 0.063531 | -1.68377 | 0.046136 | 0.440952 | 0.185672 | 1.047217 |
| rs55916920 | 0.036573 | 0.408114 | 0.928594 | -0.76333 | 0.836476 | 1.03725 | 0.466111 | 2.308218 |
| rs56391297 | 0.744964 | 0.348982 | 0.032788 | 0.060958 | 1.428969 | 2.106365 | 1.062854 | 4.174395 |
| rs59680223 | -0.24297 | 0.23748 | 0.306256 | -0.70843 | 0.222493 | 0.784296 | 0.492417 | 1.249187 |
| rs60888743 | 0.053944 | 0.330303 | 0.87027 | -0.59345 | 0.701338 | 1.055425 | 0.552418 | 2.01645 |
| rs61868788 | 0.210133 | 0.404784 | 0.603674 | -0.58324 | 1.00351 | 1.233843 | 0.558086 | 2.727839 |
| rs61944737 | 0.03484 | 0.273046 | 0.898467 | -0.50033 | 0.570011 | 1.035454 | 0.60633 | 1.768286 |
| rs62123870 | -0.04036 | 0.332771 | 0.903465 | -0.69259 | 0.61187 | 0.960443 | 0.500278 | 1.843877 |
| rs62212655 | -0.6269 | 0.469164 | 0.18148 | -1.54646 | 0.292659 | 0.534244 | 0.213 | 1.339986 |
| rs62410259 | 0.069506 | 0.186649 | 0.709605 | -0.29633 | 0.435339 | 1.071978 | 0.743544 | 1.545486 |
| rs6434441 | -0.23801 | 0.380486 | 0.531609 | -0.98377 | 0.507738 | 0.788192 | 0.3739 | 1.661529 |
| rs6544107 | -0.06779 | 0.31974 | 0.832106 | -0.69448 | 0.558905 | 0.934461 | 0.499336 | 1.748756 |
| rs66535411 | 0.825959 | 0.464389 | 0.075307 | -0.08424 | 1.736162 | 2.284071 | 0.919207 | 5.675521 |
| rs6679677 | -0.14547 | 0.082616 | 0.078273 | -0.3074 | 0.016457 | 0.864616 | 0.735359 | 1.016593 |
| rs6719660 | -0.51381 | 0.33818 | 0.128674 | -1.17665 | 0.149018 | 0.598209 | 0.308311 | 1.160694 |
| rs71635549 | 0.238625 | 0.388733 | 0.539312 | -0.52329 | 1.000542 | 1.269502 | 0.592567 | 2.719756 |
| rs727251 | 0.309542 | 0.326544 | 0.343164 | -0.33048 | 0.949569 | 1.362801 | 0.718575 | 2.584594 |
| rs72842019 | 0.079966 | 0.312243 | 0.797872 | -0.53203 | 0.691962 | 1.08325 | 0.587411 | 1.997631 |
| rs72940100 | -0.13261 | 0.380985 | 0.727794 | -0.87934 | 0.614125 | 0.87581 | 0.415058 | 1.848038 |
| rs72958430 | -0.17866 | 0.273794 | 0.514047 | -0.7153 | 0.357972 | 0.836387 | 0.489045 | 1.430426 |
| rs73136665 | -0.18142 | 0.34734 | 0.60146 | -0.8622 | 0.49937 | 0.834088 | 0.42223 | 1.647683 |
| rs73261904 | -0.04983 | 0.251338 | 0.842848 | -0.54245 | 0.442794 | 0.951393 | 0.581322 | 1.557052 |
| rs73826337 | 0.328034 | 0.341878 | 0.337304 | -0.34205 | 0.998114 | 1.388236 | 0.710315 | 2.71316 |
| rs741172 | 0.00398 | 0.207185 | 0.984674 | -0.4021 | 0.410062 | 1.003988 | 0.668912 | 1.506912 |
| rs74388302 | -0.00839 | 0.333114 | 0.979916 | -0.66129 | 0.644517 | 0.991649 | 0.516186 | 1.905067 |
| rs75354435 | -0.15707 | 0.180483 | 0.384144 | -0.51082 | 0.196675 | 0.854643 | 0.600004 | 1.217348 |
| rs75575119 | 0.021222 | 0.473568 | 0.964256 | -0.90697 | 0.949415 | 1.021449 | 0.403745 | 2.584198 |
| rs76342469 | -0.12864 | 0.246507 | 0.601769 | -0.6118 | 0.354512 | 0.879289 | 0.542376 | 1.425485 |

| | | | | | | | | |
|---|---|---|---|---|---|---|---|---|
| rs76495645 | -0.14625 | 0.203794 | 0.472979 | -0.54569 | 0.253185 | 0.863941 | 0.579444 | 1.288122 |
| rs7670944 | -0.18818 | 0.118201 | 0.111375 | -0.41985 | 0.043493 | 0.828465 | 0.657143 | 1.044452 |
| rs7687596 | 0.341901 | 0.352326 | 0.331842 | -0.34866 | 1.032461 | 1.407621 | 0.705634 | 2.807967 |
| rs77064152 | -0.04897 | 0.175149 | 0.779772 | -0.39227 | 0.294317 | 0.952205 | 0.675525 | 1.342209 |
| rs77089863 | 0.033977 | 0.306544 | 0.911743 | -0.56685 | 0.634803 | 1.034561 | 0.567311 | 1.88665 |
| rs77181159 | -0.16652 | 0.334782 | 0.618917 | -0.82269 | 0.489657 | 0.84661 | 0.43925 | 1.631756 |
| rs77308817 | -0.09176 | 0.3455 | 0.790549 | -0.76895 | 0.585416 | 0.91232 | 0.463502 | 1.795739 |
| rs77523242 | -0.41842 | 0.343449 | 0.22312 | -1.09158 | 0.254745 | 0.658089 | 0.335687 | 1.290133 |
| rs7780389 | 0.144732 | 0.426153 | 0.73414 | -0.69053 | 0.979992 | 1.15573 | 0.501311 | 2.664436 |
| rs78387855 | 0.124128 | 0.187529 | 0.508029 | -0.24343 | 0.491685 | 1.13216 | 0.783935 | 1.635069 |
| rs79315483 | -0.04239 | 0.414018 | 0.918455 | -0.85386 | 0.769088 | 0.958498 | 0.425767 | 2.157798 |
| rs80004547 | 1.029425 | 0.4151 | 0.01314 | 0.215829 | 1.84302 | 2.799454 | 1.24089 | 6.315582 |
| rs80058612 | -0.51104 | 0.65072 | 0.432254 | -1.78645 | 0.764375 | 0.599873 | 0.167554 | 2.147652 |
| rs80348093 | -0.17969 | 0.330382 | 0.586531 | -0.82723 | 0.467864 | 0.835533 | 0.437257 | 1.59658 |
| rs8041124 | -0.43839 | 0.268891 | 0.103023 | -0.96542 | 0.088634 | 0.645072 | 0.380824 | 1.09268 |
| rs8056814 | -0.09241 | 0.262666 | 0.724978 | -0.60724 | 0.422416 | 0.911732 | 0.544855 | 1.525644 |
| rs8108092 | -0.47778 | 0.290128 | 0.0996 | -1.04643 | 0.090869 | 0.620158 | 0.351189 | 1.095125 |
| rs8133961 | -0.09718 | 0.256874 | 0.705206 | -0.60065 | 0.406297 | 0.907396 | 0.548456 | 1.501249 |
| rs860291 | 0.240356 | 0.4642 | 0.604608 | -0.66948 | 1.150187 | 1.271701 | 0.511977 | 3.158784 |
| rs9273363 | 0.014538 | 0.033456 | 0.663887 | -0.05104 | 0.080112 | 1.014645 | 0.950245 | 1.083409 |
| rs9382199 | -0.00734 | 0.330081 | 0.982261 | -0.6543 | 0.63962 | 0.992688 | 0.519807 | 1.89576 |
| rs9405098 | 0.124204 | 0.150541 | 0.409342 | -0.17086 | 0.419265 | 1.132247 | 0.842943 | 1.520843 |
| rs9501109 | 0.078005 | 0.186328 | 0.675476 | -0.2872 | 0.443207 | 1.081128 | 0.750364 | 1.557695 |
| All - Inverse variance weighted | -0.03082 | 0.017828 | 0.083863 | -0.06576 | 0.004124 | 0.969651 | 0.936354 | 1.004132 |
| All - MR Egger | -0.0244 | 0.026589 | 0.360454 | -0.07651 | 0.027719 | 0.975899 | 0.926342 | 1.028107 |

**Table 9:** ebi-a-GCST90000529

| SNP | b | se | p | lo_ci | up_ci | or | or_lci95 | or_uci95 |
|---|---|---|---|---|---|---|---|---|
| rs10225904 | 0.227424 | 0.392264 | 0.562067 | -0.54141 | 0.996262 | 1.255362 | 0.581925 | 2.708139 |
| rs10944479 | 0.050406 | 0.300505 | 0.86679 | -0.53858 | 0.639396 | 1.051698 | 0.583574 | 1.895336 |
| rs11058949 | -0.06521 | 0.224465 | 0.771432 | -0.50516 | 0.374744 | 0.936873 | 0.603409 | 1.454619 |
| rs11145876 | 0.012894 | 0.322851 | 0.968143 | -0.61989 | 0.645682 | 1.012977 | 0.538002 | 1.907286 |
| rs11620323 | -0.13904 | 0.385166 | 0.718101 | -0.89397 | 0.615882 | 0.870189 | 0.409028 | 1.851288 |
| rs11658622 | 0.415056 | 0.277328 | 0.13449 | -0.12851 | 0.958618 | 1.514456 | 0.879408 | 2.60809 |
| rs11765215 | 0.082335 | 0.354229 | 0.8162 | -0.61195 | 0.776624 | 1.08582 | 0.54229 | 2.17412 |
| rs118000057 | 0.229136 | 0.462747 | 0.620484 | -0.67785 | 1.136121 | 1.257513 | 0.507708 | 3.114663 |
| rs12140846 | -0.66098 | 0.352028 | 0.06043 | -1.35096 | 0.028991 | 0.516344 | 0.258992 | 1.029416 |
| rs12148251 | -0.28494 | 0.373028 | 0.444958 | -1.01607 | 0.446198 | 0.752062 | 0.362014 | 1.562361 |
| rs12150079 | -0.40744 | 0.339684 | 0.230348 | -1.07322 | 0.258342 | 0.665353 | 0.341907 | 1.294782 |
| rs12416116 | 0.132965 | 0.316635 | 0.674537 | -0.48764 | 0.753569 | 1.142209 | 0.614074 | 2.12457 |
| rs12610458 | -0.6934 | 0.477229 | 0.146231 | -1.62877 | 0.241968 | 0.499873 | 0.196171 | 1.273753 |
| rs12679857 | -0.26953 | 0.365215 | 0.460518 | -0.98535 | 0.446295 | 0.763741 | 0.373309 | 1.562512 |
| rs12722496 | -0.26125 | 0.189013 | 0.16691 | -0.63172 | 0.109211 | 0.770086 | 0.531677 | 1.115398 |
| rs12927355 | 0.015931 | 0.208323 | 0.939043 | -0.39238 | 0.424243 | 1.016059 | 0.675446 | 1.528434 |
| rs12982003 | -0.16067 | 0.327135 | 0.623315 | -0.80186 | 0.480509 | 0.851569 | 0.448494 | 1.616898 |

| | | | | | | | | |
|---|---|---|---|---|---|---|---|---|
| rs12982646 | 0.246266 | 0.216843 | 0.256088 | −0.17875 | 0.671278 | 1.27924 | 0.836318 | 1.956737 |
| rs1338373 | 0.298496 | 0.349515 | 0.393089 | −0.38655 | 0.983545 | 1.34783 | 0.679395 | 2.673918 |
| rs13415465 | −0.44671 | 0.285845 | 0.118102 | −1.00697 | 0.113541 | 0.639726 | 0.365324 | 1.120237 |
| rs144451833 | 0.285718 | 0.219814 | 0.193665 | −0.14512 | 0.716553 | 1.330716 | 0.86492 | 2.047364 |
| rs1487916 | −0.05442 | 0.294218 | 0.85327 | −0.63108 | 0.522252 | 0.947039 | 0.532016 | 1.68582 |
| rs151233 | 0.427467 | 0.261456 | 0.10206 | −0.08499 | 0.93992 | 1.533368 | 0.918525 | 2.559776 |
| rs1526913 | −0.51264 | 0.422749 | 0.225269 | −1.34123 | 0.315947 | 0.598912 | 0.261524 | 1.371558 |
| rs1534424 | 0.057268 | 0.356692 | 0.872445 | −0.64185 | 0.756385 | 1.05894 | 0.526319 | 2.130559 |
| rs1790946 | 0.340557 | 0.311438 | 0.274174 | −0.26986 | 0.950975 | 1.405731 | 0.763486 | 2.588232 |
| rs1857446 | 0.033499 | 0.031411 | 0.286219 | −0.02807 | 0.095064 | 1.034066 | 0.972323 | 1.09973 |
| rs187998805 | 0.050312 | 0.196448 | 0.797866 | −0.33473 | 0.43535 | 1.051599 | 0.715535 | 1.545504 |
| rs1950897 | 0.383807 | 0.366241 | 0.294656 | −0.33403 | 1.10164 | 1.467862 | 0.716035 | 3.009097 |
| rs2111485 | 0.689722 | 0.2492 | 0.005644 | 0.201291 | 1.178154 | 1.993162 | 1.22298 | 3.248371 |
| rs2269246 | −0.61298 | 0.299985 | 0.041015 | −1.20096 | −0.02501 | 0.541732 | 0.300907 | 0.975298 |
| rs2281808 | −0.00087 | 0.318933 | 0.997831 | −0.62598 | 0.624242 | 0.999133 | 0.534739 | 1.86683 |
| rs2289702 | 0.100807 | 0.218528 | 0.644581 | −0.32751 | 0.529121 | 1.106064 | 0.720719 | 1.69744 |
| rs231779 | −0.18784 | 0.203271 | 0.355434 | −0.58625 | 0.210569 | 0.828745 | 0.556407 | 1.23438 |
| rs2383983 | 0.079811 | 0.343553 | 0.816296 | −0.59355 | 0.753175 | 1.083083 | 0.552361 | 2.123733 |
| rs2476601 | −0.15566 | 0.079878 | 0.051324 | −0.31222 | 0.000898 | 0.855848 | 0.731818 | 1.000898 |
| rs280525 | 0.092632 | 0.357865 | 0.795753 | −0.60878 | 0.794049 | 1.097058 | 0.544012 | 2.212335 |
| rs28746962 | −0.01389 | 0.038613 | 0.71896 | −0.08958 | 0.061786 | 0.986201 | 0.914319 | 1.063735 |
| rs3024505 | −0.12928 | 0.297286 | 0.663651 | −0.71196 | 0.453398 | 0.878725 | 0.490679 | 1.573651 |
| rs3129880 | −0.02828 | 0.023912 | 0.236888 | −0.07515 | 0.018584 | 0.972113 | 0.927603 | 1.018758 |
| rs35672585 | 0.47502 | 0.423486 | 0.261994 | −0.35501 | 1.305051 | 1.608046 | 0.701165 | 3.687879 |
| rs3738676 | −0.23197 | 0.337962 | 0.492476 | −0.89438 | 0.430437 | 0.79297 | 0.408863 | 1.537929 |
| rs3814778 | −0.12968 | 0.300617 | 0.666192 | −0.71889 | 0.459529 | 0.878376 | 0.487293 | 1.583328 |
| rs3859570 | 0.282852 | 0.344559 | 0.411697 | −0.39248 | 0.958187 | 1.326909 | 0.675378 | 2.606966 |
| rs3918531 | −0.21565 | 0.389774 | 0.58007 | −0.97961 | 0.548301 | 0.806013 | 0.375457 | 1.730311 |
| rs4759229 | −0.15838 | 0.172458 | 0.35842 | −0.4964 | 0.179636 | 0.853524 | 0.608719 | 1.196781 |
| rs4820827 | −0.04964 | 0.244962 | 0.839421 | −0.52976 | 0.430489 | 0.951574 | 0.588744 | 1.538009 |
| rs4849134 | 0.186478 | 0.42181 | 0.658423 | −0.64027 | 1.013225 | 1.204999 | 0.527151 | 2.75447 |
| rs4939827 | −0.18171 | 0.363393 | 0.617042 | −0.89396 | 0.530536 | 0.83384 | 0.409031 | 1.699844 |
| rs56083426 | −0.16054 | 0.367923 | 0.662588 | −0.88167 | 0.560589 | 0.851683 | 0.41409 | 1.751704 |
| rs591001 | 0.213418 | 0.31684 | 0.500577 | −0.40759 | 0.834425 | 1.237902 | 0.665253 | 2.303488 |
| rs592625 | −0.44421 | 0.336968 | 0.18742 | −1.10467 | 0.21625 | 0.641332 | 0.331321 | 1.241412 |
| rs6000602 | −0.24429 | 0.36061 | 0.498137 | −0.95108 | 0.46251 | 0.783264 | 0.386323 | 1.588054 |
| rs6056608 | −0.33152 | 0.471355 | 0.481846 | −1.25538 | 0.592334 | 0.717831 | 0.284969 | 1.808204 |
| rs62397561 | 0.053494 | 0.191168 | 0.779609 | −0.3212 | 0.428184 | 1.054951 | 0.725281 | 1.534469 |
| rs6448432 | −0.79285 | 0.336345 | 0.018411 | −1.45209 | −0.13361 | 0.452552 | 0.234081 | 0.874927 |
| rs663743 | 0.210359 | 0.29903 | 0.481762 | −0.37574 | 0.796458 | 1.234121 | 0.686781 | 2.217671 |
| rs67140765 | 0.132783 | 0.140061 | 0.343109 | −0.14174 | 0.407303 | 1.142003 | 0.86785 | 1.50276 |
| rs6829631 | 0.383293 | 0.331183 | 0.247131 | −0.26582 | 1.032411 | 1.467108 | 0.766573 | 2.807828 |
| rs6871748 | 0.895162 | 0.310469 | 0.003936 | 0.286643 | 1.503682 | 2.447733 | 1.331948 | 4.498222 |
| rs706911 | 0.794567 | 0.442612 | 0.072626 | −0.07295 | 1.662087 | 2.213481 | 0.929644 | 5.270297 |
| rs709166 | −0.3323 | 0.34474 | 0.335082 | −1.008 | 0.343386 | 0.717269 | 0.36495 | 1.409713 |
| rs71479319 | −0.16648 | 0.432634 | 0.700377 | −1.01445 | 0.681479 | 0.846637 | 0.362604 | 1.9768 |
| rs719654 | −0.00965 | 0.067364 | 0.886118 | −0.14168 | 0.122386 | 0.990399 | 0.867898 | 1.13019 |

| | | | | | | | | |
|---|---|---|---|---|---|---|---|---|
| rs7221403 | 0.343123 | 0.360247 | 0.340862 | -0.36296 | 1.049207 | 1.409341 | 0.695613 | 2.855385 |
| rs7237497 | -0.06888 | 0.216885 | 0.750815 | -0.49397 | 0.35622 | 0.933443 | 0.610199 | 1.427922 |
| rs72661832 | -0.33939 | 0.325304 | 0.296813 | -0.97698 | 0.298209 | 0.712207 | 0.376445 | 1.347443 |
| rs72698768 | -0.2856 | 0.322402 | 0.375694 | -0.91751 | 0.346305 | 0.751561 | 0.399512 | 1.413834 |
| rs73135029 | -0.33826 | 0.279634 | 0.226408 | -0.88635 | 0.20982 | 0.713008 | 0.412159 | 1.233455 |
| rs77027760 | -0.16026 | 0.393849 | 0.684078 | -0.9322 | 0.611684 | 0.851923 | 0.393686 | 1.843534 |
| rs78037977 | 0.183929 | 0.27805 | 0.508293 | -0.36105 | 0.728906 | 1.201931 | 0.696946 | 2.072812 |
| rs80054410 | -0.16319 | 0.25791 | 0.526901 | -0.66869 | 0.342313 | 0.849429 | 0.512377 | 1.408201 |
| rs8007228 | 0.080883 | 0.380043 | 0.831462 | -0.664 | 0.825768 | 1.084245 | 0.514787 | 2.283635 |
| rs8033923 | -0.00078 | 0.451426 | 0.99863 | -0.88557 | 0.884019 | 0.999225 | 0.412479 | 2.420609 |
| rs8097275 | 0.069387 | 0.422723 | 0.869619 | -0.75915 | 0.897924 | 1.071851 | 0.468064 | 2.454502 |
| rs85360 | -0.45841 | 0.377854 | 0.225059 | -1.199 | 0.282187 | 0.63229 | 0.301495 | 1.326026 |
| rs883242 | -0.00906 | 0.297659 | 0.975726 | -0.59247 | 0.574355 | 0.990984 | 0.55296 | 1.775985 |
| rs9266775 | 0.01658 | 0.062933 | 0.792199 | -0.10677 | 0.139928 | 1.016718 | 0.898734 | 1.150191 |
| rs9375435 | -0.03304 | 0.360107 | 0.926897 | -0.73885 | 0.67277 | 0.9675 | 0.477663 | 1.959658 |
| rs9468204 | -0.0219 | 0.151781 | 0.885298 | -0.31939 | 0.275595 | 0.978343 | 0.726595 | 1.317314 |
| rs9517712 | 0.547891 | 0.356898 | 0.124748 | -0.15163 | 1.247411 | 1.729601 | 0.859307 | 3.481319 |
| rs9600451 | -0.60176 | 0.290098 | 0.038048 | -1.17035 | -0.03317 | 0.547846 | 0.310257 | 0.967376 |
| rs9911533 | 0.316931 | 0.30776 | 0.303105 | -0.28628 | 0.920141 | 1.372908 | 0.751053 | 2.509644 |
| All - Inverse variance weighted | -0.01034 | 0.014723 | 0.482455 | -0.0392 | 0.018516 | 0.989712 | 0.96156 | 1.018689 |
| All - MR Egger | -0.00927 | 0.01841 | 0.615952 | -0.04535 | 0.026814 | 0.990772 | 0.955658 | 1.027177 |

**Table 10:** ebi-a-GCST90014023

| SNP | b | se | p | lo_ci | up_ci | or | or_lci95 | or_uci95 |
|---|---|---|---|---|---|---|---|---|
| rs10004996 | -0.07625 | 0.522483 | 0.883977 | -1.10031 | 0.947821 | 0.926588 | 0.332767 | 2.580081 |
| rs10023871 | 1.12844 | 0.49385 | 0.022314 | 0.160493 | 2.096386 | 3.09083 | 1.174089 | 8.136714 |
| rs10224046 | -0.21784 | 0.457141 | 0.6337 | -1.11383 | 0.678157 | 0.804255 | 0.328298 | 1.970242 |
| rs10440734 | 0.834487 | 0.601051 | 0.165021 | -0.34357 | 2.012547 | 2.303632 | 0.709232 | 7.482351 |
| rs1050979 | -0.24676 | 0.359532 | 0.492498 | -0.95145 | 0.457922 | 0.781327 | 0.386183 | 1.580785 |
| rs1075163 | 0.25935 | 0.770257 | 0.736338 | -1.25035 | 1.769053 | 1.296088 | 0.286404 | 5.865299 |
| rs10751776 | -0.1081 | 0.490978 | 0.825732 | -1.07042 | 0.854214 | 0.897535 | 0.342864 | 2.349528 |
| rs10801128 | -0.44703 | 0.456819 | 0.327797 | -1.34239 | 0.44834 | 0.639527 | 0.26122 | 1.565711 |
| rs10942481 | 0.264315 | 0.493608 | 0.592322 | -0.70316 | 1.231787 | 1.302538 | 0.49502 | 3.427348 |
| rs1094791 | 0.302048 | 0.55515 | 0.586383 | -0.78604 | 1.390142 | 1.352627 | 0.455643 | 4.015419 |
| rs11022095 | 0.554236 | 0.555055 | 0.318025 | -0.53367 | 1.642144 | 1.740611 | 0.586448 | 5.166237 |
| rs11136791 | -0.36234 | 0.564172 | 0.520705 | -1.46812 | 0.743432 | 0.696043 | 0.230358 | 2.103142 |
| rs11203203 | -0.1826 | 0.295125 | 0.5361 | -0.76105 | 0.395845 | 0.833101 | 0.467177 | 1.485639 |
| rs11240547 | 0.149758 | 0.551343 | 0.785911 | -0.93088 | 1.230391 | 1.161553 | 0.394209 | 3.422568 |
| rs11245318 | 1.067467 | 0.607592 | 0.078938 | -0.12341 | 2.258347 | 2.908005 | 0.883899 | 9.567262 |
| rs112485592 | -0.01262 | 0.754614 | 0.986657 | -1.49166 | 1.466423 | 0.98746 | 0.224998 | 4.333707 |
| rs11264545 | -0.12235 | 0.553294 | 0.824995 | -1.2068 | 0.962108 | 0.884841 | 0.299152 | 2.617208 |
| rs112923750 | 0.475552 | 0.504765 | 0.346128 | -0.51379 | 1.464892 | 1.608903 | 0.598226 | 4.327074 |
| rs114378220 | -0.33778 | 0.462436 | 0.465119 | -1.24416 | 0.568591 | 0.71335 | 0.288183 | 1.765777 |
| rs115696001 | 0.504348 | 0.466401 | 0.279536 | -0.4098 | 1.418494 | 1.655906 | 0.663785 | 4.130893 |
| rs116495604 | 0.630037 | 0.529696 | 0.23427 | -0.40817 | 1.668242 | 1.87768 | 0.664867 | 5.302836 |

| | | | | | | | | |
|---|---|---|---|---|---|---|---|---|
| rs116592029 | −0.25668 | 0.632795 | 0.685016 | −1.49696 | 0.983599 | 0.773617 | 0.22381 | 2.674063 |
| rs116626221 | −0.07951 | 0.127944 | 0.534285 | −0.33029 | 0.171256 | 0.923565 | 0.718719 | 1.186795 |
| rs11756073 | 0.758644 | 0.566005 | 0.180132 | −0.35072 | 1.868013 | 2.135379 | 0.704177 | 6.475417 |
| rs11783245 | −0.67584 | 0.528883 | 0.2013 | −1.71245 | 0.360774 | 0.508731 | 0.180424 | 1.434439 |
| rs117918837 | −0.26655 | 0.543787 | 0.624011 | −1.33237 | 0.799273 | 0.766018 | 0.263851 | 2.223923 |
| rs11940117 | −0.49205 | 0.487196 | 0.312511 | −1.44696 | 0.462853 | 0.61137 | 0.235285 | 1.588599 |
| rs12128789 | −0.97146 | 0.469997 | 0.03874 | −1.89265 | −0.05026 | 0.378531 | 0.150672 | 0.95098 |
| rs12137048 | 0.526478 | 0.562755 | 0.349511 | −0.57652 | 1.629477 | 1.69296 | 0.56185 | 5.101208 |
| rs12225187 | −0.14824 | 0.468599 | 0.751741 | −1.06669 | 0.770216 | 0.862225 | 0.344145 | 2.160233 |
| rs12257077 | 0.456654 | 0.611845 | 0.455452 | −0.74256 | 1.65587 | 1.578783 | 0.475894 | 5.237632 |
| rs12464462 | 0.034033 | 0.445796 | 0.939146 | −0.83973 | 0.907794 | 1.034619 | 0.431828 | 2.478848 |
| rs12597485 | 0.689512 | 0.548079 | 0.208372 | −0.38472 | 1.763746 | 1.992743 | 0.68064 | 5.834254 |
| rs12722572 | −0.45804 | 0.523413 | 0.381518 | −1.48393 | 0.56785 | 0.632522 | 0.226745 | 1.764469 |
| rs12742756 | −0.72743 | 0.460897 | 0.114499 | −1.63079 | 0.175929 | 0.483149 | 0.195775 | 1.192354 |
| rs12793625 | 0.672588 | 0.656677 | 0.305726 | −0.6145 | 1.959675 | 1.959302 | 0.540912 | 7.09702 |
| rs12927355 | 0.015601 | 0.204009 | 0.939043 | −0.38426 | 0.415458 | 1.015723 | 0.680957 | 1.515065 |
| rs13032694 | −0.05611 | 0.659377 | 0.932186 | −1.34849 | 1.236269 | 0.945436 | 0.259633 | 3.442743 |
| rs13086363 | −0.24499 | 0.550941 | 0.656559 | −1.32483 | 0.834858 | 0.782715 | 0.265848 | 2.304487 |
| rs13147049 | 0.305053 | 0.34999 | 0.383424 | −0.38093 | 0.991033 | 1.356697 | 0.683227 | 2.694016 |
| rs13259300 | 0.127468 | 0.419028 | 0.760976 | −0.69383 | 0.948763 | 1.135948 | 0.49966 | 2.582512 |
| rs13322900 | −0.95898 | 0.546092 | 0.079075 | −2.02932 | 0.11136 | 0.383284 | 0.131425 | 1.117797 |
| rs13421651 | 0.312094 | 0.577899 | 0.589163 | −0.82059 | 1.444777 | 1.366283 | 0.440172 | 4.240907 |
| rs1350275 | 0.033531 | 0.419206 | 0.936248 | −0.78811 | 0.855174 | 1.034099 | 0.454702 | 2.351783 |
| rs138413706 | −0.01228 | 0.64666 | 0.984854 | −1.27973 | 1.255178 | 0.987799 | 0.278113 | 3.508461 |
| rs1389999 | 0.629539 | 0.526047 | 0.231409 | −0.40151 | 1.660591 | 1.876746 | 0.669307 | 5.262421 |
| rs141094656 | 0.41857 | 0.443785 | 0.345588 | −0.45125 | 1.288389 | 1.519787 | 0.636832 | 3.626938 |
| rs142770866 | 1.140942 | 0.513622 | 0.026326 | 0.134244 | 2.147641 | 3.129717 | 1.143672 | 8.564629 |
| rs1529969 | −0.55286 | 0.532485 | 0.29915 | −1.59653 | 0.490814 | 0.575304 | 0.202599 | 1.633645 |
| rs1574285 | 0.06424 | 0.303353 | 0.832288 | −0.53033 | 0.658812 | 1.066349 | 0.58841 | 1.932495 |
| rs1701704 | −0.12025 | 0.167414 | 0.472578 | −0.44838 | 0.207879 | 0.886697 | 0.63866 | 1.231065 |
| rs17105278 | 0.647186 | 0.464764 | 0.163769 | −0.26375 | 1.558123 | 1.910159 | 0.768165 | 4.749897 |
| rs172032 | −0.38525 | 0.508839 | 0.448981 | −1.38257 | 0.612075 | 0.680281 | 0.250932 | 1.844254 |
| rs17424046 | 0.377044 | 0.565729 | 0.505108 | −0.73178 | 1.485872 | 1.457968 | 0.48105 | 4.418817 |
| rs17623914 | −0.17263 | 0.551961 | 0.754468 | −1.25447 | 0.909217 | 0.841451 | 0.285227 | 2.482377 |
| rs1782648 | 0.383797 | 0.522769 | 0.462849 | −0.64083 | 1.408424 | 1.467848 | 0.526855 | 4.089505 |
| rs182429 | 0.775992 | 0.544805 | 0.154346 | −0.29183 | 1.84381 | 2.172747 | 0.746899 | 6.320573 |
| rs1893208 | 0.135524 | 0.506971 | 0.789222 | −0.85814 | 1.129186 | 1.145136 | 0.42395 | 3.093139 |
| rs1947178 | −0.62747 | 0.519665 | 0.227261 | −1.64601 | 0.391076 | 0.533943 | 0.192818 | 1.478571 |
| rs202535 | 0.467062 | 0.329536 | 0.156386 | −0.17883 | 1.112953 | 1.595301 | 0.836249 | 3.043333 |
| rs2044970 | 0.721816 | 0.479613 | 0.132325 | −0.21823 | 1.661858 | 2.058168 | 0.803944 | 5.26909 |
| rs2111485 | 0.837641 | 0.302644 | 0.005644 | 0.24446 | 1.430823 | 2.31091 | 1.276932 | 4.182138 |
| rs218265 | 0.105584 | 0.524692 | 0.840519 | −0.92281 | 1.133979 | 1.111359 | 0.3974 | 3.108 |
| rs2188962 | −0.00495 | 0.516051 | 0.992344 | −1.01641 | 1.006508 | 0.995061 | 0.361891 | 2.73603 |
| rs2290708 | −0.29397 | 0.609833 | 0.629768 | −1.48924 | 0.9013 | 0.745297 | 0.225543 | 2.462802 |
| rs229527 | −0.30812 | 0.374733 | 0.410944 | −1.04259 | 0.426359 | 0.734829 | 0.352539 | 1.53167 |
| rs231972 | 0.081014 | 0.388019 | 0.834613 | −0.6795 | 0.841532 | 1.084386 | 0.506869 | 2.319918 |
| rs238265 | −0.36998 | 0.451171 | 0.412192 | −1.25427 | 0.514315 | 0.690748 | 0.285283 | 1.672493 |

| | | | | | | | | |
|---|---|---|---|---|---|---|---|---|
| rs2421471 | −0.13488 | 0.574417 | 0.814358 | −1.26073 | 0.990979 | 0.873823 | 0.283446 | 2.693871 |
| rs2476601 | −0.16254 | 0.083409 | 0.051324 | −0.32602 | 0.000938 | 0.849979 | 0.721787 | 1.000938 |
| rs2478151 | 0.328595 | 0.587064 | 0.575667 | −0.82205 | 1.479241 | 1.389015 | 0.439529 | 4.389612 |
| rs2493411 | 0.360168 | 0.402749 | 0.371176 | −0.42922 | 1.149556 | 1.43357 | 0.651016 | 3.156791 |
| rs2543537 | −0.83264 | 0.459555 | 0.07001 | −1.73337 | 0.068085 | 0.434899 | 0.176688 | 1.070456 |
| rs2596560 | 0.106878 | 0.058172 | 0.066168 | −0.00714 | 0.220895 | 1.112798 | 0.992887 | 1.247192 |
| rs2608053 | −0.02869 | 0.532447 | 0.957026 | −1.07229 | 1.014905 | 0.971716 | 0.342225 | 2.7591 |
| rs2611211 | 0.006919 | 0.31099 | 0.98225 | −0.60262 | 0.61646 | 1.006943 | 0.547374 | 1.852359 |
| rs2649751 | 0.297542 | 0.578809 | 0.60721 | −0.83692 | 1.432008 | 1.346545 | 0.433041 | 4.187098 |
| rs2793108 | −0.23881 | 0.626109 | 0.702897 | −1.46598 | 0.988368 | 0.787568 | 0.230852 | 2.686847 |
| rs28406450 | −0.27906 | 0.544076 | 0.608021 | −1.34545 | 0.787332 | 0.756497 | 0.260424 | 2.197526 |
| rs28427413 | −0.12879 | 0.471115 | 0.78456 | −1.05218 | 0.794591 | 0.879155 | 0.349176 | 2.213536 |
| rs3024493 | −0.14608 | 0.32141 | 0.649476 | −0.77604 | 0.483886 | 0.86409 | 0.460224 | 1.622367 |
| rs3087243 | 0.143724 | 0.203792 | 0.480656 | −0.25571 | 0.543157 | 1.154566 | 0.774367 | 1.721434 |
| rs3098944 | 0.613548 | 0.546629 | 0.261684 | −0.45785 | 1.684941 | 1.846972 | 0.632645 | 5.392131 |
| rs3181261 | 0.727159 | 0.509472 | 0.153499 | −0.2714 | 1.725724 | 2.069194 | 0.762308 | 5.616584 |
| rs3184504 | −0.16444 | 0.167679 | 0.326751 | −0.49309 | 0.164212 | 0.848369 | 0.610736 | 1.178464 |
| rs34593439 | 0.131969 | 0.261955 | 0.614412 | −0.38146 | 0.6454 | 1.141073 | 0.682862 | 1.906749 |
| rs34908535 | −0.00484 | 0.567215 | 0.993199 | −1.11658 | 1.106906 | 0.995177 | 0.327399 | 3.024985 |
| rs34976781 | 0.085553 | 0.046775 | 0.067396 | −0.00613 | 0.177233 | 1.08932 | 0.993892 | 1.193909 |
| rs35327136 | 0.760042 | 0.719936 | 0.291102 | −0.65103 | 2.171117 | 2.138367 | 0.521507 | 8.768072 |
| rs35810604 | −0.50393 | 0.504173 | 0.317548 | −1.49211 | 0.484254 | 0.604154 | 0.224898 | 1.622964 |
| rs36104352 | −0.30201 | 0.657803 | 0.646145 | −1.59131 | 0.987281 | 0.739328 | 0.203659 | 2.683928 |
| rs3802214 | 0.460096 | 0.405446 | 0.256463 | −0.33458 | 1.254771 | 1.584226 | 0.71564 | 3.507034 |
| rs3830119 | 0.544666 | 0.489664 | 0.265999 | −0.41508 | 1.504408 | 1.724032 | 0.66029 | 4.501488 |
| rs4073745 | 0.835926 | 0.503223 | 0.096684 | −0.15039 | 1.822244 | 2.30695 | 0.860372 | 6.185723 |
| rs41453248 | −0.07663 | 0.632325 | 0.903547 | −1.31598 | 1.162732 | 0.926236 | 0.26821 | 3.198659 |
| rs4548024 | −0.32409 | 0.480121 | 0.499666 | −1.26513 | 0.616947 | 0.723186 | 0.282204 | 1.853262 |
| rs4794674 | 0.417109 | 0.529307 | 0.430681 | −0.62033 | 1.454551 | 1.517568 | 0.537765 | 4.282561 |
| rs4820827 | −0.06407 | 0.316165 | 0.839421 | −0.68375 | 0.555618 | 0.937943 | 0.504721 | 1.743017 |
| rs548591 | −0.60586 | 0.585454 | 0.300736 | −1.75335 | 0.541629 | 0.545605 | 0.173193 | 1.718805 |
| rs55728265 | −0.33784 | 0.538524 | 0.530438 | −1.39334 | 0.717671 | 0.713312 | 0.248244 | 2.049654 |
| rs55893453 | 0.268978 | 0.551532 | 0.625768 | −0.81203 | 1.349982 | 1.308627 | 0.443958 | 3.857356 |
| rs559503 | −0.20312 | 0.533202 | 0.70324 | −1.2482 | 0.841953 | 0.816177 | 0.287021 | 2.320894 |
| rs56152581 | −0.31233 | 0.497516 | 0.530155 | −1.28746 | 0.662806 | 0.731743 | 0.275972 | 1.940229 |
| rs56994090 | −0.22401 | 0.285758 | 0.433093 | −0.7841 | 0.336078 | 0.799308 | 0.456533 | 1.399448 |
| rs57209021 | 0.238122 | 0.465661 | 0.609097 | −0.67457 | 1.150817 | 1.268864 | 0.509374 | 3.160775 |
| rs574384 | 0.303533 | 0.432279 | 0.482574 | −0.54373 | 1.150801 | 1.354636 | 0.580576 | 3.160722 |
| rs59896527 | 0.861457 | 0.524225 | 0.100321 | −0.16602 | 1.888937 | 2.366607 | 0.847027 | 6.612339 |
| rs601338 | 0.115892 | 0.310127 | 0.708634 | −0.49196 | 0.723741 | 1.122875 | 0.611429 | 2.062133 |
| rs607703 | −0.59923 | 0.415995 | 0.149732 | −1.41458 | 0.216118 | 0.549233 | 0.243027 | 1.241249 |
| rs61476910 | 0.642372 | 0.49288 | 0.192472 | −0.32367 | 1.608416 | 1.900984 | 0.723487 | 4.994893 |
| rs61759532 | 1.169228 | 0.397037 | 0.003231 | 0.391034 | 1.947421 | 3.219505 | 1.478509 | 7.010585 |
| rs61839660 | −0.34532 | 0.268717 | 0.19877 | −0.872 | 0.181367 | 0.707995 | 0.418113 | 1.198855 |
| rs61951270 | 0.177643 | 0.53897 | 0.741704 | −0.87874 | 1.234023 | 1.194399 | 0.415307 | 3.435021 |
| rs61987801 | 0.723051 | 0.546097 | 0.185492 | −0.3473 | 1.793402 | 2.060711 | 0.706593 | 6.009864 |
| rs62284579 | 0.427365 | 0.55448 | 0.440856 | −0.65942 | 1.514146 | 1.533211 | 0.517153 | 4.545537 |

| | | | | | | | | |
|---|---|---|---|---|---|---|---|---|
| rs62407874 | -0.09691 | 0.247677 | 0.695587 | -0.58236 | 0.388535 | 0.907636 | 0.558579 | 1.474818 |
| rs62556069 | -1.21598 | 0.793515 | 0.125426 | -2.77127 | 0.339313 | 0.296421 | 0.062583 | 1.403983 |
| rs6434435 | 0.056954 | 0.503482 | 0.909935 | -0.92987 | 1.043779 | 1.058607 | 0.394605 | 2.839929 |
| rs6582583 | 1.034382 | 0.583532 | 0.076291 | -0.10934 | 2.178104 | 2.813366 | 0.896425 | 8.82955 |
| rs6602699 | 0.720761 | 0.570497 | 0.206449 | -0.39741 | 1.838935 | 2.055997 | 0.672056 | 6.289837 |
| rs663743 | 0.277656 | 0.394694 | 0.481762 | -0.49594 | 1.051256 | 1.320032 | 0.608995 | 2.861244 |
| rs6841698 | 0.732377 | 0.588879 | 0.213617 | -0.42183 | 1.88658 | 2.08002 | 0.655848 | 6.596771 |
| rs6908626 | 0.140001 | 0.29428 | 0.634259 | -0.43679 | 0.71679 | 1.150275 | 0.646108 | 2.047849 |
| rs6944602 | -0.35407 | 0.581592 | 0.542662 | -1.49399 | 0.785851 | 0.701827 | 0.224476 | 2.194274 |
| rs7068821 | 0.058181 | 0.2744 | 0.832085 | -0.47964 | 0.596005 | 1.059906 | 0.619004 | 1.814854 |
| rs706911 | 1.080909 | 0.602119 | 0.072626 | -0.09924 | 2.261063 | 2.947359 | 0.905521 | 9.593285 |
| rs7130222 | 0.260955 | 0.470959 | 0.579516 | -0.66213 | 1.184035 | 1.298169 | 0.515754 | 3.267532 |
| rs71624119 | 0.186238 | 0.606337 | 0.758727 | -1.00218 | 1.37466 | 1.204709 | 0.367077 | 3.953731 |
| rs722988 | 0.113649 | 0.500776 | 0.820465 | -0.86787 | 1.09517 | 1.120359 | 0.419845 | 2.989689 |
| rs7237497 | -0.07926 | 0.249602 | 0.750815 | -0.56849 | 0.409955 | 0.923795 | 0.566383 | 1.506751 |
| rs725351 | 0.3192 | 0.531955 | 0.548472 | -0.72343 | 1.361832 | 1.376027 | 0.485085 | 3.903338 |
| rs72789002 | -0.16413 | 0.459549 | 0.720975 | -1.06485 | 0.736586 | 0.848631 | 0.344781 | 2.088792 |
| rs736374 | 0.514772 | 0.572016 | 0.36816 | -0.60638 | 1.635924 | 1.673257 | 0.545321 | 5.1342 |
| rs74480102 | -1.04943 | 0.671509 | 0.118102 | -2.36559 | 0.266729 | 0.350138 | 0.093894 | 1.305687 |
| rs7668577 | -0.74868 | 0.440076 | 0.088897 | -1.61123 | 0.113871 | 0.472991 | 0.199642 | 1.120607 |
| rs7684253 | 0.474355 | 0.498572 | 0.341387 | -0.50284 | 1.451555 | 1.606978 | 0.604808 | 4.269751 |
| rs7688335 | -0.45315 | 0.494801 | 0.359755 | -1.42296 | 0.516655 | 0.63562 | 0.240999 | 1.676411 |
| rs77580539 | 0.521561 | 0.685797 | 0.446945 | -0.8226 | 1.865723 | 1.684655 | 0.439288 | 6.460603 |
| rs7776597 | 0.115853 | 0.498492 | 0.816223 | -0.86119 | 1.092898 | 1.12283 | 0.422658 | 2.982905 |
| rs7795896 | 0.366334 | 0.295522 | 0.215116 | -0.21289 | 0.945557 | 1.442438 | 0.808246 | 2.574247 |
| rs78526882 | -0.71464 | 0.568049 | 0.208369 | -1.82802 | 0.398735 | 0.489368 | 0.160732 | 1.489938 |
| rs78885459 | -0.28341 | 0.528852 | 0.592034 | -1.31996 | 0.753144 | 0.753213 | 0.267147 | 2.123665 |
| rs79318397 | -0.04381 | 0.939785 | 0.96282 | -1.88579 | 1.798171 | 0.957138 | 0.15171 | 6.03859 |
| rs79879717 | 0.929105 | 0.746044 | 0.212994 | -0.53314 | 2.391352 | 2.532241 | 0.586758 | 10.92826 |
| rs8043362 | -0.37015 | 0.605625 | 0.541071 | -1.55718 | 0.81687 | 0.690628 | 0.21073 | 2.263405 |
| rs844289 | -0.02327 | 0.479987 | 0.961341 | -0.96404 | 0.91751 | 0.977003 | 0.381349 | 2.503049 |
| rs855330 | -0.39321 | 0.359879 | 0.274558 | -1.09858 | 0.31215 | 0.674885 | 0.333346 | 1.366359 |
| rs9268145 | 0.030027 | 0.063816 | 0.637986 | -0.09505 | 0.155107 | 1.030482 | 0.909324 | 1.167783 |
| rs9277196 | 0.303735 | 0.153934 | 0.048478 | 0.002025 | 0.605445 | 1.35491 | 1.002027 | 1.832067 |
| rs9286452 | -0.38033 | 0.534639 | 0.476847 | -1.42823 | 0.667559 | 0.683634 | 0.239734 | 1.949473 |
| rs9376293 | -0.8805 | 0.592285 | 0.137114 | -2.04138 | 0.280374 | 0.414574 | 0.129849 | 1.323625 |
| rs9378200 | -0.11079 | 0.127 | 0.383032 | -0.35971 | 0.138135 | 0.895131 | 0.697882 | 1.148131 |
| rs9405031 | -0.01678 | 0.348937 | 0.961656 | -0.70069 | 0.667141 | 0.983365 | 0.496242 | 1.948658 |
| rs9517712 | 0.640154 | 0.416998 | 0.124748 | -0.17716 | 1.45747 | 1.896772 | 0.837643 | 4.295081 |
| rs9866625 | -0.48748 | 0.542267 | 0.368668 | -1.55032 | 0.57536 | 0.614171 | 0.212179 | 1.777771 |
| rs9897747 | 0.818033 | 0.481936 | 0.089623 | -0.12656 | 1.762629 | 2.266039 | 0.881119 | 5.827737 |
| All - Inverse variance weighted | 0.047997 | 0.022118 | 0.030002 | 0.004646 | 0.091347 | 1.049167 | 1.004657 | 1.095649 |
| All - MR Egger | 0.026014 | 0.030735 | 0.398637 | -0.03423 | 0.086254 | 1.026356 | 0.966353 | 1.090084 |

**Table 11:** ebi-a-GCST90018925

| SNP | b | se | p | lo_ci | up_ci | or | or_lci95 | or_uci95 |
|---|---|---|---|---|---|---|---|---|
| rs1023004 | 0.456563 | 0.48638 | 0.347886 | -0.49674 | 1.409868 | 1.57864 | 0.608511 | 4.095413 |
| rs10420439 | 0.002531 | 0.450887 | 0.995522 | -0.88121 | 0.88627 | 1.002534 | 0.414282 | 2.426064 |
| rs10503778 | 0.503856 | 0.594308 | 0.396548 | -0.66099 | 1.6687 | 1.655091 | 0.516341 | 5.305265 |
| rs10811662 | -0.77757 | 0.385511 | 0.043697 | -1.53317 | -0.02197 | 0.459521 | 0.21585 | 0.978271 |
| rs10876866 | -0.28238 | 0.369942 | 0.445276 | -1.00747 | 0.442705 | 0.753986 | 0.365142 | 1.556912 |
| rs11219480 | 0.122407 | 0.46673 | 0.793117 | -0.79238 | 1.037198 | 1.130214 | 0.452764 | 2.821299 |
| rs11257600 | 0.36636 | 0.489493 | 0.45419 | -0.59305 | 1.325766 | 1.442474 | 0.552641 | 3.76507 |
| rs115335034 | 0.257143 | 0.288945 | 0.373499 | -0.30919 | 0.823476 | 1.293231 | 0.734042 | 2.278407 |
| rs115380430 | 0.08662 | 0.14975 | 0.562973 | -0.20689 | 0.380131 | 1.090482 | 0.813109 | 1.462476 |
| rs116490751 | 0.09589 | 0.575163 | 0.867592 | -1.03143 | 1.22321 | 1.100638 | 0.356497 | 3.398079 |
| rs11784281 | -0.23606 | 0.389913 | 0.5449 | -1.00029 | 0.528168 | 0.789733 | 0.367773 | 1.695823 |
| rs12331396 | -0.80191 | 0.452572 | 0.076413 | -1.68895 | 0.085134 | 0.448473 | 0.184713 | 1.088863 |
| rs12405018 | 0.399157 | 0.54809 | 0.466449 | -0.6751 | 1.473413 | 1.490567 | 0.509106 | 4.364105 |
| rs12590642 | -0.0446 | 0.495941 | 0.928343 | -1.01664 | 0.927444 | 0.95638 | 0.361807 | 2.528039 |
| rs12608932 | 0.575541 | 0.471531 | 0.222246 | -0.34866 | 1.499742 | 1.778092 | 0.705633 | 4.480533 |
| rs12960562 | 0.150778 | 0.440837 | 0.732331 | -0.71326 | 1.014818 | 1.162738 | 0.490043 | 2.758861 |
| rs138231518 | -0.12691 | 0.466283 | 0.785484 | -1.04083 | 0.787001 | 0.88081 | 0.353162 | 2.196799 |
| rs145178398 | -0.34226 | 0.560998 | 0.541804 | -1.44181 | 0.757298 | 0.710165 | 0.236499 | 2.132506 |
| rs148997216 | 0.077945 | 0.411231 | 0.849669 | -0.72807 | 0.883957 | 1.081063 | 0.482841 | 2.420459 |
| rs151233 | 0.733617 | 0.448709 | 0.10206 | -0.14585 | 1.613088 | 2.082601 | 0.864285 | 5.018284 |
| rs1574285 | 0.094091 | 0.444314 | 0.832288 | -0.77676 | 0.964946 | 1.09866 | 0.459892 | 2.624646 |
| rs1977833 | 0.170116 | 0.422551 | 0.687248 | -0.65808 | 0.998317 | 1.185442 | 0.517842 | 2.71371 |
| rs2037595 | 0.788104 | 0.414849 | 0.057468 | -0.025 | 1.601208 | 2.199223 | 0.97531 | 4.959021 |
| rs2101873 | 0.856484 | 0.479394 | 0.074003 | -0.08313 | 1.796097 | 2.354866 | 0.920232 | 6.026079 |
| rs2237897 | -0.95066 | 0.450539 | 0.034853 | -1.83372 | -0.06761 | 0.386484 | 0.159818 | 0.934626 |
| rs2284178 | -0.0437 | 0.132599 | 0.741729 | -0.30359 | 0.216194 | 0.957241 | 0.738161 | 1.241343 |
| rs2476601 | -0.24381 | 0.125111 | 0.051324 | -0.48903 | 0.001407 | 0.783635 | 0.613221 | 1.001408 |
| rs2797304 | 0.07303 | 0.451748 | 0.871572 | -0.8124 | 0.958457 | 1.075763 | 0.443793 | 2.60767 |
| rs281379 | -0.23054 | 0.462043 | 0.617812 | -1.13614 | 0.675066 | 0.794106 | 0.321055 | 1.964162 |
| rs2847278 | -0.11364 | 0.348337 | 0.744253 | -0.79638 | 0.569103 | 0.892582 | 0.45096 | 1.766682 |
| rs2961259 | -0.38951 | 0.472138 | 0.409374 | -1.3149 | 0.535879 | 0.677388 | 0.268501 | 1.70895 |
| rs3104414 | 0.137524 | 0.087246 | 0.114959 | -0.03348 | 0.308526 | 1.14743 | 0.967077 | 1.361416 |
| rs3184504 | -0.26565 | 0.270882 | 0.326751 | -0.79658 | 0.265281 | 0.766709 | 0.45087 | 1.303797 |
| rs35151229 | -0.23364 | 0.586607 | 0.690413 | -1.38339 | 0.916108 | 0.791645 | 0.250727 | 2.499542 |
| rs3818287 | 0.299746 | 0.400917 | 0.45467 | -0.48605 | 1.085543 | 1.349516 | 0.615051 | 2.961046 |
| rs3842753 | 0.024674 | 0.152554 | 0.87151 | -0.27433 | 0.323681 | 1.024981 | 0.76008 | 1.382206 |
| rs406767 | 0.097664 | 0.372574 | 0.79322 | -0.63258 | 0.827908 | 1.102592 | 0.531219 | 2.288527 |
| rs4682706 | -0.46175 | 0.505876 | 0.361362 | -1.45327 | 0.529767 | 0.63018 | 0.233805 | 1.698537 |
| rs56994090 | -0.3641 | 0.464461 | 0.433093 | -1.27444 | 0.546248 | 0.694825 | 0.279588 | 1.726763 |
| rs57052773 | -0.16233 | 0.490155 | 0.740503 | -1.12304 | 0.798371 | 0.850158 | 0.32529 | 2.221919 |
| rs574390686 | 0.403163 | 0.384284 | 0.29412 | -0.35003 | 1.15636 | 1.49655 | 0.704664 | 3.178342 |
| rs61775255 | 0.264946 | 0.456817 | 0.561926 | -0.63042 | 1.160308 | 1.303361 | 0.532371 | 3.190915 |
| rs61897928 | 0.317349 | 0.690423 | 0.645771 | -1.03588 | 1.670578 | 1.373482 | 0.354914 | 5.315237 |
| rs62021879 | -0.1194 | 0.442379 | 0.787238 | -0.98646 | 0.747665 | 0.887455 | 0.372894 | 2.112063 |
| rs706779 | 0.137454 | 0.408898 | 0.736753 | -0.66399 | 0.938894 | 1.147349 | 0.514795 | 2.557152 |
| rs7167984 | 0.068671 | 0.420948 | 0.870413 | -0.75639 | 0.89373 | 1.071084 | 0.469359 | 2.44423 |

| | | | | | | | | |
|---|---|---|---|---|---|---|---|---|
| rs72805613 | -0.17692 | 0.396961 | 0.655831 | -0.95496 | 0.601128 | 0.83785 | 0.384827 | 1.824175 |
| rs74203920 | -0.3635 | 0.371436 | 0.327756 | -1.09152 | 0.364511 | 0.695236 | 0.335706 | 1.43981 |
| rs74463708 | 0.296054 | 0.294295 | 0.314426 | -0.28076 | 0.872872 | 1.344543 | 0.755207 | 2.393776 |
| rs74759001 | -0.24978 | 0.348956 | 0.474122 | -0.93373 | 0.434176 | 0.778973 | 0.393084 | 1.54369 |
| rs74912105 | -0.15334 | 0.417749 | 0.71357 | -0.97213 | 0.665446 | 0.857837 | 0.378277 | 1.945359 |
| rs7501939 | -0.13495 | 0.503908 | 0.788842 | -1.12261 | 0.852706 | 0.873756 | 0.325428 | 2.345987 |
| rs7567242 | 0.034658 | 0.386994 | 0.928639 | -0.72385 | 0.793166 | 1.035266 | 0.484882 | 2.210383 |
| rs76072048 | -0.48103 | 0.449871 | 0.284951 | -1.36278 | 0.400717 | 0.618146 | 0.255949 | 1.492895 |
| rs76310611 | 0.195558 | 0.259225 | 0.450614 | -0.31252 | 0.703639 | 1.215989 | 0.731598 | 2.021095 |
| rs78221253 | 0.47169 | 0.500182 | 0.345662 | -0.50867 | 1.452048 | 1.602701 | 0.601296 | 4.271854 |
| rs7903146 | -0.16132 | 0.285677 | 0.572277 | -0.72125 | 0.398605 | 0.851018 | 0.486144 | 1.489745 |
| rs9273364 | 0.021291 | 0.05863 | 0.716498 | -0.09362 | 0.136207 | 1.02152 | 0.910625 | 1.145919 |
| rs9398803 | 0.021612 | 0.438167 | 0.960661 | -0.83719 | 0.880419 | 1.021847 | 0.432923 | 2.41191 |
| rs955033 | 0.13709 | 0.428941 | 0.749271 | -0.70363 | 0.977814 | 1.146931 | 0.494784 | 2.658639 |
| All - Inverse variance weighted | 0.013625 | 0.032529 | 0.67531 | -0.05013 | 0.077382 | 1.013719 | 0.951105 | 1.080454 |
| All - MR Egger | 0.021396 | 0.049616 | 0.667896 | -0.07585 | 0.118643 | 1.021627 | 0.926955 | 1.125967 |
