# Supplementary material for: No evidence of genetic causality between diabetes and osteonecrosis: a bidirectional two-sample Mendelian randomization analysis
Source: J Orthop Surg Res. 2023 Dec 16;18:970. doi: 10.1186/s13018-023-04428-7 (PMC10725608; doi:10.1186/s13018-023-04428-7)
Supplement: Supplementary file 3 — Additional file 3. Reverse MR instrumental variables. [file 13018_2023_4428_MOESM3_ESM.pdf]

Reverse-MR instrumental variables

Table 1: ebi-a-GCST006867

SNP	b	se	p	lo_ci	up_ci	or	or_lci95	or_uci95
rs245878	-0.00948	0.046284	0.837713	-0.1002	0.081237	0.990565	0.904659	1.084628
rs35498319	-0.05334	0.041881	0.202762	-0.13543	0.028742	0.948053	0.873338	1.029159
rs4945708	-0.00457	0.049239	0.926076	-0.10108	0.09194	0.995442	0.903863	1.096299
rs60834670	0.099319	0.048491	0.040541	0.004277	0.194362	1.104419	1.004286	1.214536
rs6482921	-0.03834	0.046009	0.404657	-0.12852	0.051837	0.962385	0.879396	1.053204
rs72766422	0.089876	0.043633	0.039415	0.004356	0.175397	1.094039	1.004365	1.191719
All - Inverse variance weighted	0.01205	0.026895	0.65412	-0.04066	0.064764	1.012123	0.960152	1.066908
All - MR Egger	-0.0566	0.08169	0.526488	-0.21672	0.103508	0.944968	0.805158	1.109054

Table 2: ebi-a-GCST005413

SNP	b	se	p	lo_ci	up_ci	or	or_lci95	or_uci95
rs11757625	-0.04945	0.091833	0.590258	-0.22944	0.130545	0.951754	0.794977	1.139449
rs117909311	0.06045	0.089095	0.49746	-0.11418	0.235077	1.062315	0.892101	1.265006
rs12444537	-0.11674	0.097853	0.232876	-0.30853	0.075055	0.889819	0.734527	1.077943
rs141188770	-0.02343	0.028783	0.415656	-0.07984	0.032986	0.976843	0.92326	1.033536
rs143477778	-0.10994	0.077262	0.154769	-0.26137	0.041499	0.895892	0.769997	1.042372
rs144456859	0.004892	0.081568	0.952175	-0.15498	0.164765	1.004904	0.856432	1.179116
rs148308717	-0.23391	0.165305	0.157054	-0.55791	0.090083	0.79143	0.572403	1.094265
rs245878	0.017844	0.092568	0.847138	-0.16359	0.199278	1.018005	0.849091	1.220522
rs35498319	-0.04815	0.085138	0.571714	-0.21502	0.118722	0.952993	0.806526	1.126057
rs4945708	-0.09543	0.098986	0.334995	-0.28944	0.09858	0.90898	0.748679	1.103602
rs60834670	0.039728	0.096398	0.680251	-0.14921	0.228668	1.040527	0.861386	1.256925
rs6482921	0.027797	0.09106	0.760166	-0.15068	0.206275	1.028187	0.860122	1.229092
rs72704886	0.087735	0.089323	0.32599	-0.08734	0.262807	1.091699	0.916367	1.300576
rs72766422	-0.0082	0.088758	0.926351	-0.18217	0.16576	0.991829	0.83346	1.18029
All - Inverse variance weighted	-0.02245	0.019077	0.239332	-0.05984	0.014944	0.977802	0.941916	1.015056
All - MR Egger	-0.02272	0.026877	0.414492	-0.0754	0.02996	0.977538	0.927375	1.030414

Table 3: GCST90006934

SNP	b	se	p	lo_ci	up_ci	or	or_lci95	or_uci95
rs11757625	0.054339	0.108135	0.615307	-0.15761	0.266284	1.055843	0.854187	1.305106
rs117909311	-0.05768	0.121098	0.633828	-0.29504	0.179668	0.943948	0.744504	1.19682
rs12444537	-0.22031	0.128754	0.087061	-0.47267	0.032045	0.802268	0.623336	1.032564
rs141000138	0.010756	0.074996	0.885956	-0.13624	0.157748	1.010814	0.872637	1.170871
rs141188770	-0.08816	0.043755	0.043925	-0.17392	-0.0024	0.915617	0.840367	0.997605
rs143477778	0.157856	0.099044	0.110983	-0.03627	0.351983	1.170997	0.964379	1.421884
rs144456859	0.067291	0.106827	0.528756	-0.14209	0.276672	1.069607	0.867543	1.318733

rs148308717	-0.04153	0.234717	0.859569	-0.50157	0.418518	0.959324	0.605578	1.519708
rs245878	-0.03569	0.117105	0.760547	-0.26521	0.193836	0.96494	0.767042	1.213897
rs35498319	0.016966	0.113263	0.880924	-0.20503	0.238961	1.017111	0.814624	1.269929
rs4945708	0.008122	0.128428	0.949575	-0.2436	0.25984	1.008155	0.783804	1.296723
rs60834670	-0.17585	0.122689	0.151763	-0.41632	0.064616	0.838741	0.659467	1.066749
rs6482921	-0.06183	0.115503	0.592463	-0.28821	0.16456	0.940047	0.749604	1.178875
rs72704886	-0.26817	0.107386	0.012517	-0.47864	-0.05769	0.76478	0.619624	0.943942
rs72766422	0.03692	0.113371	0.744683	-0.18529	0.259127	1.03761	0.830866	1.295799
All - Inverse variance weighted	-0.04451	0.026413	0.091959	-0.09628	0.007259	0.956467	0.908212	1.007286
All - MR Egger	-0.03606	0.039161	0.373862	-0.11282	0.040691	0.964578	0.893311	1.041531

Table 4: GCST90026414

SNP	b	se	p	lo_ci	up_ci	or	or_lci95	or_uci95
rs11757625	-0.30191	0.291008	0.299517	-0.87229	0.268464	0.739403	0.417994	1.307954
rs12444537	-0.27752	0.340208	0.414653	-0.94433	0.389289	0.757661	0.388941	1.475932
rs245878	-0.32915	0.304409	0.279569	-0.9258	0.267488	0.719532	0.396216	1.306678
rs4945708	-0.03369	0.339021	0.92083	-0.69818	0.630786	0.966867	0.497492	1.879087
rs60834670	0.522484	0.318962	0.101407	-0.10268	1.14765	1.68621	0.902413	3.150779
rs6482921	-0.35524	0.312715	0.25596	-0.96816	0.25768	0.701003	0.379779	1.293924
rs72766422	0.292227	0.295652	0.322949	-0.28725	0.871705	1.339407	0.750323	2.390984
All - Inverse variance weighted	-0.06946	0.132518	0.600169	-0.32919	0.190275	0.932897	0.719503	1.209582
All - MR Egger	0.49492	1.046563	0.656208	-1.55634	2.546183	1.640368	0.210906	12.75832

Table 5: GCST90026417

SNP	b	se	p	lo_ci	up_ci	or	or_lci95	or_uci95
rs11757625	-0.59287	0.176059	0.000759	-0.93795	-0.2478	0.552736	0.39143	0.780517
rs12444537	-0.30346	0.207971	0.14452	-0.71109	0.104159	0.738256	0.49111	1.109777
rs245878	-0.02633	0.187325	0.888225	-0.39349	0.340828	0.974015	0.674701	1.406111
rs4945708	-0.16201	0.205651	0.430825	-0.56508	0.241068	0.850435	0.568313	1.272607
rs60834670	0.370139	0.194841	0.057473	-0.01175	0.752028	1.447936	0.988319	2.121298
rs6482921	0.146988	0.185949	0.42925	-0.21747	0.511448	1.15834	0.80455	1.667704
rs72766422	-0.02421	0.183947	0.895273	-0.38475	0.336322	0.976077	0.680621	1.39979
All - Inverse variance weighted	-0.09067	0.12058	0.452102	-0.327	0.145671	0.913323	0.721081	1.156816
All - MR Egger	0.09791	0.990452	0.925095	-1.84338	2.039197	1.102863	0.158282	7.684433

Table 6: GCST90043636

SNP	b	se	p	lo_ci	up_ci	or	or_lci95	or_uci95
rs11757625	1.117579	0.840139	0.183442	-0.52909	2.764252	3.057444	0.589139	15.86717
rs117909311	-0.56918	0.85096	0.503581	-2.23706	1.098703	0.56599	0.106772	3.000273
rs12444537	-0.97412	0.924264	0.291911	-2.78568	0.83744	0.377525	0.061687	2.310445
rs141188770	0.0849	0.298126	0.775814	-0.49943	0.669226	1.088608	0.606879	1.952725

rs143477778	-0.38664	0.769602	0.615398	-1.89506	1.121785	0.679339	0.15031	3.07033
rs144456859	1.256445	0.868163	0.147828	-0.44515	2.958043	3.512909	0.640726	19.26025
rs148308717	0.077369	1.444768	0.957293	-2.75438	2.909115	1.08044	0.063649	18.34055
rs245878	-0.53666	0.908982	0.554926	-2.31826	1.244946	0.584699	0.098444	3.472747
rs35498319	0.266827	0.812537	0.742618	-1.32575	1.859401	1.305815	0.265605	6.419889
rs4945708	-0.35408	0.97935	0.717694	-2.2736	1.565449	0.701821	0.102941	4.784825
rs60834670	0.290748	0.950919	0.759791	-1.57305	2.154549	1.337428	0.207411	8.623998
rs6482921	0.659847	0.909251	0.468021	-1.12229	2.441979	1.934496	0.325535	11.49577
rs72704886	0.177949	0.789824	0.821744	-1.37011	1.726003	1.194764	0.25408	5.618154
rs72766422	0.28003	0.860505	0.744859	-1.40656	1.96662	1.323169	0.244985	7.14648
All - Inverse variance weighted	0.102058	0.18983	0.590835	-0.27001	0.474125	1.107448	0.763372	1.606608
All - MR Egger	0.130685	0.273973	0.64193	-0.4063	0.667672	1.139609	0.666109	1.949694

Table 7: ebi-a-GCST010681

SNP	b	se	p	lo_ci	up_ci	or	or_lci95	or_uci95
rs11757625	0.21627	0.133675	0.105687	-0.04573	0.478272	1.241438	0.955298	1.613285
rs117909311	0.080008	0.127222	0.529426	-0.16935	0.329363	1.083295	0.844215	1.390083
rs12444537	-0.11044	0.15336	0.471433	-0.41103	0.190144	0.895438	0.662968	1.209423
rs141000138	0.053533	0.065677	0.415018	-0.07519	0.18226	1.054992	0.927564	1.199926
rs141188770	-0.15125	0.041981	0.000315	-0.23353	-0.06897	0.859634	0.791732	0.933359
rs143477778	-0.17784	0.111857	0.111853	-0.39708	0.041396	0.837073	0.672277	1.042265
rs144456859	-0.02716	0.128092	0.832105	-0.27822	0.223905	0.973209	0.757132	1.250953
rs148308717	0.313156	0.211847	0.139349	-0.10206	0.728376	1.367735	0.902972	2.071714
rs245878	0.007807	0.140525	0.955696	-0.26762	0.283237	1.007838	0.765196	1.327419
rs35498319	-0.12136	0.125338	0.332898	-0.36703	0.124298	0.885712	0.692792	1.132354
rs4945708	-0.18731	0.148732	0.207891	-0.47883	0.104204	0.829185	0.61951	1.109827
rs60834670	0.021032	0.144305	0.88412	-0.26181	0.303871	1.021255	0.76966	1.355094
rs6482921	-0.10496	0.13659	0.442237	-0.37268	0.162758	0.900361	0.688888	1.176752
rs72704886	0.041485	0.119891	0.729323	-0.1935	0.276472	1.042358	0.824069	1.31847
rs72766422	0.073467	0.131645	0.576794	-0.18456	0.331491	1.076233	0.831473	1.393044
All - Inverse variance weighted	-0.05509	0.031446	0.079817	-0.11672	0.006549	0.946405	0.889835	1.00657
All - MR Egger	-0.09078	0.041624	0.048153	-0.17236	-0.0092	0.913219	0.841674	0.990846

Table 8: ebi-a-GCST90000529

SNP	b	se	p	lo_ci	up_ci	or	or_lci95	or_uci95
rs11757625	0.316258	0.123109	0.010201	0.074965	0.557552	1.371985	1.077846	1.746392
rs117909311	-0.25942	0.139634	0.06319	-0.5331	0.014263	0.7715	0.586783	1.014365
rs12444537	0.021516	0.147481	0.884011	-0.26755	0.310578	1.021749	0.765254	1.364214
rs143477778	-0.20767	0.124125	0.094308	-0.45096	0.035612	0.812472	0.637017	1.036253
rs148308717	0.131168	0.21227	0.536622	-0.28488	0.547218	1.140159	0.752103	1.728437
rs245878	-0.09476	0.136262	0.486775	-0.36184	0.172311	0.909588	0.696396	1.188047
rs35498319	-0.1044	0.126627	0.40966	-0.35259	0.143786	0.900862	0.702864	1.154637
rs4945708	0.06301	0.138492	0.64913	-0.20843	0.334454	1.065037	0.811855	1.397177
rs60834670	0.270198	0.13462	0.044737	0.006344	0.534053	1.310224	1.006364	1.705832

rs6482921	0.104518	0.126407	0.408327	-0.14324	0.352275	1.110175	0.866547	1.422299
rs72704886	0.045209	0.115359	0.695134	-0.1809	0.271313	1.046247	0.834523	1.311686
rs72766422	-0.1555	0.122256	0.203395	-0.39512	0.084119	0.855985	0.673597	1.087759
All - Inverse variance weighted	0.006039	0.053148	0.90954	-0.09813	0.110208	1.006057	0.90653	1.116511
All - MR Egger	-0.22478	0.102374	0.052828	-0.42543	-0.02413	0.798693	0.653487	0.976163

Table 9: ebi-a-GCST90014023

SNP	b	se	p	lo_ci	up_ci	or	or_lci95	or_uci95
rs11757625	0.372099	0.078335	2.03E-06	0.218561	0.525637	1.450777	1.244285	1.691535
rs117909311	-0.01767	0.081274	0.82786	-0.17697	0.141624	0.982482	0.837805	1.152144
rs12444537	-0.10252	0.094917	0.280087	-0.28856	0.083516	0.902558	0.749341	1.087102
rs141000138	0.011116	0.04822	0.817681	-0.08339	0.105627	1.011178	0.919988	1.111407
rs141188770	-0.02163	0.027755	0.435878	-0.07603	0.032774	0.978606	0.926792	1.033317
rs143477778	-0.13934	0.072888	0.055912	-0.2822	0.003519	0.869931	0.754122	1.003525
rs144456859	-0.00778	0.081564	0.924034	-0.16764	0.152088	0.992253	0.845656	1.164262
rs148308717	-0.02035	0.143859	0.887508	-0.30231	0.261614	0.979856	0.739106	1.299025
rs245878	-0.08664	0.090483	0.338296	-0.26399	0.090706	0.917007	0.767984	1.094947
rs35498319	-0.01178	0.080302	0.883339	-0.16917	0.145608	0.988286	0.844361	1.156743
rs4945708	0.071387	0.096402	0.458991	-0.11756	0.260334	1.073996	0.889086	1.297364
rs60834670	0.102223	0.088383	0.247437	-0.07101	0.275453	1.10763	0.931456	1.317127
rs6482921	-0.05173	0.089306	0.562411	-0.22677	0.123308	0.949583	0.797103	1.131233
rs72704886	0.044778	0.072351	0.535981	-0.09703	0.186587	1.045796	0.907528	1.20513
rs72766422	-0.07986	0.08724	0.359981	-0.25085	0.091131	0.923246	0.77814	1.095412
All - Inverse variance weighted	-0.00147	0.025748	0.954373	-0.05194	0.048993	0.998528	0.949387	1.050213
All - MR Egger	-0.03183	0.034685	0.375554	-0.09981	0.036157	0.968675	0.90501	1.036819

Table 10: ebi-a-GCST90018925

SNP	b	se	p	lo_ci	up_ci	or	or_lci95	or_uci95
rs11757625	-0.05162	0.096724	0.593544	-0.2412	0.137957	0.949687	0.785684	1.147926
rs117909311	-0.03319	0.111418	0.765801	-0.25157	0.185191	0.967356	0.777581	1.203449
rs12444537	0.060657	0.110442	0.582853	-0.15581	0.277124	1.062535	0.855722	1.31933
rs141000138	0.092543	0.308905	0.764495	-0.51291	0.697996	1.09696	0.59875	2.009721
rs141188770	0.015551	0.037787	0.680668	-0.05851	0.089613	1.015673	0.943168	1.093751
rs143477778	0.110192	0.105195	0.294868	-0.09599	0.316373	1.116492	0.908473	1.372142
rs144456859	0.150856	0.124099	0.224135	-0.09238	0.39409	1.162829	0.91176	1.483033
rs148308717	-0.10271	0.137219	0.454136	-0.37166	0.166236	0.902385	0.689587	1.180851
rs245878	-0.01729	0.098702	0.860969	-0.21074	0.17617	0.982862	0.809982	1.192641
rs35498319	0.03378	0.091099	0.710783	-0.14477	0.212334	1.034357	0.865218	1.236561
rs4945708	0.114214	0.102539	0.265339	-0.08676	0.315191	1.120992	0.916895	1.370521
rs60834670	0.128531	0.10633	0.226743	-0.07988	0.336938	1.137157	0.923231	1.400652
rs6482921	-0.00335	0.108793	0.9754	-0.21659	0.20988	0.996651	0.805261	1.233529
rs72704886	-0.11493	0.087933	0.191213	-0.28728	0.057421	0.89143	0.750303	1.059101
rs72766422	-0.00224	0.110015	0.983773	-0.21787	0.213391	0.997765	0.804233	1.237869

All - Inverse variance weighted	0.016987	0.022991	0.459999	-0.02808	0.06205	1.017132	0.972315	1.064015
All - MR Egger	0.011688	0.034232	0.738233	-0.05541	0.078782	1.011756	0.946101	1.081968
