# Supplementary material for: No evidence of genetic causality between diabetes and osteonecrosis: a bidirectional two-sample Mendelian randomization analysis
Source: J Orthop Surg Res. 2023 Dec 16;18:970. doi: 10.1186/s13018-023-04428-7 (PMC10725608; doi:10.1186/s13018-023-04428-7)
Supplement: Supplementary file 4 — Additional file 4. Leave one out and funnel plots of the results of the r everse MR analyses of each data for T2DM and T1DM. [file 13018_2023_4428_MOESM4_ESM.pdf]

Reverse MR leave-one-out method and funnel plots

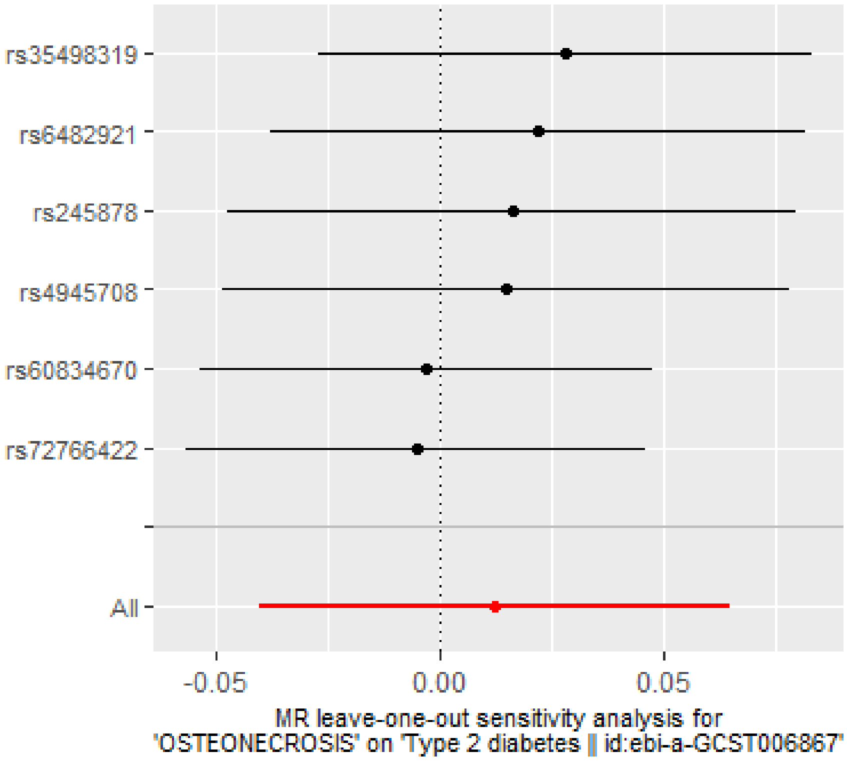

Figure 1 Leave-one-out method for ebi-a-GCST006867

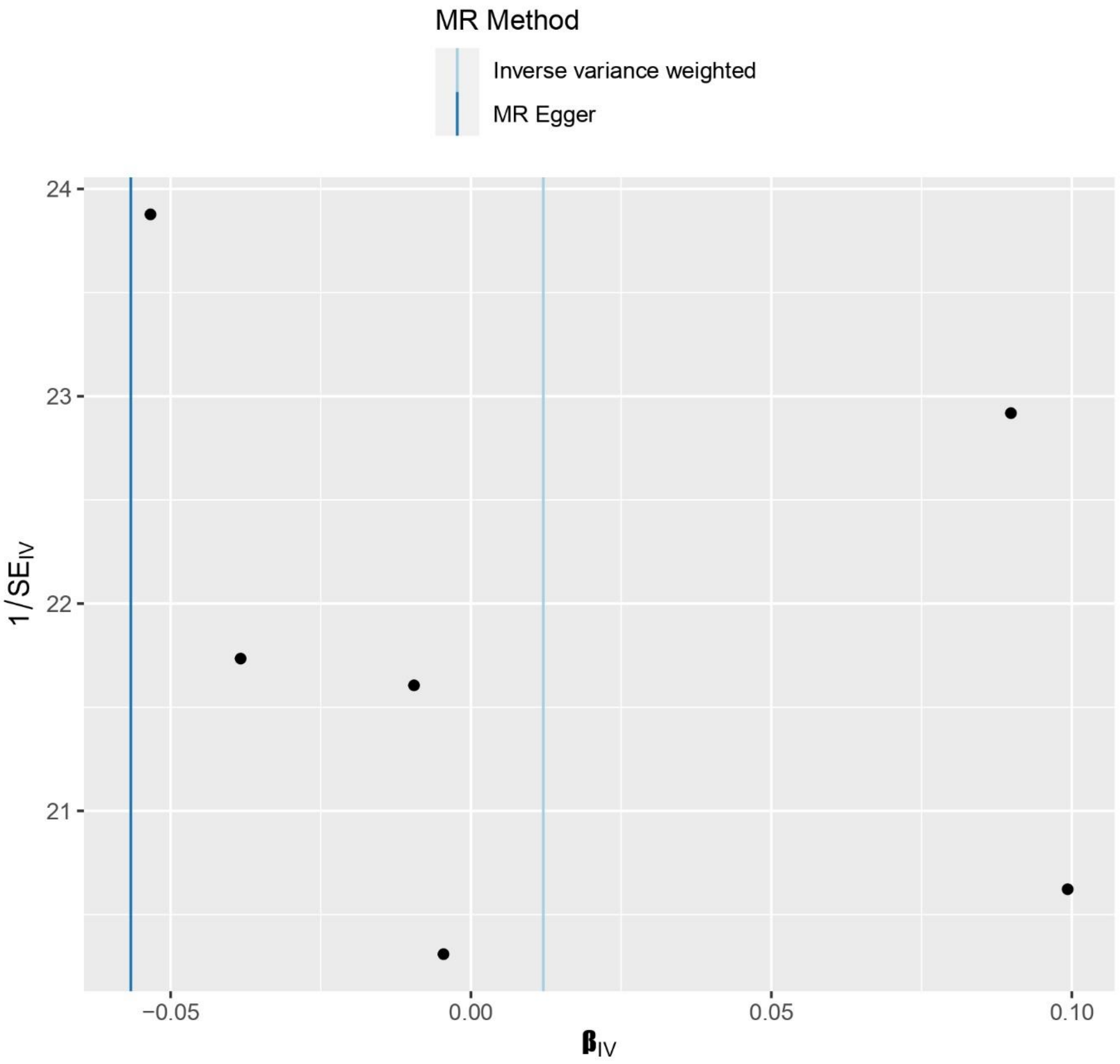

Figure 2 Funnel plot for ebi-a-GCST006867

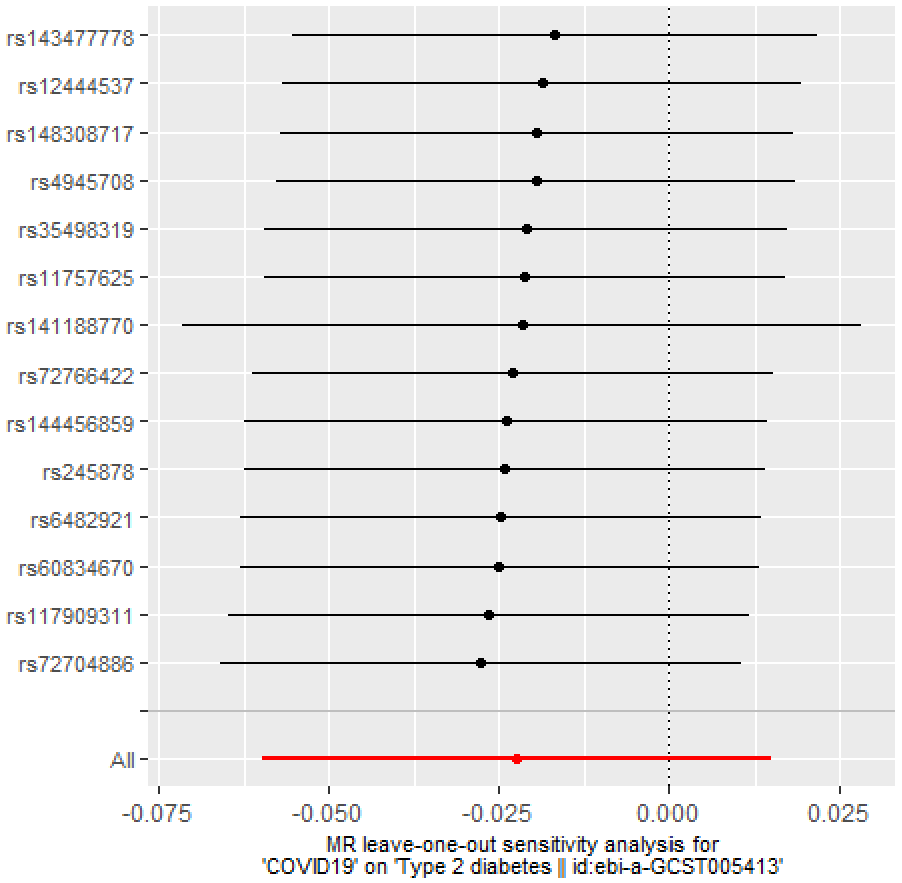

Figure 3 Leave-one-out method for ebi-a-GCST005413

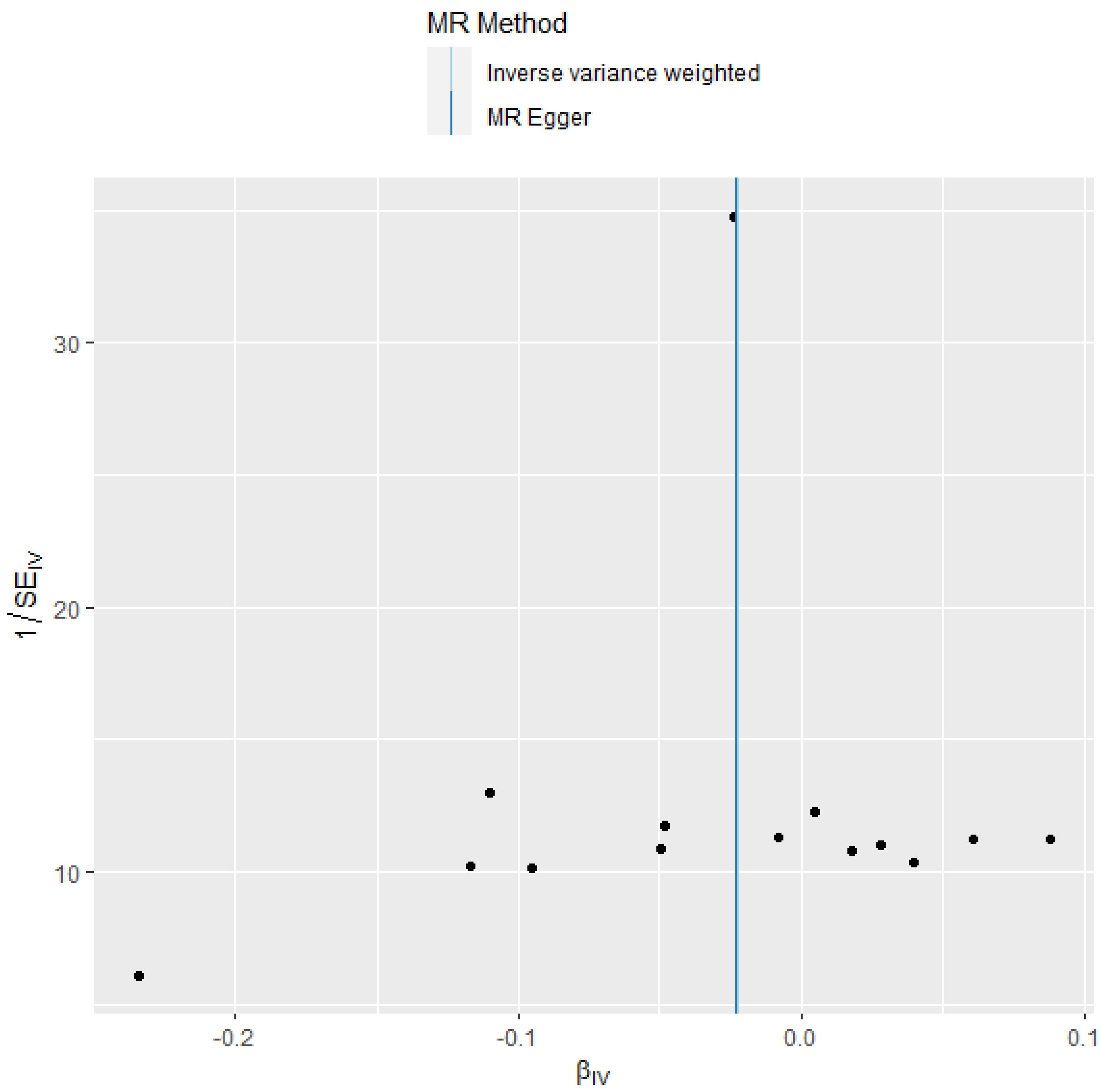

Figure 4 Funnel plot for ebi-a-GCST005413

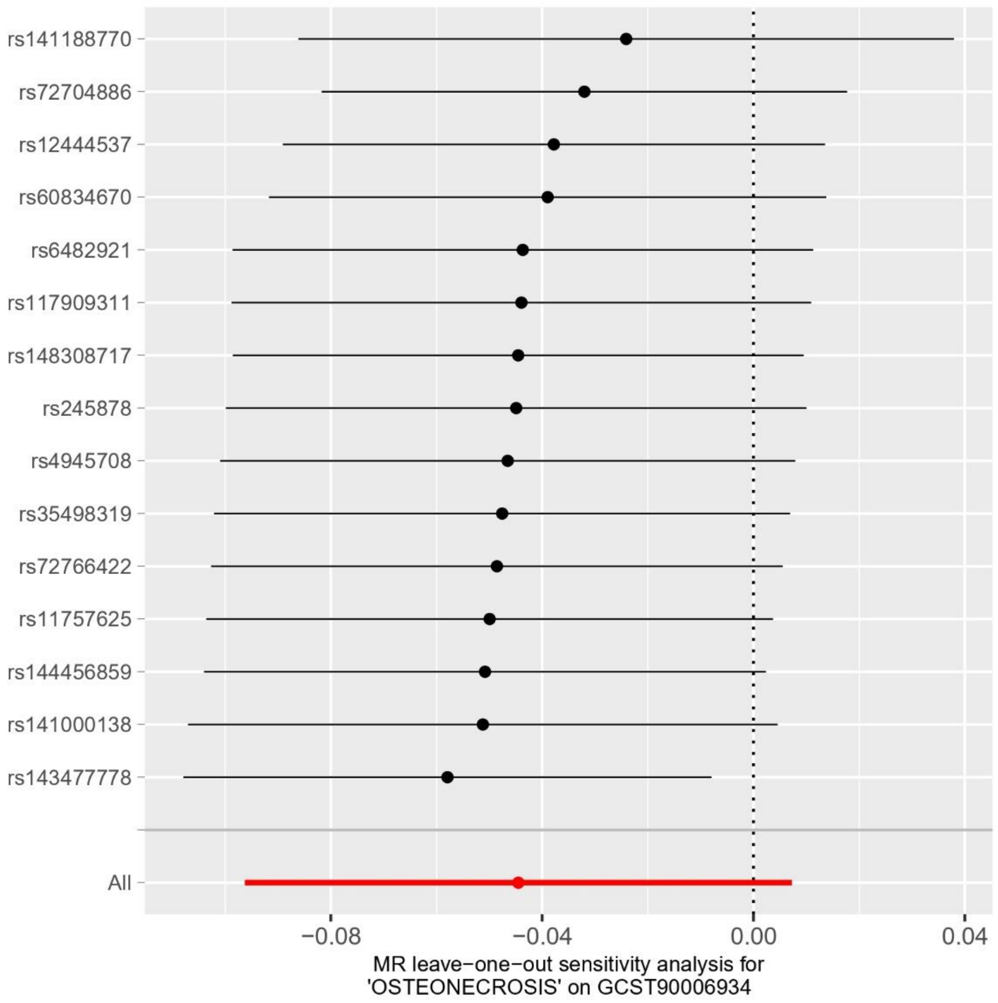

Figure 5 Leave-one-out method for GCST90006934

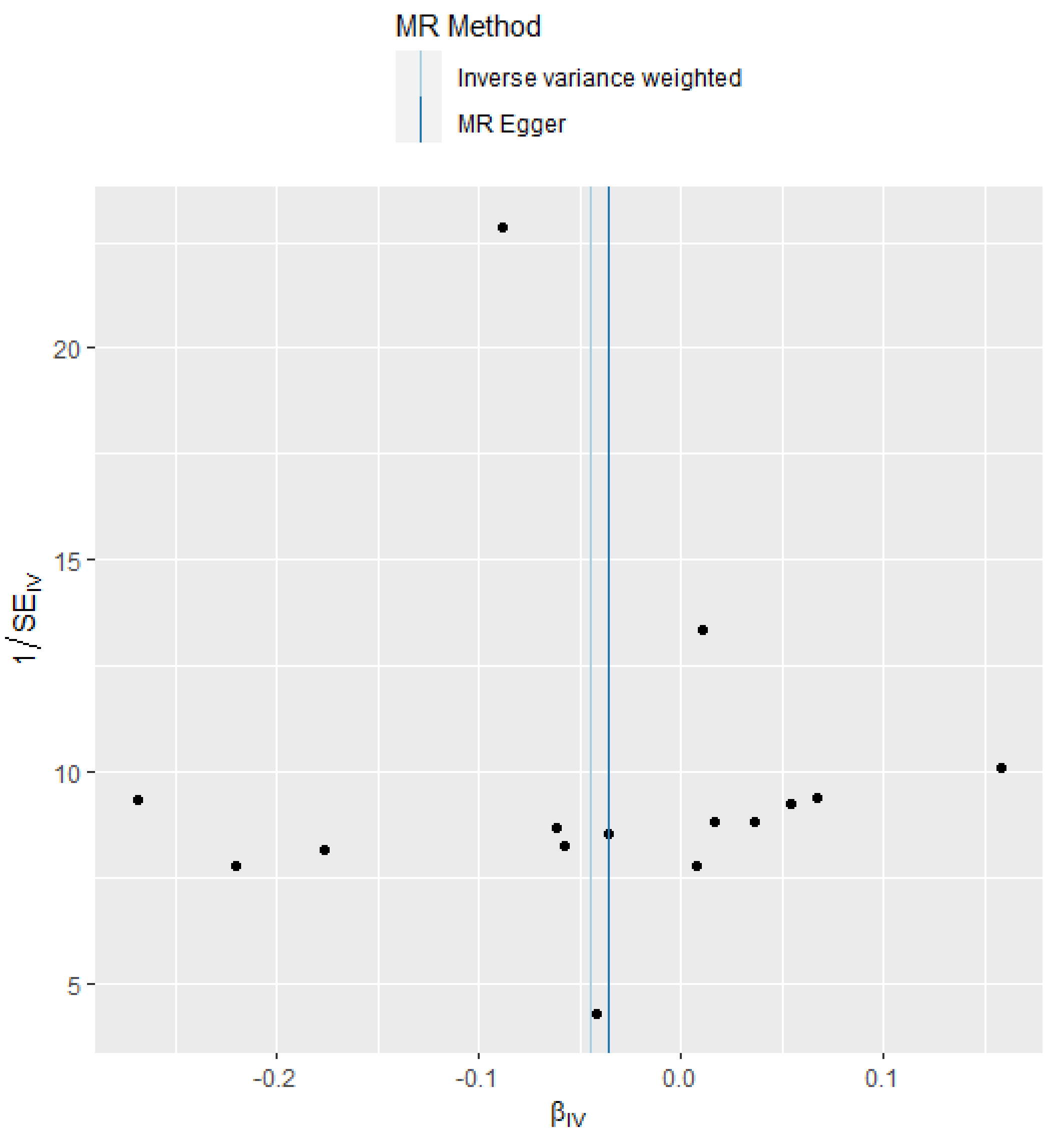

Figure 6 Funnel plot for GCST90006934

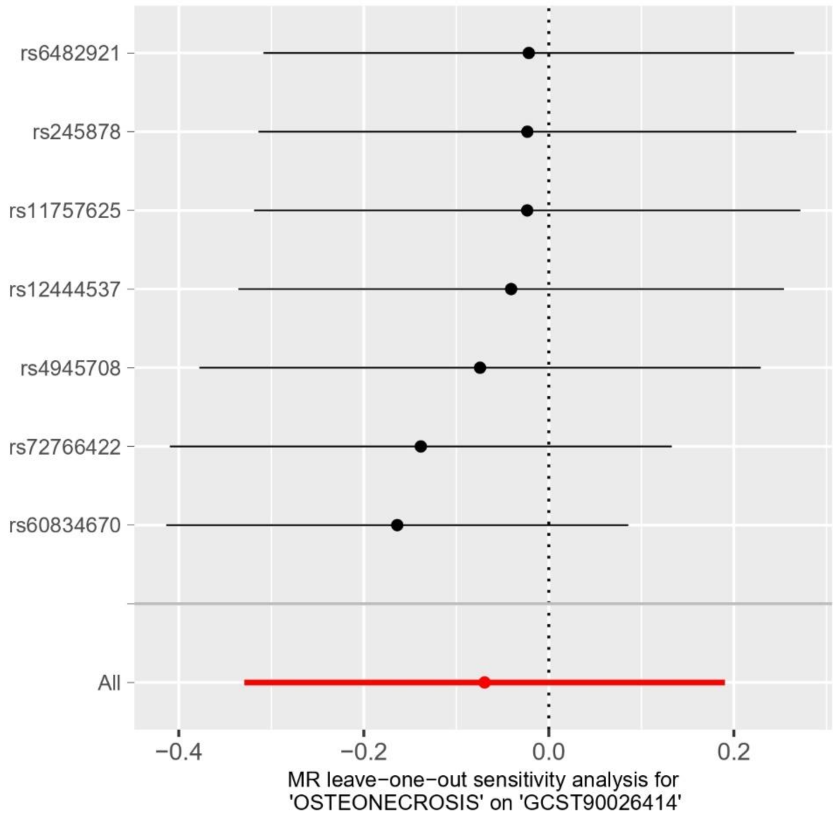

Figure 7 Leave-one-out method for GCST90026414

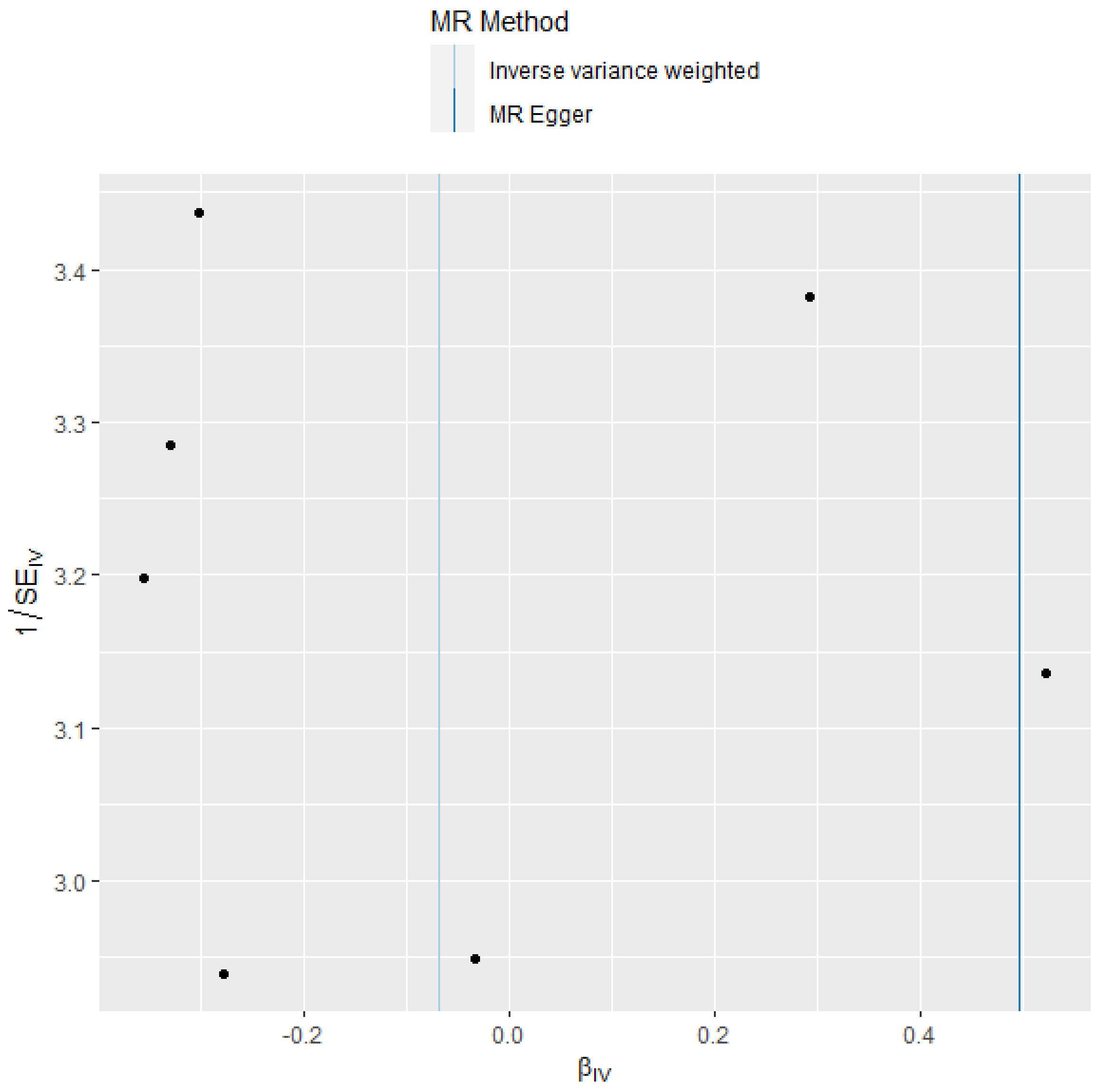

Figure 8 Funnel plot for GCST90026414

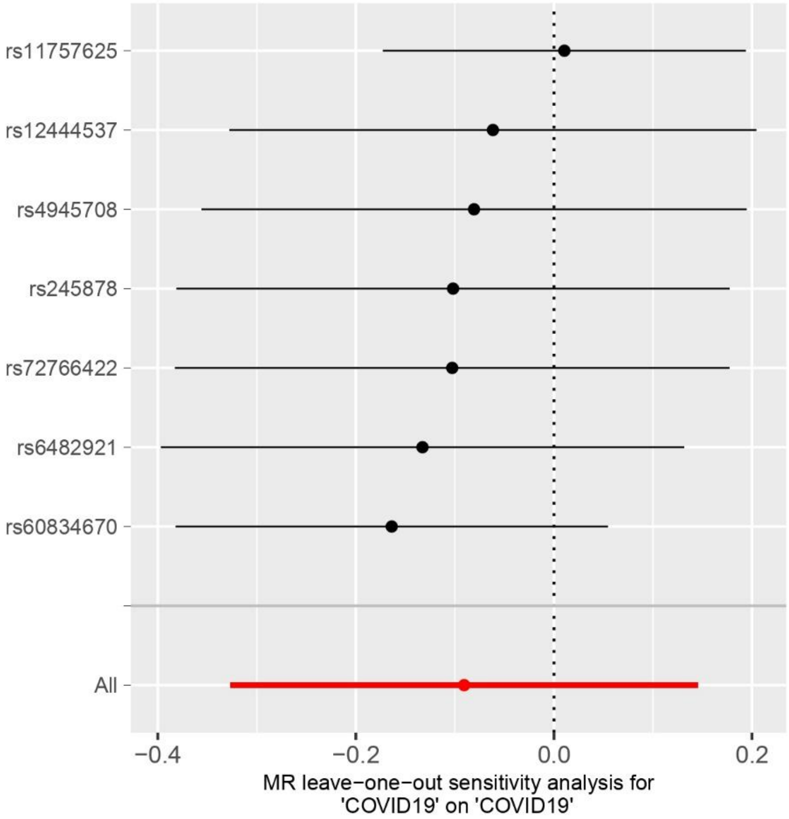

Figure 9 Leave-one-out method for GCST90026417

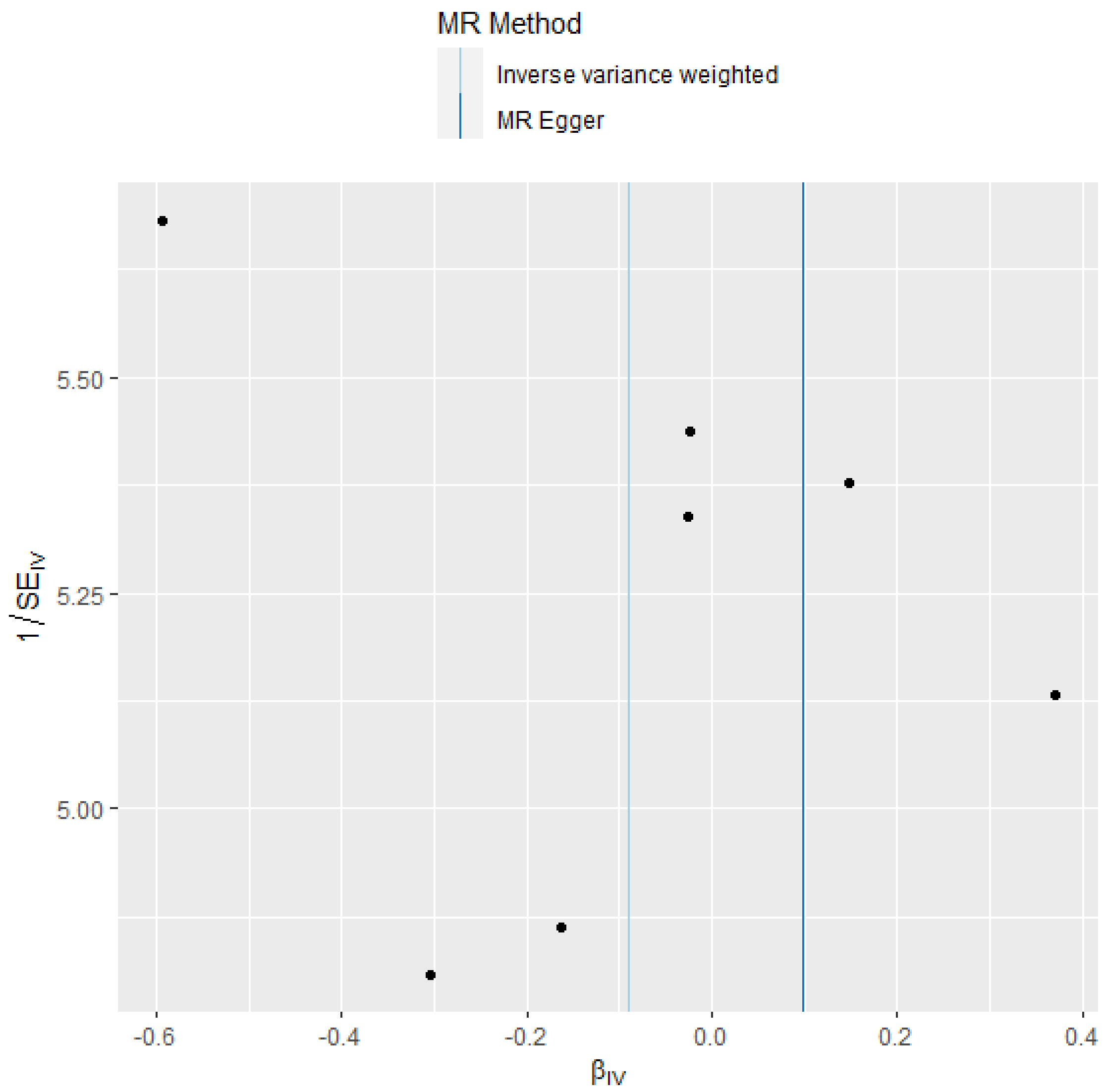

Figure 10 Funnel plot for GCST90026417

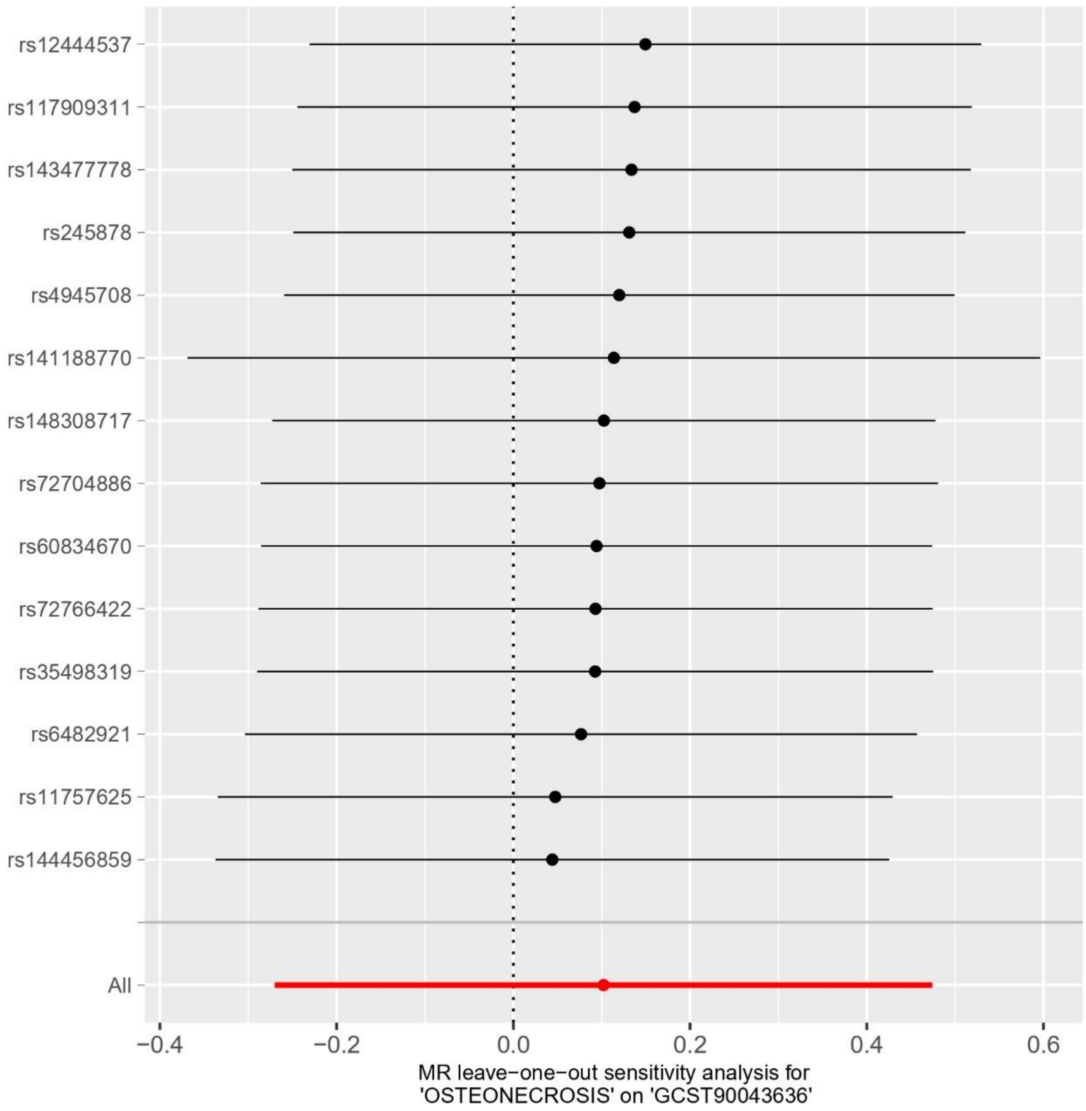

Figure 11 Leave-one-out method for GCST90043636

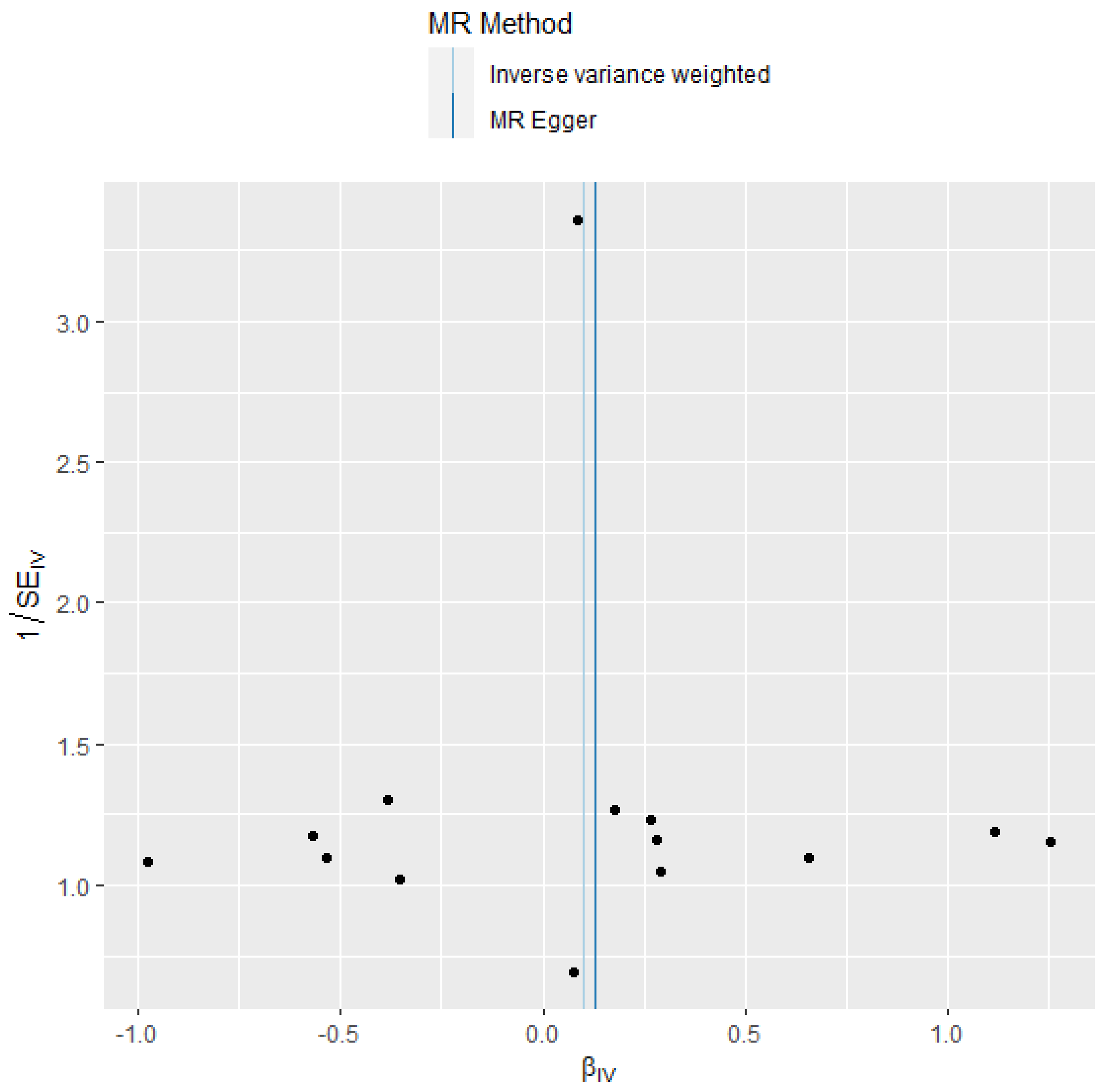

Figure 12 Funnel plot for GCST90043636

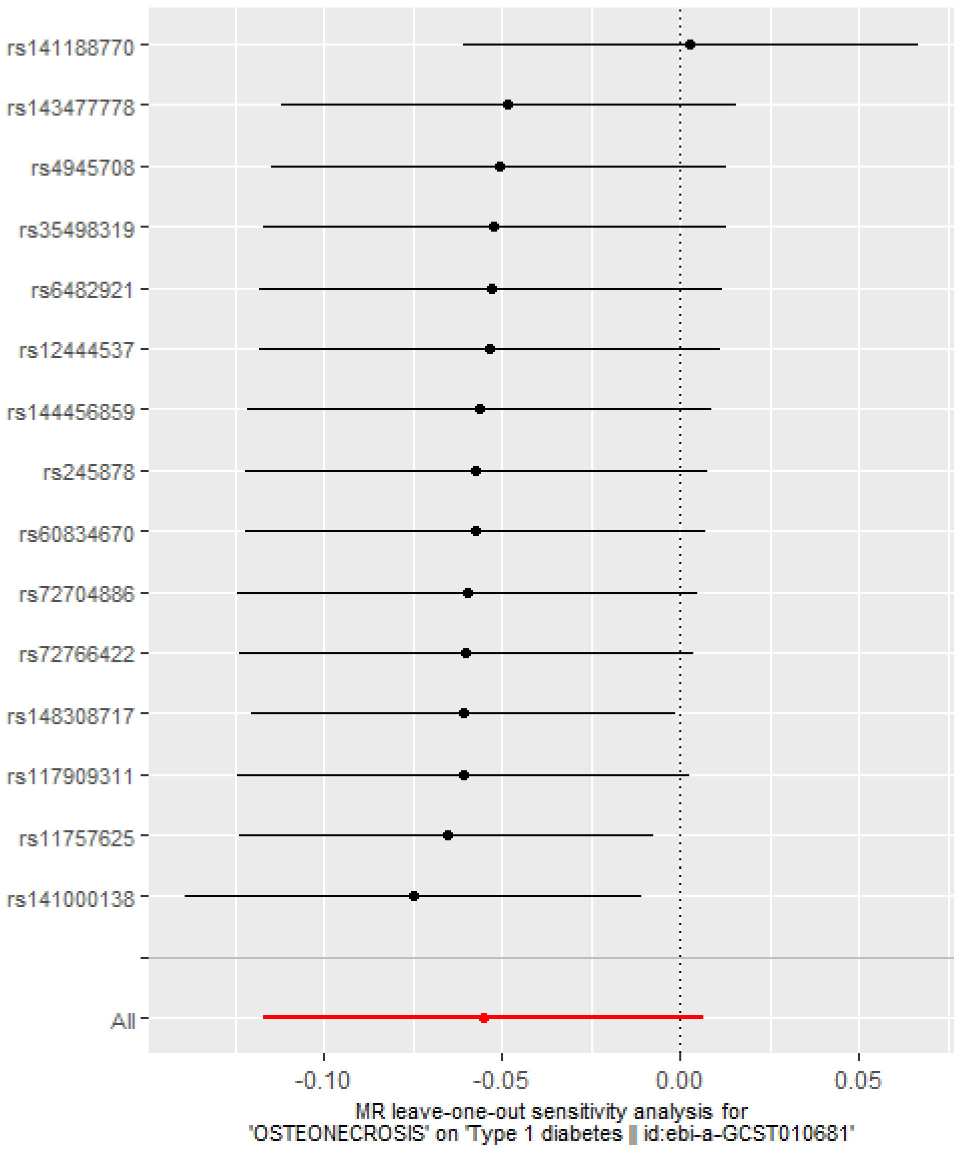

Figure 11 Leave-one-out method for ebi-a-GCST010681

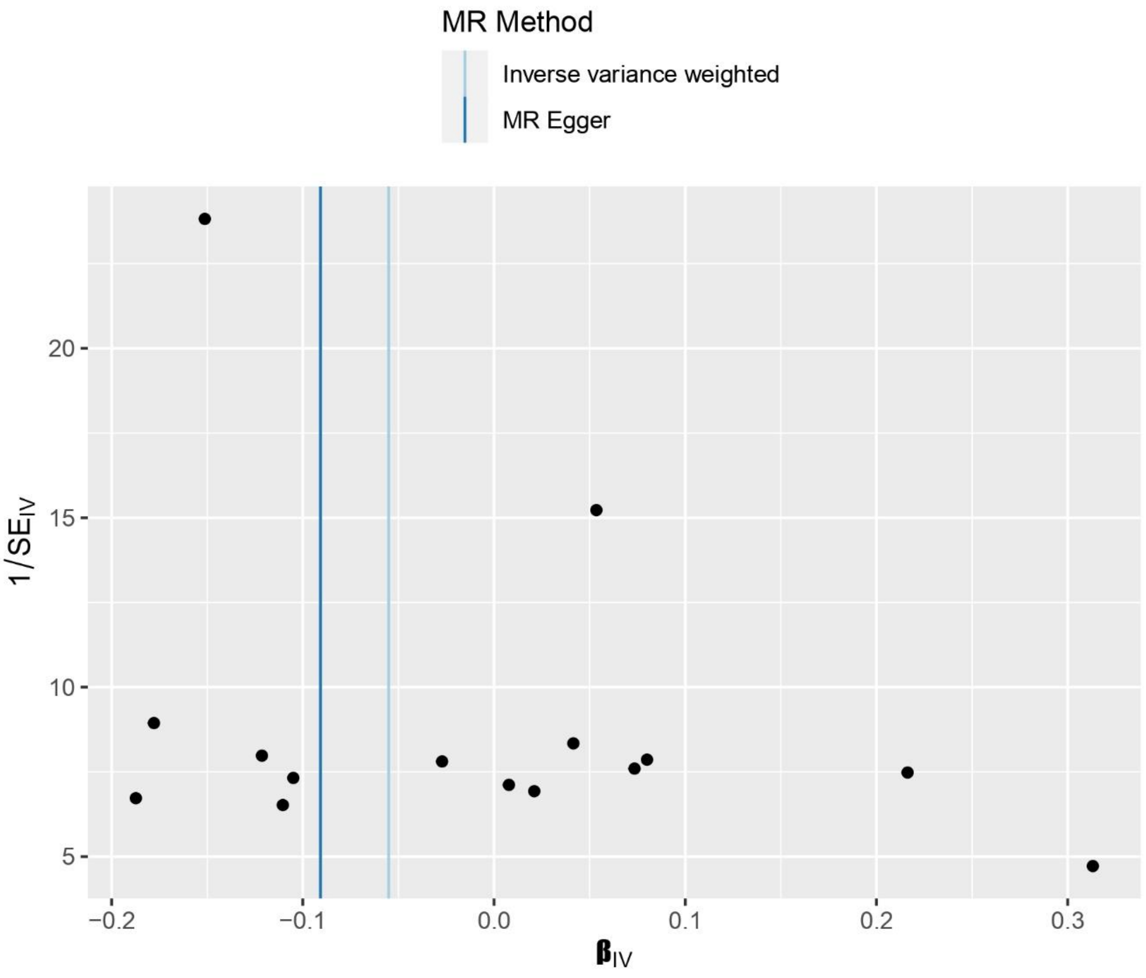

Figure 12 Funnel plot for ebi-a-GCST010681

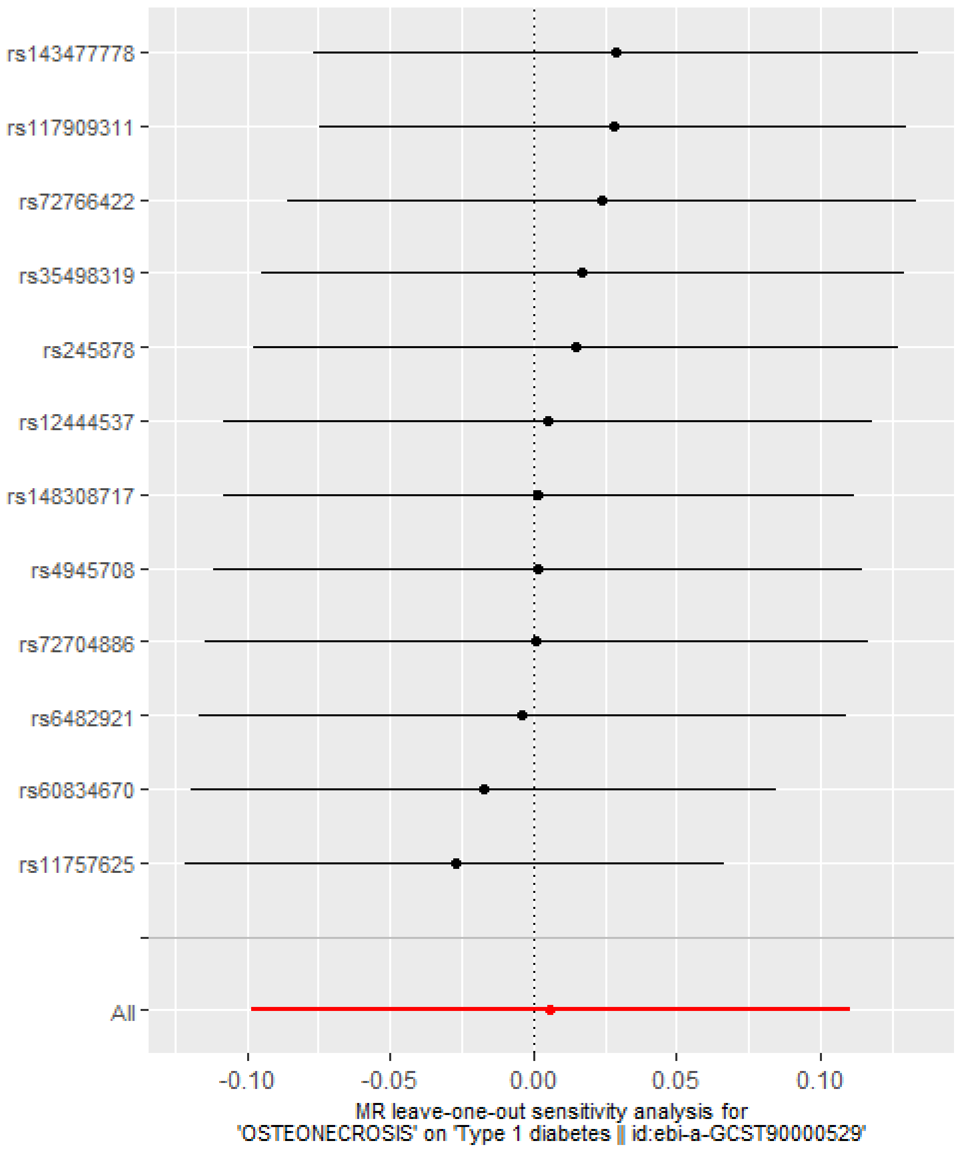

Figure 13 Leave-one-out method for ebi-a-GCST90000529

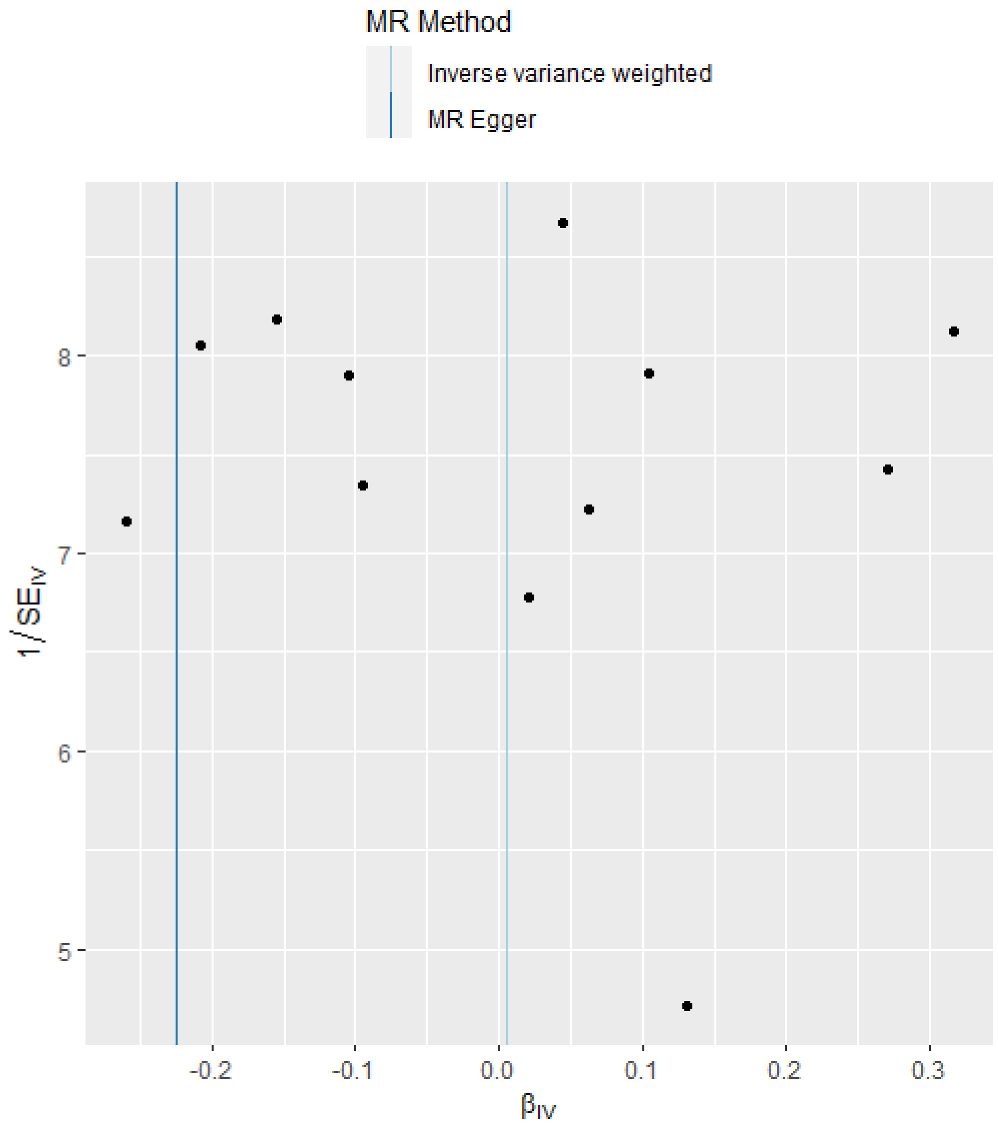

Figure 14 Funnel plot for ebi-a-GCST90000529

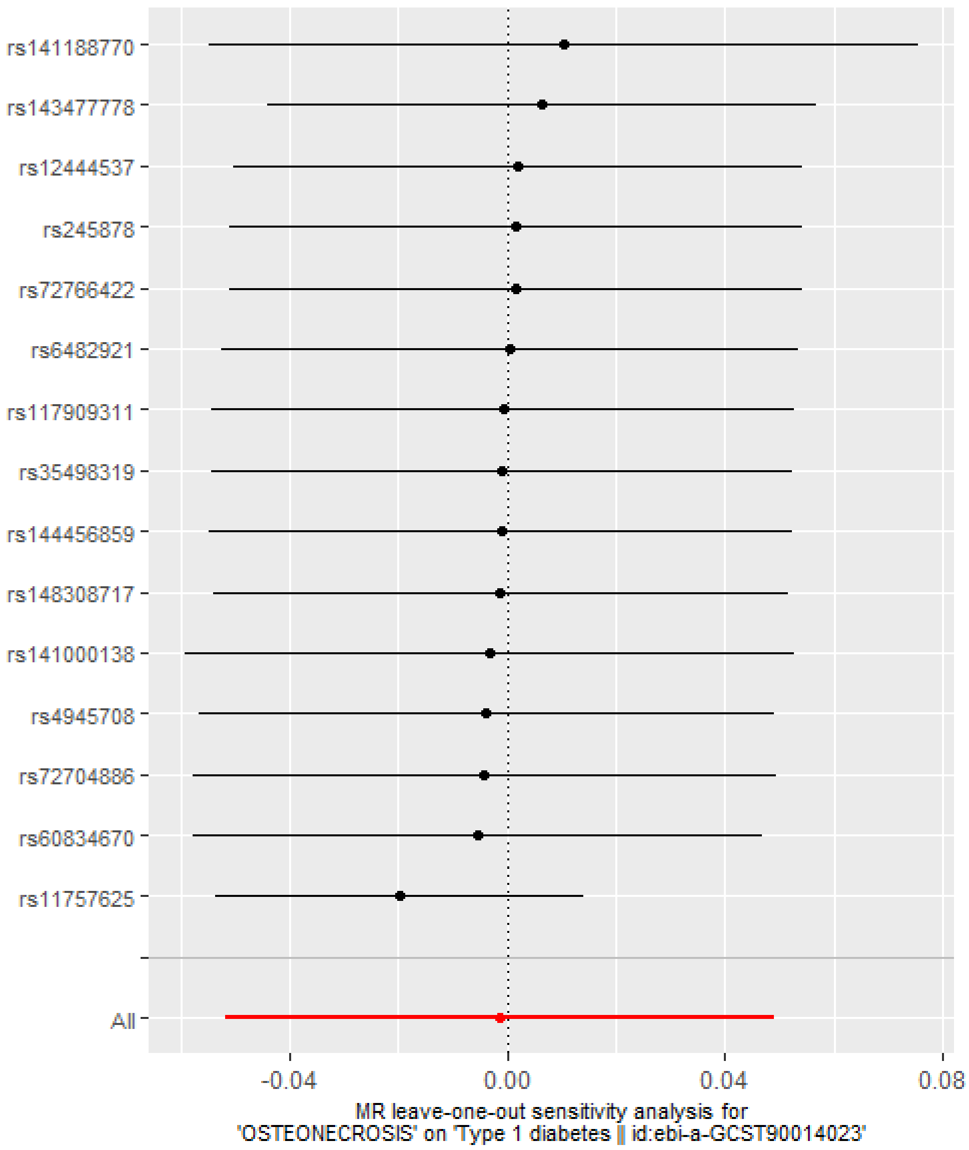

Figure 15 Leave-one-out method for ebi-a-GCST90014023

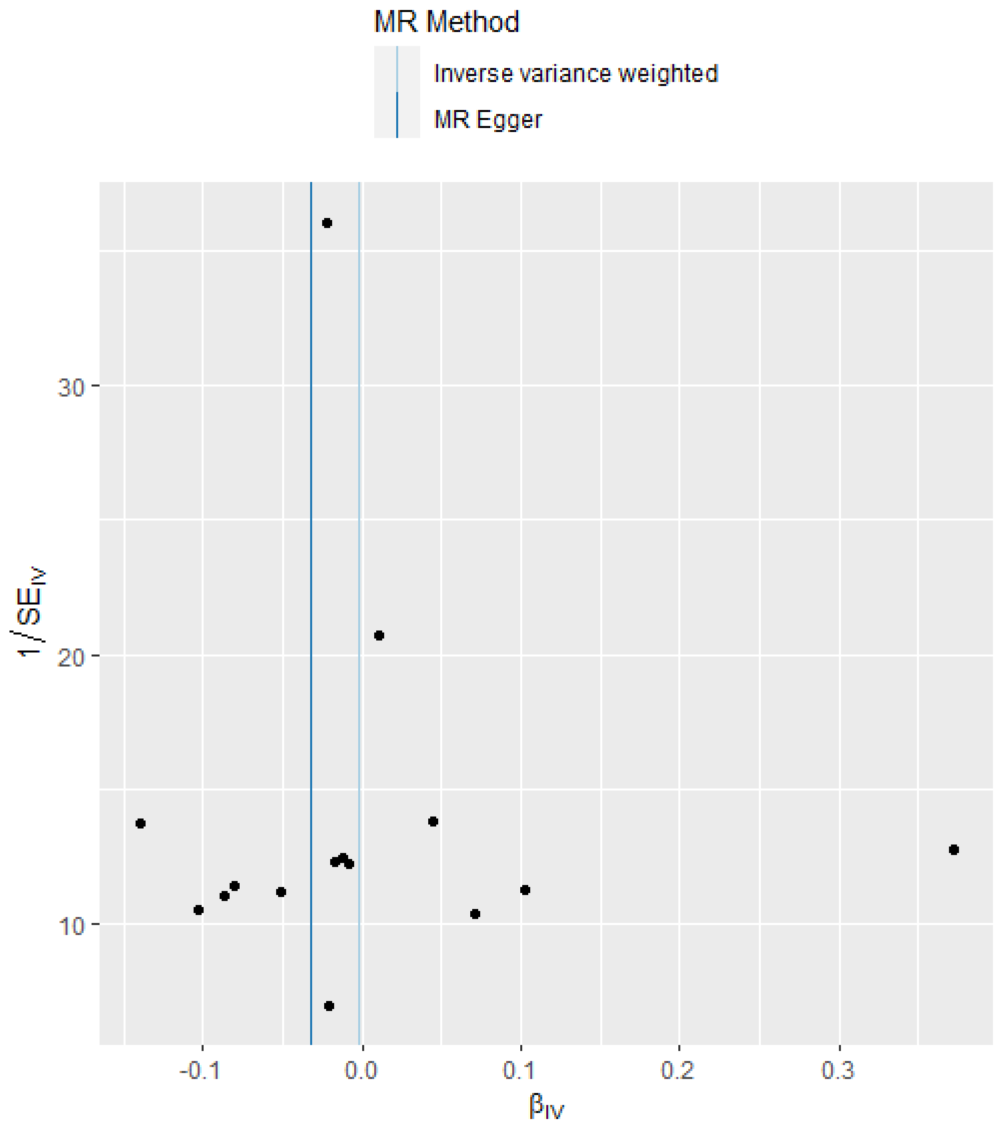

Figure 16 Funnel plot for ebi-a-GCST90014023

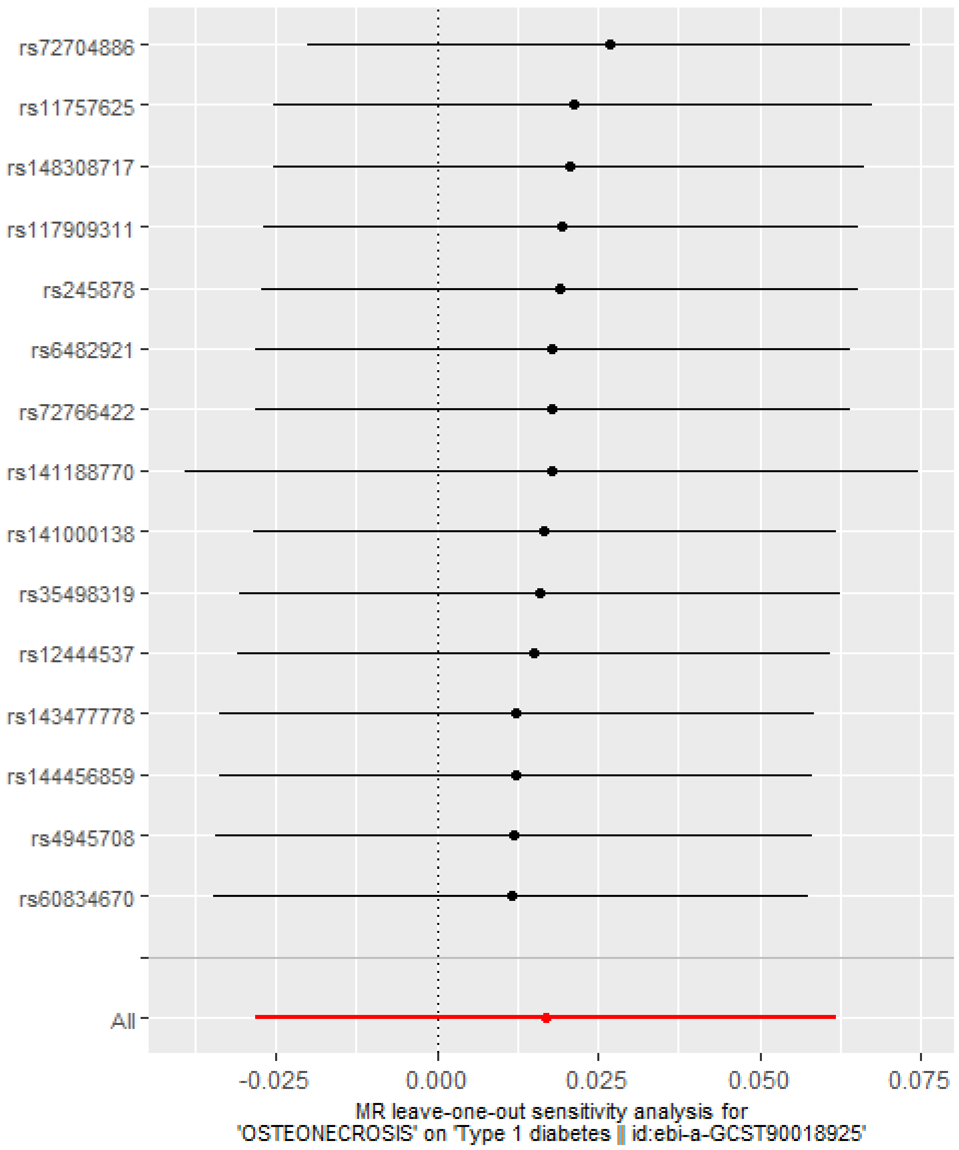

Figure 17 Leave-one-out method for ebi-a-GCST90018925

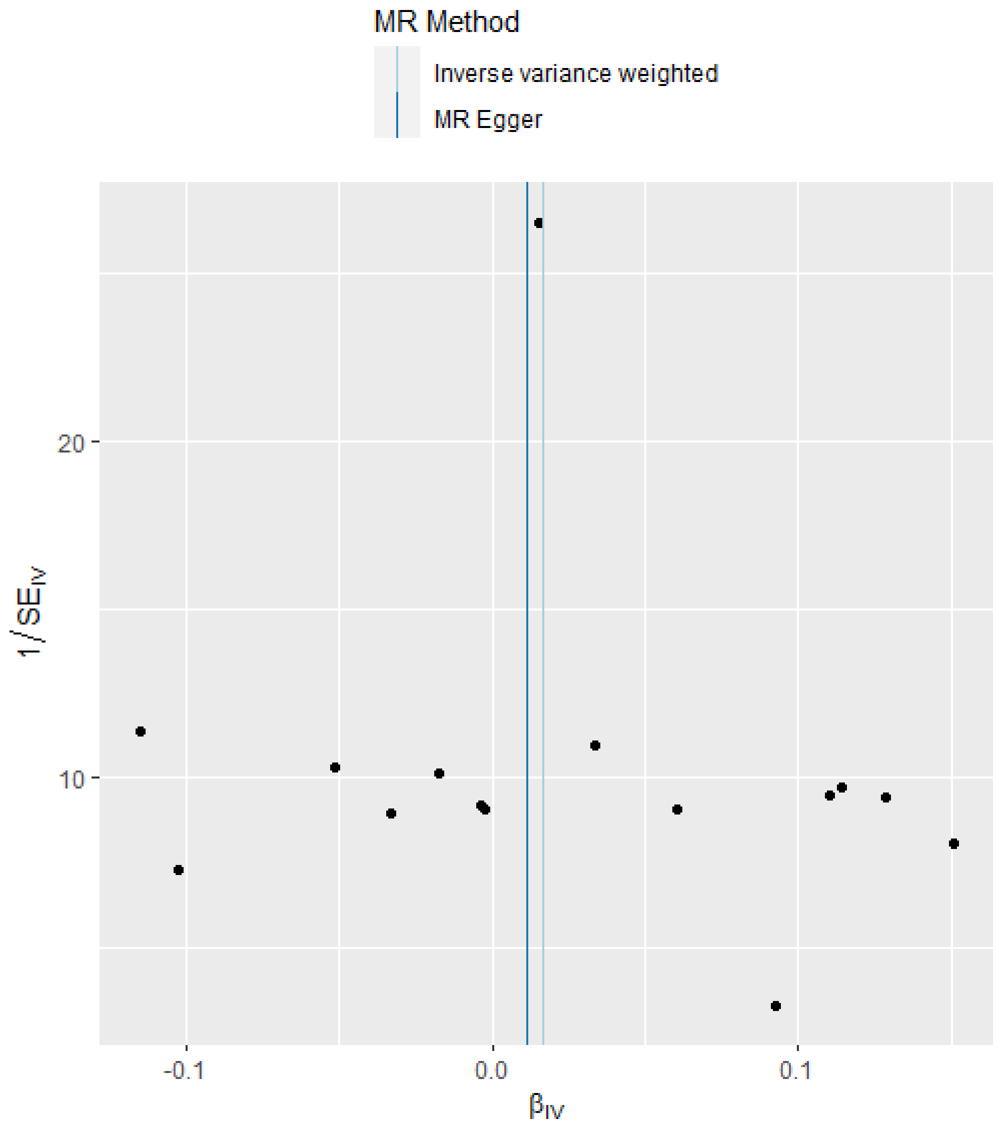

Figure 18 Funnel plot for ebi-a-GCST90018925
